# Can MXene be the Effective Nanomaterial Family for the Membrane and Adsorption Technologies to Reach a Sustainable Green World?

**DOI:** 10.1021/acsomega.3c01182

**Published:** 2023-07-24

**Authors:** Şirin Massoumılari, Sadiye Velioǧlu

**Affiliations:** † Institute of Nanotechnology, Gebze Technical University, Gebze 41400, Kocaeli, Turkey; ‡ Nanotechnology Research and Application Center, Gebze Technical University, Gebze 41400, Kocaeli, Turkey

## Abstract

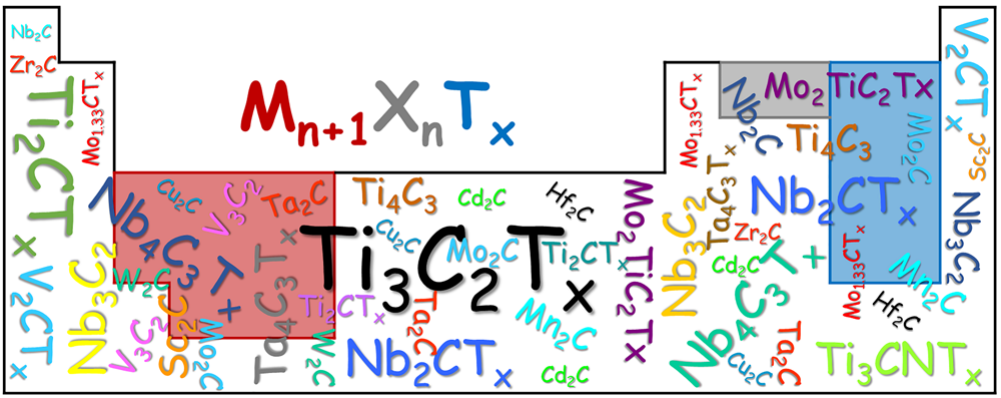

Environmental pollution has intensified and accelerated due to a steady increase in the number of industries, and exploring methods to remove hazardous contaminants, which can be typically divided into inorganic and organic compounds, have become inevitable. Therefore, the development of efficacious technology for the separation processes is of paramount importance to ensure the environmental remediation. Membrane and adsorption technologies garnered attention, especially with the use of novel and high performing nanomaterials, which provide a target-specific solution. Specifically, widespread use of MXene nanomaterials in membrane and adsorption technologies has emerged due to their intriguing characteristics, combined with outstanding separation performance. In this review, we demonstrated the intrinsic properties of the MXene family for several separation applications, namely, gas separation, solvent dehydration, dye removal, separation of oil-in-water emulsions, heavy metal ion removal, removal of radionuclides, desalination, and other prominent separation applications. We highlighted the recent advancements used to tune separation potential of the MXene family such as the manipulation of surface chemistry, delamination or intercalation methods, and fabrication of composite or nanocomposite materials. Moreover, we focused on the aspects of stability, fouling, regenerability, and swelling, which deserve special attention when the MXene family is implemented in membrane and adsorption-based separation applications.

## 1 Introduction

Environmental pollution originating from population growth, increased affluence, and the industrial revolution has increased quickly.^1^ As a result of the ruthless consumption of the resources, the ecological balance has been deteriorated.^2^ The main resources used dominantly are the nonrenewable fossil fuels such as coal, natural gas, and oil, formed over thousands of years.^3^ The amount of anthropogenic CO_2_ released to the atmosphere by the use of these resources between the years of 1750 and 2011 is approximately 2040 Gt, and 50% of this amount has been released only in the last 40 years.^4^ Unfortunately, the release of greenhouse gases causes the local temperature to increase. For instance, a 3.5 °C increase in an average world temperature corresponds to a 7 °C increase in the polar regions. The temperature rise leads to the ice melting at the poles and mountains, and hence, the sea level increases, which is expected to increase between 9 and 88 cm in 2100.^4^ Regrettably, besides the air pollution, soil contamination has reached alarming levels due to the release of heavy metals, solvents, pharmaceuticals, pesticides, fertilizers, radioactive wastes, and plastics.^5^ Similarly, the pollution risk also exists for water sources such as rivers, lakes, oceans, etc. It was expected that by 2025, half of the world’s population would face water scarcity due to the contamination of drinking-water sources with feces and the merger of clean water with sea water as a result of the rising sea level.^6^ Hereby, people all over the world will be forced to become a climate migrant. Moreover, biodiversity and the marine ecosystem are also under serious threat due to the rapid climate change and the acidification of the ocean.^7^ The direct impact of pollution on human health is recognized as the occurrence of 9 million premature deaths in 2019, of which 6.7 million were from air pollution and 1.4 million from water pollution. Premature deaths were distressingly recorded in low and middle-income countries dominantly, which was explained by the “climate justice” concept.^8^ Collectively, air, soil, and water pollution are interconnected like links in a chain, and human health is influenced from them, either directly or indirectly. To offer a remedy for the environmental pollution, a number of international meetings and conferences have been held since the 1970s.^4^ In addition, the foundation of the Intergovernmental Panel on Climate Change (IPCC) was established in 1988 by the decision of the United Nations General Assembly.^4^ The most important international agreement is the Kyoto protocol in which strict obligations for the reduction and limitation of greenhouse gases were introduced. The experiences gained from the provided agreements paved the way for the content of the Paris Agreement in which several strict targets were set.^4^ Its long-term goal is limiting the global warming to about 1.5 °C, which was assured by the every five-year meetings to evaluate what has been done so far.

More than 30,000 different chemicals are used in our daily lives. The number of pollutants originated from the combination of these chemicals is definitely countless nowadays.^9^ The major pollutants produced via reaction of these chemicals and dominantly encountered in the water are heavy metals, dyes, oils, plastics, herbicides, and pesticides.^10^ In order to provide water safety, physical, chemical, and biological methods are being used for water remediation. Physical methods such as sedimentation, degasification, and membrane are the simple ones. They are energy efficient methods, whereas their capacities are limited due to the clogging problem. Although they reveal a solid performance, generally chemical methods are preferred in industry. Highly used chemical methods are flocculation and coagulation, ozonation, chemical precipitation, and ion exchange. Although these methods are also simple and have low operational costs, their main disadvantage is the high sludge production and disposal.^10^ Besides water treatment processes, the development of gas separation processes are also essential in order to suppress environmental pollution. To keep environmental sustainability and restrict carbon emissions, commonly used gas separation technologies are absorption/amine scrubbing, cryogenic distillation, adsorption, and membrane-based separation.^11^ Though amine-based scrubbing and cryogenic distillation are conventional and effective methods, a high energy requirement is the major challenge faced during CO_2_ separation.^11,12^ As an alternative, adsorption- and membrane-based gas separation applications were provided. They have become pervasive in applications related to energy, food, water, biotechnology, etc. Adsorption based separation is an attractive method where it promotes the use of various adsorbent materials, applicability in multiple operation modes, and high adsorption capacity.^11^ Likewise, the reasons for the attention of membrane-based separation processes are that they have low energy consumption, facile operation, and are scalable and environmentally friendly. Considering the application of membrane technology in both water treatment and gas separation, it has been proven in the literature that its efficiency in separation was close to the order of other methods, and its energy consumption was very low compared to the others, making it stand out from others.^13−15^ Accordingly, membrane market size was supposed as approximately 7 billion dollars in 2021 globally.^16^ As environmental pollution continues to increase, the membrane market will become a huge and competitive platform and membrane technology will record a big leap.^17^ Similarly, adsorbent market size is also flourishing with the estimation of 3.9 billion dollars in 2020.^16^ The adsorption market is expected to thrive more in the future with the development of novel adsorbents having high recyclability and reusability, improvement in the process design, and applied advanced characterization methods.^12^

To date, various membrane and adsorbent materials were examined for the target separation applications. Mostly, polymeric membranes have been widely used due to their scalability, processability, and cost effectiveness. However, the newly developed nanoporous inorganic materials offer advantages in their use as membrane and adsorbent materials due to their uniform pores and narrow pore size distribution. Very recently, after the discovery of graphene in 2004, two-dimensional (2D) nanomaterials started to bring much attention to membrane and adsorption technologies. Especially in the membrane area, it deserves this intense interest due to leading an ultrathin membrane fabrication, which lowers the mass transfer resistance and improves the production. Moreover, in the adsorption process, ultranarrow and functionalized interlayer channels distinguish the solute particles according to their size and chemical properties, resulting in an increase in the particle removal rate. There are different 2D material families such as graphene oxide (GO), zeolite, MXene, transition metal dichalcogenide (TMD), metal organic framework (MOF), covalent organic framework (COF), etc. which offer great potential in both membrane and adsorption technologies. 2D materials used in adsorption or membrane-based separation applications are prepared in the form of either nanoporous or nanolaminates. To enhance the separation properties of 2D membranes or adsorbents, several strategies were developed such as adjusting interlayer channels, modifying surface properties via functionalization, intercalating various molecules (cations, solvents, organic molecules), tailoring the porosity, etc.^18^ These strategies are beneficial to handle with the trade-off between permeability and selectivity existing in membrane processes and between working capacity and adsorption selectivity existing in adsorption processes.^12^

Discovery of transition metal carbides and nitrides named MXene in 2011 had a deep impact on the world of materials science. MXene nanomaterials are synthesized from MAX phases formulated *M*_*n+*1_*AX*_*n*_ (*n* = 1, 2, 3, 4) that comprise M, A, and X, representing early transition metals (Sc, Y, Ti, Zr, Hf, V, Nb, Ta, Cr, Mo, W, and Mn), IIIA or IVA group elements, and C and/or N, respectively.^19^ MAX phase which is the laminar hexagonal structure has the form of stacked transition metal carbide or nitride sheets in which X atoms are glued within A layers.^20^ Initially, the MXene (e.g., Ti_3_C_2_T_*x*_) fabrication method was the immersion of the MAX phase (e.g., Ti_3_AlC_2_) into the hydrofluoric acid (HF) at room temperature, which is named as the etching process. Thereby, the bonds between transition metals and A elements deteriorated, and A elements were removed.^21^ Within the early years of its discovery, MXene members of Ti_2_CT_*x*_, (Ti,Nb)_2_CT_*x*_, (V,Cr)_3_C_2_T_*x*_, Ti_3_CNT_*x*_, and Ta_4_C_3_T_*x*_ are included in the MXene family via the fabrication of the HF etching method.^22,23^ In 2013, the isolation of MXene as a single sheet by intercalating the organic molecules, which is known as the delamination of layers, was one of the most important turning points in the history of MXene nanomaterials. Instead of using a dangerous HF solution in etching, the use of acid mixtures with fluoride salts emerged in 2014 and single sheets of Ti_3_C_2_T_*x*_ with a small lateral size was obtained.^23^ After realizing that MXene nanomaterials have synthesis method dependent properties, various etching approaches were examined such as molten salt,^24^ Lewis acids,^25^ and electrochemical etching.^26^ With the help of evolutionary steps in MXene nanomaterials, their different forms such as MXene with in-plane and out-of-plane ordering of the metal atoms, in-plane vacancies of metal atoms, solid solution of metal or carbon/nitrogen atoms, and various surface terminations were synthesized and members of the MXene family increased rapidly.^23,27^ In 2011, there were only 70 MAX phases, whereas this number has reached 150, leading to the observation of a theoretically unlimited number of MXene structures.^20^

Attempts to adjust etching-delamination protocols or use alternative synthesis methods provided researchers with a way to obtain different MXene members in the above-mentioned forms, which enabled scientists to diversify the physical properties of the MXene family. For instance, the mechanical strength and stiffness of Ti_3_C_2_T_*x*_ nanosheets were examined by in situ transmission electron microscopy (TEM) probing and atomic force microscopy (AFM) nanomechanical mapping^28^ and displayed its superiority both experimentally and theoretically.^29,30^ The highest adhesion energy was identified between the nanosheets of Ti_3_C_2_T_*x*_ regardless of the layer number compared to the other 2D nanomaterials such as graphene, MoSe_2_, and SiO_2_.^31^ It was realized that depending on the each element within its structure, its electronic conductivity could be altered. MXenes having transition metals of chromium, molybdenum, and tungsten were defined as topological insulators,^21,32−37^ while Ti_3_C_2_T_*x*_ was accepted as the greatest conductor.^38^ However, Ti_3_C_2_T_*x*_ terminated with the groups of −F_2_ and −(OH)_2_ displayed the attitude of semiconductors with narrow band gaps, contrary to the one terminated with −O_2_ groups.^39^ Promisingly, Ti_3_C_2_T_*x*_ revealed high temperature stability up to 500 °C under argon atmosphere, with a formation of TiO_2_ crystals onto the edges.^40^ However, Zr_3_C_2_T_*x*_ kept its structure up to 1000 °C under vacuum.^41^ Considering the superb properties of already existing MXene members such as high mechanical strength, adhesion, electrical conductivity, and thermal stability, depending on their application of interest, further improvements on novel MXene members can be obtained by only manipulating their precursor, composition, and synthesis methods.

The tuneability of MXene properties has been studied from different perspectives. It was proved both experimentally and theoretically that optical and electronic properties of MXene nanomaterials having a M_2_CT_*x*_ (M = Ti, Nb, and V) structure can be altered depending on the M-site as well as its composition.^42^ For instance, Halim et al.^43^ reported that Ti_2_CT_*x*_ and Nb_2_CT_*x*_ displayed different optical properties due to their inevitable discrepancy in electronic configuration and bonding between the carbon and transition metal. It was also suggested for a large number of structures and corresponding compositions as M_2_XT_*x*_, M_3_X_2_T_*x*_, and M_4_X_3_T_*x*_ that both their electrical conductivity and electromagnetic interference shielding (EMI) performance could be adjusted in a broad range.^44^ Additionally, it was displayed on 62 different MXene structures formulated in the form of (M′_2/3_M″_1/3_)_2_X that magnetic properties can be easily varied because of their geometries.^45^ Beyond mechanical, optical, electrical, and magnetic properties, MXene nanomaterials have exceptional antibacterial activities. Antibacterial performance of Nb_2_CT_*x*_, Nb_4_C_3_T_*x*_, Ti_3_C_2_T_*x*_, and Ti_2_CT_*x*_ were compared by the group of Mahmoud.^46^ Nb_2_CT_*x*_ displayed lower antibacterial efficiency than Nb_4_C_3_T_*x*_ against the Gram-positive (*S. aureus*) (96%) and negative bacteria (*E. coli*) (94%), which were slightly lower than Ti_3_C_2_T_*x*_ nanosheets (98%).^46^ Tunable structure–property relation of MXene nanomaterials provides many advantages for various applications of interest.

Besides the outstanding properties of MXene nanomaterials, there are some challenges associated with their structures, which restrict their use in separation applications. The main limitation is their restricted stabilities under different conditions. It was revealed that Ti_3_C_2_T_*x*_ heated at different temperatures in air lost its structure stability very rapidly and TiO_2_ nanocrystals were observed in thin sheets of disordered graphitic carbon.^47^ The corresponding oxidation rate of MXene under different conditions was often explored in the literature.^48^ The strongest MXene oxidation rate was observed in a H_2_O_2_ environment, while the weakest rate was recorded in dry air.^48^ To tackle with the fast oxidation rate of MXene, improvement in either the synthesis procedure of MXene in order to eliminate the defects or its storage conditions is required. Since the defective sites on or at the edge of the MXene nanosheets become susceptible to the oxidative degradation, novel synthesis approaches were aimed to be developed.^49^ Mathis et al.^50^ just modified the MAX phase of Ti_3_C_2_T_*x*_ and increased its structural stability in closed vials up to the 10 months at room temperature. Barsoum et al.^51^ deactivated the possible interaction between dissolved oxygen in water and the edge of MXenes (Ti_3_C_2_T_*x*_ and V_2_CT_*x*_) via polyanionic salts and prevented oxidation. Alternatively, storage conditions such as temperature, pH, and concentration of colloidal dispersion of MXene were examined. Athavale et al.^52^ reported a comprehensive survey of their group’s progress in mitigating the degradation of MXenes via low-temperature storage and the use of antioxidants. Zhang et al.^53^ proposed storing conditions for the colloidal dispersion of Ti_3_C_2_T_*x*_ as Ar-filled bottles at 5 °C where chemical stability was improved from days to months. Zhao et al.^54^ replaced water with a suitable solvent as a storage medium in order to protect nanosheets from oxidation. 1-(3-aminopropyl)-3-methylimidazolium bromide [type of ionic liquid (IL)] exfoliated Ti_3_C_2_T_*x*_ and revealed a stability of up to 80 days even after exposure to water.^54^ Not only the stability but also the restacking problem of nanosheets were prevented by the use of ILs as solvents for the storage and dispersion of MXene nanosheets. Other limitations of its use in separation applications is the restacking of MXene nanosheets. To keep the stacking distance (nanoconfined space) between nanosheets constant, MXene structures were delaminated via various intercalants such as solvents, ions, organic molecules, other layered materials, and polymers.^55^ By altering the size of intercalant molecules, it was targeted to tailor the interlayer distance of MXene. Alternatively, it was utilized from the interaction capability of intercalants with the MXene surface in order to create a synergy between them.^55^ Munir et al.^56^ identified the effect of etchant concentration, solvent type, and sonication time on the adjustment of interlayer distance of MXene nanomaterials. They demonstrated that 30% HF etchant, dimethyl sulfoxide (DMSO) as a solvent, and 135 min of sonication time were the best parameters to synthesize MXene with the highest interlayer spacing.^56^ Since the implementation of the MXene nanomaterial family has increased for a variety of applications, its toxicity becomes another constraint. Therefore, its biotoxicity was aimed to be identified for several normal and cancer cell lines. It was reported that cell viability of the normal cell line (HaCaT) was over 80% in the presence of Ti_3_C_2_T_*x*_, even with the highest concentration of 500 mg/L.^57^ Nb_2_C modified with polyvinylpyrrolidone (PVP) was inserted into a mouse model, and no adverse effects were recorded, supported by the biochemistry and hematological tests. Similarly, Ti_3_C_2_T_*x*_ inserted into the zebrafish embryo model with a concentration of up to 100 mg/L did not lead to any adverse effects.^58^ The influence of not only the MXene concentration but also its exposure time, lateral size, and functional groups on cytotoxicity should be investigated. Additionally, even though toxicity studies are increasing day by day, more studies are required for different types of MXene in vivo.

To adopt the MXene family in membrane or adsorption technologies, other criteria that need to be figured out are the adjustment of surface termination groups, sheet size, surface area, and defect ratio. Therefore, novel etching or delamination routes were proposed. Since 2011, dozens of MXene nanomaterials have been prepared via the main MXene synthesis approach of the HF-etching route. Even it is the effective way to fabricate high quality of MXene nanosheets, due to the safety and environmental impact, different etching, and delamination processes to fabricate MXene from the MAX phase performed to date. Alternative to the HF-etching method, it was reported that the LiF + HCl route also provided high quality MXene nanosheets.^59^ Differently, Ti_4_N_3_T_*x*_ by fluoride molten salt,^24^ fluorine free MXene by hydrothermal alkali etching,^60^ Ti_3_C_2_T_*x*_ from non-Al MAX of Ti_3_SiC_2_ by HF-H_2_O_2_ as etchant,^61^ and hardly obtained MXenes by Lewis acidic molten salt^25^ were fabricated in a high quality form. On the other hand, covalent modification of surface terminations of MXene,^62^ delamination of titanium- and niobium-based MXenes with urea and amine,^63,64^ delamination of Ti_3_CNT_*x*_ with tetrabutylammonium hydroxide (TBAOH),^65^ use of lithium salts,^66^ in-plane ordered vacancy (Mo_1.33_CT_*x*_) MXene synthesis with the improved etching and delamination methods,^67^ and etching-delamination strategies of UV-induced^68^ and chemical-combined etching^69^ were applied successfully without leading any structural deformations. And it seems that new methods will continue to be developed to meet the criteria for the target of applications.

Especially, for membrane applications, MXene nanosheets are mostly casted in a film form by processing the MXene solution.^70^ One of the ways for processing the MXene solutions is the printing methods such as inkjet, screen, and extrusion, which provide a large scale and low-cost film fabrication, and controlled MXene deposition.^70^ However, there are some challenges due to the use of low viscosity of water-based solvents in printing methods as the clogging of nozzles of the printer. Recently, with the help of synthetic structural proteins as binder molecules and DMSO as solvent, novel inks were develop and printed onto the various substrates containing polyethylene terephthalate (PET), poly(methyl methacrylate) (PMMA), glass, and cellulose paper.^71^ Surprisingly, the MXene solution containing multilayered Ti_3_C_2_T_*x*_ and access to the MAX phase after the delamination step was used as ink for screen printing.^72^ Although the thickness of the MXene film was lower than that obtained via inkjet printing, using MXene sediment-based ink named “trashed” emerged as a green and sustainable fabrication method for the fabrication of MXene films.^72^ Hybrid inks consisting of specific cellulose nanofibrils and Ti_3_C_2_T_*x*_ were applied via 3D extrusion printing on a flexible smart textile.^73^ Comparing these printing methods, fabrication yield of screen printing is mainly higher than inkjet and extrusion printing methods.^70^ The other way for processing the MXene solutions to form a film is the coating techniques such as dip, spin, blade, and spray coatings. Dip and spin coatings enabled a film obtained with the lowest thickness and good conductivity for the MXene dispersion having the viscosity range between 1 and 100 mPa × s.^74^ Importantly, good alignment of nanosheets was provided via spin coating as a result of the applied centrifugal force.^75^ However, its production yield was lower than the other coating techniques. As the viscosity of the MXene dispersion surpassed 1000 mPa × s, blade coating became suitable to create the MXene film with a large film area.^76^ However, thick film thickness was observed compared to the films fabricated via the dip and spin coating methods. Fabrication throughputs of coating techniques were reported as follows, spray > blade > dip-spin coatings.^70^ The highly preferred way in laboratory conditions for processing the MXene solutions to form a film is the filtration methods such as vacuum- and pressure-assisted filtration methods. In vacuum-assisted filtration methods, the MXene dispersion is filtered through a porous filter substrate under vacuum to form a free-standing MXene layer.^77^ Film thickness can be adjusted by varying the concentration of MXene in dispersion and filtration volume. Moreover, film formation can be influenced from the vacuum speed, the choice of solvent and substrate membrane, viscosity of dispersion, etc.^70^ The highest specific surface area (SSA) was reported as 575 cm^2^ for Ti_3_C_2_T_*x*_, prepared by an electrophoretic deposition method,^78^ not vacuum filtration. Although a vacuum-assisted filtration method is easy to control, it has a large scalability problem in order to be applied in membrane industry.

The MXene family with their tremendous properties such as high surface area, hydrophilic manner, high metallic conductivity, active surface sides with tailorable functional groups, tunable interlayer spacing, and surface chemistry has been garnering increasing attention from several industries. MXene nanomaterials were practiced in widespread use in different applications such as energy storage,^79,80^ supercapacitors,^81^ hydrogen storage,^82^ catalysis,^83^ ultrafast photonics,^84^ membrane-based separation technologies,^85−94^ biomedical engineering, biosensors,^95,96^ and memristive and tactile sensory systems.^97^ Unlocking the full potential of MXene has revealed the need for mass production. Promisingly, Gogotsi and colleagues^98^ synthesized Ti_3_C_2_T_*x*_, for small (1 g) and large (50 g) batch sizes in a reactor (1 L) they designed. To synthesize a large batch of MXene, the protocol used to synthesize the small batch size was scaled up easily without any adjustment in conditions. Interestingly, very recently, Chen et al.^99^ used a supercritical etching method assisted by supercritical carbon dioxide for the mass production of Ti_3_C_2_T_*x*_, Nb_2_CT_*x*_, Ti_2_CT_*x*_, Mo_2_CT_*x*_, and Ti_3_CNT_*x*_ with the yield of ∼1 kg within ∼2–5 h. These are inspiring studies that pave the way for the scalability of MXene synthesis in order to observe industrial quantities.^98,99^ However, more studies are required to reach above the kilogram scale.

Since the MXene nanomaterial family provides outstanding physicochemical and structural properties, it attracted considerable attention in different applications. Therefore, several critical reviews have been published dominantly focusing on its separation applications.^86,90,91,94,100−124^ However, most of them could not cover such a large number of separation applications and aimed to concentrate on specifically either the membrane^90,100−102^ or adsorption^94,103−108^ based separation processes. However, there are comprehensive review studies covering the potential of MXene membranes and adsorbents for a single separation application such as pervaporation,^109^ gas separation,^110−112^ desalination,^86,113,114^ dye removal,^91,115^ oil-in-water separation,^116^ removal of heavy metal ions, and radionuclides.^115,117−120^ On the other hand, few studies^108,121,122^ aimed to highlight the effect of several strategies such as various surface modification approaches of MXene, intercalation methods to enhance interlayer distance of MXene nanochannels, etc. on the improvement of separation properties of MXene-based membranes and adsorbents for several practical applications. Additionally, there are also few studies that only discussed the specific modification approaches applied for the MXene family (intercalation chemistry^123^ and surface functionalization^124^) to improve its potential in different fields.

**Figure 1:**
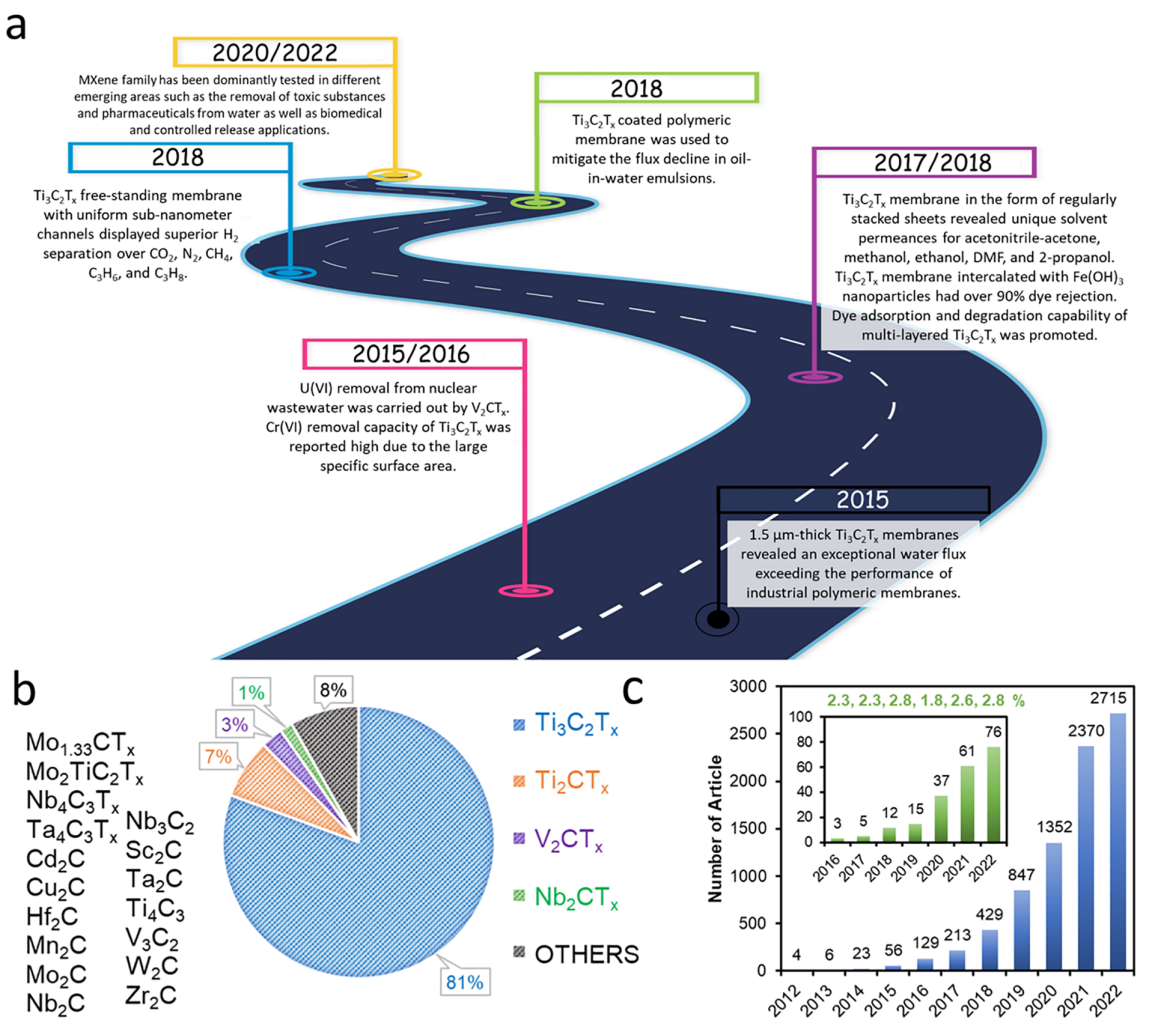
Literature survey of MXene nanomaterials. (a) Milestones of each separation application,^125−143^ (b) distribution of MXene types that were investigated for the separation application (other MXene types were listed on the left side of the figure), and (c) growth of the MXene-based studies in all fields on the Web of Science database reported on December of 2022 (reviews and patents were excluded). Inset figure of panel (c) represents the growth of the MXene literature in only separation applications, along with its percentage over all fields in each year.

Addressing the lack of adequate and safe water and impact of climate change is among the most important challenges of our time. Herein, we focused on the evaluation of performance of the most cost-effective, high performing, easy implementable membrane- and adsorption-based separation processes to suppress these challenges. As a being potential candidate nanomaterial family, successful implementation of MXene was revealed in membrane- and adsorption-based applications, namely, gas separation, solvent dehydration, dye removal, separation of oil-in-water emulsions, heavy metal ion and radionuclides removal, desalination, and some other prominent applications. In each section, the inspiring article in which the MXene nanomaterial was applied to that application was given initially, and then it was followed by compiling the considerable progress that has been made in each application. Information of inspiring articles for each application is depicted in Figure 1(a) along with a timeline manner. Additionally, it was also aimed, on the one hand, to prove the applicability and the effectiveness of MXene nanomaterials to several separation applications by tabulating their performances and, on the other hand, to unroll the use of a limited number of MXene members, instead of increasing the number of studies evaluating the MXene nanomaterials for different areas. Up to now, intensive efforts have been devoted to the use of the Ti_3_C_2_T_*x*_ member from the MXene family for the provided diversified separation applications, as summarized in Figure 1(b), although there are an abundant number of MXene members identified. However, as illustrated in Figure 1(c), a tremendous increase was observed in the applications of MXene nanomaterials for various fields since its discovery in 2011. Additionally, in this review, we provide an up-to-date overview of the major achievements on bio/fouling, swelling, regenerability, and long-term stability, which offer great promise to enable the proliferation of the MXene nanomaterial in separation applications. Collectively, we have highlighted not only the separation performance of the MXene family but also its possible applications to industry by discussing them in terms of different perspectives. Considering that only a few of the MXene have been revealed to date, researchers have a long way to go in the near future to thoroughly test them all for a variety of separation applications.

## 2 Gas Separation

**Figure 2:**
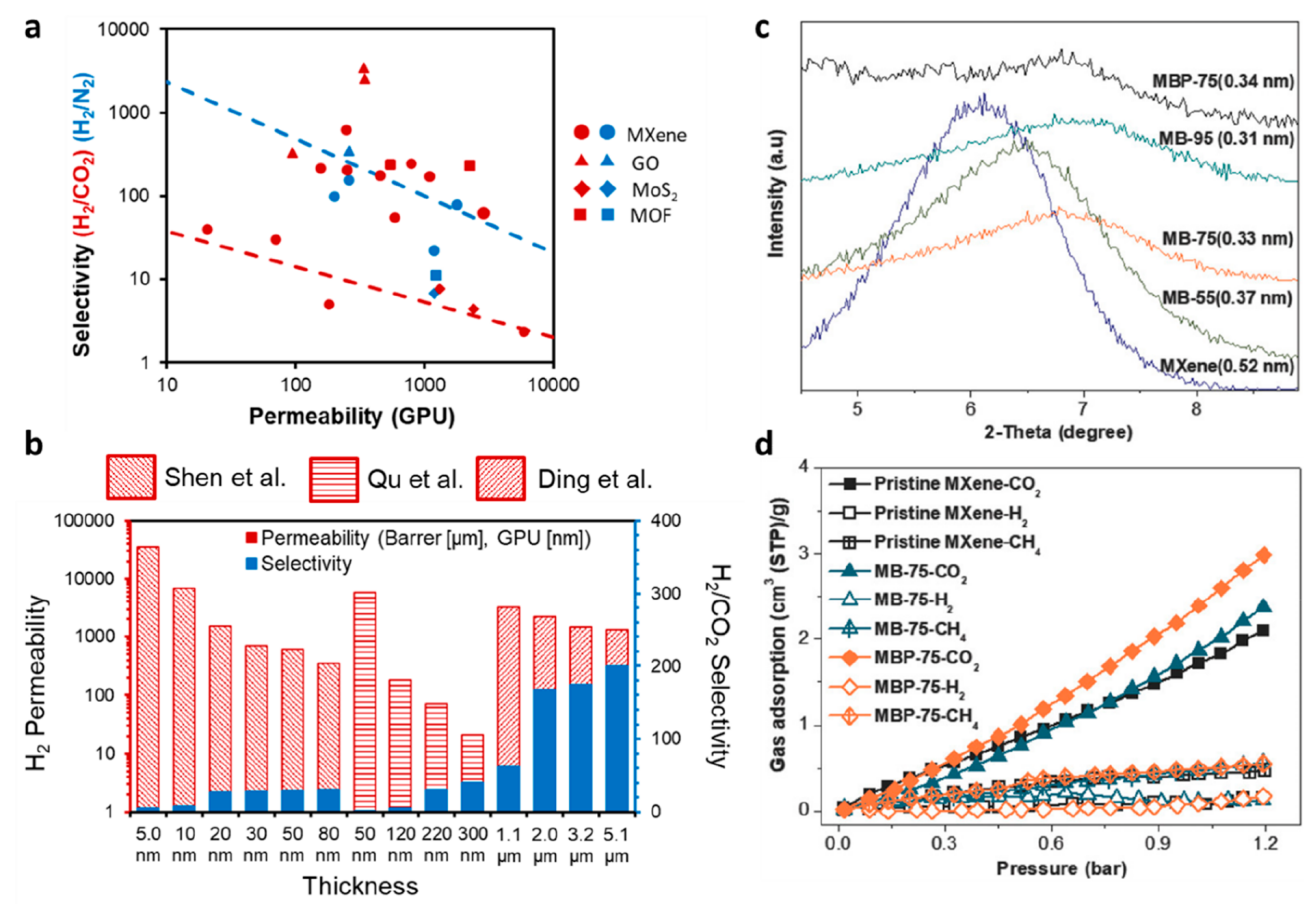
Gas separation performance of MXenes. (a) Comparison of H_2_/CO_2_ and H_2_/N_2_ separation performances of MXene membranes proposed by Ding et al.,^129^ Fan et al.,^144^ Wang et al.,^145^ Qu et al.,^146^ and Fan et al.^147^ with the other nanomaterial membranes. The red and blue lines denote the 2008 upper bound of the polymeric membrane for H_2_/CO_2_ and H_2_/N_2_, respectively, assuming membrane thickness is 0.1 μm. (b) H_2_ permeability and H_2_/CO_2_ selectivity of MXene membranes as a function of membrane thickness, which were measured by Ding et al.,^129^ Qu et al.,^146^ and Shen et al.^153^ (c) Analysis of the interlayer spaces of pristine and functionalized MXene nanofilms using XRD spectra. MB and MBP refer to the MXene-borate and MXene-borate-PEI composites, respectively. Adapted with permission from ref (153). Copyright 2018 Wiley Online Library. (d) Comparison of gas adsorption on pristine and functionalized MXenes at 25 °C. Adapted with permission from ref (153). Copyright 2018 Wiley Online Library.

With regard to the high demand for technologies in the context of sustainability and resource recovery, membrane and adsorption technologies attracted eminent attention as the major paradigm for gas separation in the past few decades. For gas separation application, the first MXene-based membrane was fabricated very recently by Ding et al. in 2018.^129^ Ding et al.^129^ synthesized MXene (Ti_3_C_2_T_*x*_) nanosheets with the uniform subnanometer channels and used these channels as blocks to create 2D laminated membranes with different thicknesses for the investigation of H_2_ separation. They reported that while single H_2_ permeability of the MXene membrane was on the order of 2400 Barrer, its binary H_2_ permeabilities for H_2_/CO_2_, H_2_/N_2_, H_2_/CH_4_, H_2_/C_3_H_6_, and H_2_/C_3_H_8_ mixtures were 2227, 1976, 1931, 2312, and 2357 Barrer, respectively.^129^ In addition to its superior single and binary H_2_ permeation coefficients, they also revealed that it had an ideal selectivity of 238 and a separation factor of 167 for H_2_/CO_2_.^129^ To further estimate the reason behind this inspiring H_2_ separation performance of MXene membrane, they used molecular simulation techniques and ascribed to the channel orderliness. As a result of this study, among all 2D and three-dimensional (3D) inorganic materials synthesized up to now, MXene with the thickness of 2 μm has emerged as the best performing material with H_2_ permeability of 2267 Barrer and H_2_/CO_2_ selectivity of 167 for the binary gas mixture, as illustrated in Figure 2(a).^129^ Its heart’s beating location in Robeson’s trade-off diagram for H_2_/CO_2_ separation accelerated the research about gas separation performance of MXene within five years. After the first encouraging and exciting results came from Ding et al.^129^ about H_2_/CO_2_ separation of MXene membranes, other studies aroused targeting different gas pairs like the study of Fan et al.^144^ focusing on H_2_/N_2_ separation. Fan et al.^144^ similarly fabricated 2D lamellar MXene (Ti_3_C_2_T_*x*_) membrane for the investigation of H_2_/N_2_ separation. While single H_2_ and N_2_ permeabilities were reported as ∼4.05 × 10^–7^ (968 Barrer) and ∼1.84 × 10^–8^ (44 Barrer) mol/m^2^ × s × Pa, those for binary mixtures were measured as ∼3.99 × 10^–7^ (954 Barrer) and ∼0.37 × 10^–8^ (8.76 Barrer) mol/m^2^ × s × Pa, respectively. Ideal and binary H_2_/N_2_ selectivities of the MXene membrane were measured as ∼22 and 41, respectively.^144^ Compared with the single H_2_ permeability and ideal selectivity (129) of MXene membrane fabricated by Ding et al.^129^ for H_2_/N_2_ separation, the one provided by Fan et al.^144^ revealed an order of magnitude greater permeability, but an order of magnitude lower H_2_/N_2_ selectivity, as given in Figure 2(a). Ding et al.^129^ fabricated 2D lamellar MXene by etching with hydrogen chloride (HCl) + lithium fluoride (LiF) solutions, which provide high exfoliation yields. However, Fan et al.^144^ synthesized 2D lamellar MXene via HF etching method and used DMSO as an intercalating substance, leading to the enhanced interlayer distance between MXene laminates. To reveal the gas separation performance of MXene membranes, we compiled the data of free-standing MXene membranes in the early published studies and plotted them on the Robeson diagram as illustrated in Figure 2(a). Concerning the H_2_/CO_2_ and H_2_/N_2_ separation performances of MXene membranes,^129,144−147^ they revealed H_2_/CO_2_ separation well above the upper bound and H_2_/N_2_ separation in the range of upper bound line exceeding the other 2D membranes. Mainly, this performance was attributed to its subnanometer interlayer spacing between the well-aligned and regular MXene nanosheets, which behave as a molecular sieving membrane.

In addition to the membrane-based separation process, MXene nanomaterials were also used as adsorbents for the capture of different targeting gases such as CH_4_,^148,149^ CO_2_,^150,151^ formaldehyde (HCHO),^152^ etc. Zhang et al.^152^ demonstrated the stability of Ti_3_C_2_O_2_ up to 177 °C and its high HCHO adsorption capacity (6.9 mmol/g) in their computational study where density functional theory (DFT)-based ab initio molecular dynamics simulation was carried out. They proposed that interfacial van der Waals (vdW), H–O, and C–O interactions were responsible for gas capture in MXene adsorbents. These promising studies have enlightened the importance of exploring the gas separation performance of MXene nanomaterials for the membrane- and adsorption-based technologies. Since they were very recently discovered for gas separation applications in membrane and adsorption technologies, the mechanism underlying their outstanding separation performance needs to be investigated. Therefore, several issues were considered in the literature such as rationally tailoring its 2D channels by either tuning thickness or intercalating specific species between its nanochannels to optimize the separation properties. The studies identifying the MXene nanomaterials as membranes and adsorbents tried to answer the question of “Can better gas separation performance be achieved by the help of MXene nanomaterials?”

### 2.1 Membrane-Based Separation

Gas transport mechanism within the membrane can be affected by various parameters especially physicochemical properties of the membrane material. Membrane structure and thickness, nonselective defects/voids, and galleries between nanosheets can change the transport rate of gas molecules. Interlayer spacing was suggested as the most important factor affecting the gas transport in lamellar membranes due to the tunable channel width. To investigate the degree of effect of interlayer spacing on gas separation performance of MXene membranes, several studies were carried out in the literature.^129,144,153−155^ Ding et al.^129^ varied the thickness of the MXene membrane with 0.2, 0.5, 1.1, 2, 3.2, and 5.1 μm to show the relationship between membrane thickness and H_2_ permeability and H_2_/CO_2_ selectivity. H_2_ permeability was decreased with the membrane thickness from 2961 Barrer of 0.2 μm-thick membrane to 1302.7 Barrer of a 5.1 μm-thick one, while H_2_/CO_2_ selectivity was increased from ∼4.5 to 200 as given in Figure 2(b). Likewise, Shen et al.^153^ revealed the effect of membrane thickness on H_2_ permeance and H_2_/CO_2_ selectivity using ultrathin MXene (Ti_3_C_2_T_*x*_) lamellar membranes having the thickness of 0.005, 0.01, 0.02, 0.03, 0.05, and 0.08 μm (change in interlayer spacing from 15 to 11.6 Å). Similar gas separation performance alteration of the thick MXene membranes fabricated by Ding et al.^129^ was revealed by the ultrathin membranes synthesized by Shen et al.^153^ where H_2_ permeances and H_2_/CO_2_ selectivities of MXene membranes were changed from ∼36,000 to 350 GPU and from ∼5 to 31, respectively, as given in Figure 2(b). The most severe drop in H_2_ permeances (from 6000 to 3 GPU) and similar increase in H_2_/CO_2_ selectivities (from 2.32 to 30.3) were reported by Qu et al.^146^ accompanying with the increase in thickness from 0.05 to 0.22 μm (decrease in interlayer spacing from 4.0 to 3.1 Å) for the self-cross-linked MXene (Ti_3_C_2_T_*x*_) membranes by heat treatment at 140 °C for 10 h. The reverse relation of membrane thickness with interlayer spacing was suggested, and therefore, the importance of interlayer distance on gas separation was underlined by these studies. To clarify the proposed claim and explain the gas separation mechanism, Ding et al.^129^ carried out molecular dynamics (MD) simulations for MXene nanosheets having two different interlayer spacing and found that small disturbances of nanochannels regularity affected the interlayer spacing leading to the deteriorated gas selectivity. While H_2_/CO_2_ selectivity of the MXene membrane with the interlayer distance of 4.5 Å was computed as only ∼70, that of MXene membrane with interlayer distance of 3.5 Å was >200.^129^ In accordance with these studies, the decrease in gas permeability and increase in selectivity with the membrane thickness was related to the prolonged diffusion pathway due to the shrinkage of interlayer spacing. Interlayer spacing was influenced not only by the membrane thickness but also by the membrane film treatment conditions, such as temperature, which had a dominant influence on the change of interlayer spacing. Fan et al.^144^ identified the effect of high temperature treatment on H_2_/N_2_ separation of MXene membrane. For mixed gas permeation measurements, H_2_ permeance and H_2_/N_2_ separation factor of a MXene membrane treated at 320 °C were reported as 612 GPU and 41, respectively, whereas those treated at 20 °C were 690 GPU and 12.^144^ Since its interlayer spacing decreased from 13.4 to 12.8 Å with the temperature increase from 20 to 300 °C, an enhanced molecular sieving was observed yielding the improvement in the H_2_/N_2_ separation factor.^144^ The most tremendous decrease in the interlayer spacing was observed from 3.45 to 0.24 Å by the study of Emerenciano et al.^155^ with treatment of the Ti_3_C_2_T_*x*_ membrane at 500 °C under an Ar/H_2_ atmosphere, compared to the one treated at 80 °C under vacuum.

On the other hand, since the best way to identify the underlying mechanism behind the interlayer spacing is to carry out molecular simulations, Li et al.^154^ examined in depth the diffusion of several gas molecules (He, H_2_, CO_2_, N_2_, and CH_4_) for anhydrous and hydrous MXene (Ti_3_C_2_O_2_) nanosheets by varying the interlayer spacing. Their results supported the above-mentioned experimental studies revealing the significant effect of interlayer spacing between MXene nanosheets on gas transport. They proposed that for small interlayer spacing, gas molecules diffused close to the MXene walls, whereas for large interlayer spacing, diffusion was affected from the mass of the gas molecule instead of its kinetic diameter.^154^ Li et al.^154^ suggested that hydrous MXene ranging between *d*-spacing of 6 and 8 Å and anhydrous MXene having *d*-spacing around 5 Å displayed the optimum gas separation performances based on the criteria of H_2_/CO_2_ diffusion selectivity >70 and H_2_ diffusion >7 × 10^–5^ cm^2^/s. Jin et al.^156^ investigated the thickness of MXene on H_2_/N_2_ separation by using two different nanochannel models, flatwise nanochannels (>20 Å) and corrugated nanochannels (<20 Å), which were formed with nonregularly distributed MXene nanolayers. Similarly, as the thickness of MXene reduced from 1 to 0.02 μm, H_2_ and N_2_ permeances increased from 477.6 to 4090 GPU and from 24.2 to 201.5 GPU, respectively. However, interestingly, H_2_/N_2_ selectivity kept stable as 13.5. It was ascribed to the stability of the fractional contribution of Knudsen diffusion and the molecular sieving mechanism as 18% and 82%, respectively. Therefore, according to their model, they suggested that the thickness of the MXene membrane did not have any effect on the fractional diffusion of gases. While studies on the effect of interlayer spacing on gas separation of MXene membranes continue, on the other hand, new approaches have been sought to enhance selectivity without sacrificing from permeability. Considering the adsorption process, in our recent atomistic scale simulation study,^157,158^ we have targeted to quantitatively identify the alteration of the CO_2_ working capacity and CO_2_/H_2_ adsorption selectivity of 730 MXene adsorbents after the enhancement in the interlayer distance. Therefore, adsorption simulation of the proposed MXene database was carried out for structures having two different interlayer distances (3.5 and 6.5 Å). We recorded a considerable decrease in both CO_2_/H_2_ adsorption selectivity and CO_2_ working capacity of 76% of the total MXene structures in numbers.^157,158^ As a result of electrostatic and vdW interactions, CO_2_ can be easily kept within the MXene nanochannels at the short interlayer distance, and the effective gas adsorption can be observed for MXene adsorbents.

One of the approaches that was intensely focused is the intercalation of MXene nanochannels via several types of molecules. This approach has an importance of either enhancing the gas permeability due to the increase in interlayer spacing or altering the perm-selectivity of a membrane due to the preferences of intercalated species. Shen et al.^153^ modified MXene by intercalating different molecules such as borate and amine to synthesize CO_2_-selective MXene membranes. The interlayer spacing of MXene was decreased from 15 to 12.9 Å with the temperature increase from 55 to 75 °C, which is related to the enhancement in cross-linking of a MXene surface with the functional groups, and hence CO_2_/CH_4_ selectivity of MXene was manipulated. The reason for this was speculated that with the addition of borate molecules, CO_2_ uptake was increased as 13%, and with the addition of amine, this improvement was raised to 43% (CO_2_ permeance, 350 GPU, and CO_2_/CH_4_ selectivity, 15.3) due to the zipped in-plane slit-like pores [see Figure 2(c–d)]. However, adsorption of H_2_ and CH_4_ showed no change. Additionally, Shen et al.^153^ suggested that pristine MXene membranes were diffusion-selective membranes, which had low H_2_ solubility and high H_2_ diffusivity. However, intercalated MXene membranes with borate and amine were proposed as solution-controlled membranes due to the greater adsorption selectivity (28.3) than diffusion selectivity (0.05). In addition to the intercalation of bulky molecules, ions were also preferred to stem from the strong interaction with the negatively charged MXene nanosheets. Fan et al.^147^ reported superior improvement in binary H_2_/CO_2_ selectivity of MXene intercalated with Ni^2+^ ions (615) compared to the pristine one (215), yielding in almost 3-fold increase. MXene intercalated with Pd^2+^ ions also displayed satisfactory binary H_2_/CO_2_ selectivity as 242,^145^ although not so high as that of MXene intercalated with Ni^2+^ ions.^147^ It was proposed that cationic intercalation is a vital approach to tune interlayer spacing by weakening repulsive electrostatic interactions and promoting a strong interaction between MXene nanosheets, which leads to regular channel size. Lin et al.^159^ aimed to adapt the similar mechanism via intercalating MXene nanosheets with deep eutectic solvent (DES). Rather than observing H_2_-selective membranes as in cation-intercalated membranes, they ended up with a CO_2_-selective membrane with a CO_2_/H_2_ selectivity of 12.4 by the DES intercalation. Collectively, although a limited number and type of intercalants was tested to tune the interlayer distance of MXenes and alter their gas separation properties, the identified ones evidently revealed their efficiency in selectivity due to their size-sieving effect.

To combine the superior permeability and selectivity performances of MXenes with the ease of manufacture of polymers, mixed matrix membranes (MMMs) were addressed where MXenes were used as fillers in a polymeric continuous phase. However, the main issue that was considered is the compatibility between MXene and polymer. Liu et al.^160^ fabricated MMMs consisting of Ti_3_C_2_T_*x*_ and commercial polyether-polyamide block copolymer (Pebax) with different MXene loadings of 0.05, 0.1, 0.15, 0.2, and 0.3 wt %. When MXene loading increased to 0.15 wt %, MMM displayed a uniform structure without aggregation. However, further increase in MXene loading led to the agglomeration of MXene nanosheets and hence the hindrance in the molecular transport through the membrane. Therefore, the greatest CO_2_ permeance and CO_2_/N_2_ selectivity were reported for MMM with MXene loading of 0.15 wt % as ∼22.23 GPU and ∼69.2, respectively. Compared to the Pebax membrane, MMM with MXene loading of 0.15 wt % exhibited around an 81 and 73.4% increase in CO_2_ permeance and CO_2_/N_2_ selectivity, respectively. Similarly, to investigate the interaction between MXene and Pebax and reveal how their assembly affects the separation performance, Shamsabadi et al.^161^ fabricated MMMs with MXene loadings of 0.05, 0.075, 0.1, 0.2, and 0.5 wt %. Considerable enhancement in both CO_2_ permeance and CO_2_/N_2_ selectivity was observed from 987 to 1820 GPU and from 32.1 to 42.0 with the incorporation of MXene loadings of 0.1 wt % into the Pebax polymer, respectively.^161^ They also compared the gas separation properties of MXene- and GO-based MMMs, and MXene-based MMMs were suggested as the optimum membranes for CO_2_ capture due to having enhancement in both separation properties while GO-based MMMs revealed an increase in only selectivity. Additionally, MD simulations were carried out to gain a depth of understanding of the interactions between MXene and Pebax. MD simulations revealed that strong interactions between hard segments of the Pebax and surface functional groups of MXene arose from the hydrogen bonding. This led to the increase in compatibility and homogeneous dispersion of MXene nanosheets in the polymer matrix and, hence, the improvement of CO_2_/N_2_ separation.^161^ Compared to low MXene loadings, Guan et al.^162^ increased it up to 1 wt % and Shi et al.^163^ even further enhanced the MXene content in a Pebax continuous phase up to 20 wt %. Similarly, they demonstrated the use of a certain optimal MXene loading where the permeability reaches a peak value. It was 0.5 wt % for the MMM synthesized by Guan et al.^162^ with CO_2_ permeability of 70.2 Barrer and CO_2_/N_2_ selectivity of 93.2 at 4 bar and 25 °C. Although high MXene loading was handled by Shi et al.,^163^ optimal MXene loading was identified as 1 wt % with CO_2_ permeability of 148 Barrer and CO_2_/N_2_ selectivity of 63 at 2 bar and 30 °C. However, for the humidified MMM, this weight content of MXene in a MMM increased to 10 wt % with a superior performance (584 Barrer CO_2_ permeability and 59 CO_2_/N_2_ selectivity) compared to that of dry MMM.^163^ Considering this limitation in MXene loading, to improve compatibility or in other words to remove the nonselective voids between MXene and polymer phase, the transformation of MXene into nanoscale ionic materials and then fabrication of MMM were proposed. With the surface functionalization of MXene which led to the enhancement in electrostatic interaction with the polymer, Wang et al.^164^ dispersed a great amount of MXene in a polymer phase and ended up with high-performing MMM displaying 176% and 29% increases in CO_2_ and CO_2_/N_2_ selectivity compared to the Pebax membrane, respectively. MXene nanosheets were also dispersed within the poly(ethylene glycol) (PEG) phase^165^ rather than the frequently preferred Pebax one. They revealed greater CO_2_ permeability as 1912 GPU at 1 bar and 25 °C and lower binary CO_2_/N_2_ selectivity as 31.2 than did the Pebax-based MMMs.^165^ As it was experienced from the reported studies including other inorganic fillers in MMMs, there are several approaches to handle the voids between filler and polymer. Therefore, they should be tested for the MXene-based MMMs, rather than just changing its content in polymer phase. Alternatively, there are high technology polymers that can be used as a continuous phase. Since the above-mentioned studies evidently prove the capability of MXene-based MMMs in gas separation, with a proper harmony between the high-performing polymers and MXene nanomaterials, novel MMMs can be designed for the target gas separation application.

In addition to MMMs, nanocomposite membranes were also fabricated with the combination of MXene nanomaterial with other inorganic materials such as MOF. Hong et al.^166^ synthesized a Ti_3_C_2_T_*x*_ (MXene)/ZIF-8(MOF) dual-layered nanocomposite membrane within 21 min with an active membrane layer of 525 cm^2^ using a novel approach. They initially used electrophoretic deposition (EPD) to accumulate MXene nanosheets (thickness of approximately 0.8 μm) onto the copper disc for 1 min and then synthesized a ZIF-8 layer (thickness of approximately 0.45 μm) onto the MXene layer within 20 min using a fast current-driven synthesis (FCDS) approach. Gas permeances of H_2_ and CO_2_ were 379 and 8.4 GPU for pristine MXene membrane, whereas those were 178.2 and 2.3 GPU for the Ti_3_C_2_T_*x*_/ZIF-8 nanocomposite membrane, respectively.^166^ However, the average ideal selectivity of H_2_/CO_2_ for the nanocomposite membrane increased from 44.4 to 77.4 compared to the pristine MXene membrane.^166^ In addition to the alteration of the attractive environment for gas molecules in the nanocomposite membrane compared to the pristine MXene membrane, this performance was attributed to the size change of pores existing for gas transport. Because the interlayer distance of MXene was 3.7 Å and the pore aperture of ZIF-8 was 3.1 Å. In situ synthesis of MOF-801 (pore aperture of 4.7 Å) on the MXene nanosheet was also followed by Li et al.,^167^ leading to the formation of MOF crystals within MXene channels contrary to Hong et al.,^166^ where MOF crystals were grown on the MXene surface. Then, its superiority in H_2_/CO_2_ separation was verified by comparing with a pristine MXene membrane and Ti_3_C_2_T_*x*_/MOF-801 nanocomposite membrane fabricated with a well-known physical intercalation method. H_2_ permeance was improved in both in situ (202%) and intercalated (136%) Ti_3_C_2_T_*x*_/MOF-801 nanocomposite membrane compared to the pristine MXene membrane (773 GPU).^13^ However, due to the local agglomeration of MOF crystals within MXene nanosheets in the nanocomposite membrane fabricated by the intercalation, a severe drop of H_2_/CO_2_ selectivity (5) was observed, contrary to the selectivity improvement (29.4) in the in situ synthesized nanocomposite membrane. Building on this contradictory trend in H_2_ permeance of MXene/MOF nanocomposite membranes fabricated in two different research groups, this might be aroused from the growth of MOF crystals either within MXene channels or on the surface of MXene layer. Further elaboration of this issue is required by testing different MOFs.

Not only are outstanding gas separation properties of membranes essential but also is their long-term stability required for use in industry. Therefore, long-term stability of MXene membranes in gas separation performance was tested by several studies in the order of hours, days, and weeks.^129,144−147,153,156,161,165,166^ Ding et al.^129^ reported that the MXene membrane displayed stable performances with H_2_ and CO_2_ permeability of ∼2210 and ∼15.9 Barrer, respectively, and H_2_/CO_2_ selectivity of ∼147 up to 70 h with shifting between a dry and wet (3 vol % steam) equimolar H_2_/CO_2_ mixture at 25 °C and 1 bar. More importantly, to confirm high temperature durability of MXenes, Fan et al.^144^ applied long-term permeation measurements for H_2_/N_2_ separation at 320 °C and reported stable H_2_ permeance of 612 GPU and the H_2_/N_2_ mixture selectivity of 41 during 200 h. Shen et al.^153^ suggested that as-prepared MXene with a thickness of 0.02 μm and functionalized MXene with borate and poly(ether imide) (PEI) preserved their separation performances of H_2_/CO_2_ and CO_2_/CH_4_ within 100 h at 1.5 bar and 25 °C, respectively. Likewise, MXene/Pebax MMMs with MXene loading of 0.15 wt % were examined for long-term operation in a mixed-gas system at 25 °C and 1 bar by Liu et al.^160^ Effectiveness of MXene nanosheets into the Pebax polymer and the stability of gas separation properties of MMMs were revealed by the stable CO_2_ permeance at 21.6 GPU and CO_2_/N_2_ selectivity at 72.5 up to around 120 h.^160^ Shamsabadi et al.^161^ tested the stability of MXene/Pebax MMMs with a MXene loading of 0.05 wt % at 25 °C and 4 bar for 2 weeks. MMM maintained its CO_2_/N_2_ selectivity of ∼40 up to 15 days, whereas its CO_2_ permeance decreased from ∼1800 GPU to ∼1400 GPU due to the rapid physical aging of highly permeable poly[1-(trimethylsilyl)-1-propyne] (PTMSP) used as a gutter layer. Interestingly, Wang et al.^164^ fabricated the MXene (Ti_3_C_2_T_*x*_) nanoscale ionic materials (NIMs) with a novel synthesis approach benefitted from the electrical interaction, which displayed excellent antioxidation stability after 570 days (stored in air with a tight cap at room temperature), whereas pristine MXene was oxidized within 1 week. Collectively, the stability of MXene membranes under aggressive conditions, high temperature measurement, and long operation times were introduced by several studies.

The other requirement for membranes to be used in industry is to be resistant to the humidity. However, most of the inorganic nanomaterials such as MOFs lose their intensity when they are exposed to the humidity. Therefore, it is a serious challenge in inorganic membranes. Its effect on MXenes’ performance or stability have not been identified properly in the literature, except for a few studies. Li et al.^154^ studied hydrous and anhydrous MXenes to reveal the effect of water intercalation on gas diffusion via molecular simulation methods. Significant alteration in gas diffusion was observed for gas molecules having a large kinetic diameter at a short interlayer distance. For instance, for the interlayer spacing ranging between 6 and 14 Å, the ratios of diffusion coefficients of anhydrous MXene over hydrous MXene varied between 15 and ∼1.5 for H_2_, and 650 and ∼1.7 for CH_4_. This was attributed to the blockage of water molecules for the transport of gases due to dividing transport channels into the small parts resulting in narrow paths for large gas molecules. Furthermore, they displayed that diffusion coefficients of H_2_ and CO_2_ were decreased with a water content increase from 0 to 2.4 wt %. Finally, Li et al.^154^ highlighted that water molecules intercalated within nanosheets enhanced the sieving effect of the MXene membrane, yielding an almost one order of magnitude greater diffusion selectivity than anhydrous MXene. Shen et al.^153^ experimentally studied the humidity effect on gas permeation for ultrathin MXene nanosheets with a 0.02 μm thickness. They reported that H_2_ permeance decreased from 1030 to 618 GPU and CO_2_ permeance increased from 52.3 to 64.3 GPU with a relative humidity increase from 40 to 90%, leading to a decrease in mixed H_2_/CO_2_ selectivity from 19.7 to 9.8. These results were consistent with the simulation data computed by Li et al.^154^ displaying the severe hindrance in H_2_ permeation with the rise in humidity of MXene. However, there is still need for detailed elaboration of the effect of humidity on MXene gas separation performance.

### 2.2 Adsorption-Based Separation

From the discovery of first MXene member, more than 46 different MXene structures^168^ were synthesized for different purposes and almost 730 structures^157^ were theoretically investigated. However, only one of them was dominantly examined for membrane-based gas separation. Unlike this common MXene type, Ti_3_C_2_T_*x*_, there were few studies which investigated the other MXene types^149−151^ experimentally for the adsorption-based gas separation. Liu et al.^149^ fabricated two different MXene types (Ti_3_C_2_T_*x*_ and Ti_2_CT_*x*_) by etching with different salts such as LiF, sodium fluoride (NaF), potassium fluoride (KF), and ammonium fluoride (NH_4_F) in 4 mol/L salt solutions and then intercalated with DMSO, NH_3_·H_2_O (ammonium hydroxide), and urea to investigate the CH_4_ adsorption capacity of MXenes. Highest CH_4_ adsorption capacities were reported as 8.5 cm^3^/g for Ti_3_C_2_T_*x*_ etched with LiF and 11.6 cm^3^/g for Ti_2_CT_*x*_ etched with KF. Since the Li^+^ ion was smaller than the other ions used for etching, it was easily adsorbed and kept on the surface of the MXene, leading to higher CH_4_ adsorption and lower CH_4_ desorption due to the strong interaction between CH_4_ and Li^+^. On the contrary, MXenes exfoliated by NaF and KF released greater amounts of adsorbed methane in the desorption process compared to MXenes exfoliated by LiF, due to the fact that few Na^+^ and K^+^ ions were located on the surface of the MXenes. Liu et al.^148^ examined both theoretically and experimentally the effect of intercalation of NH_3_·H_2_O on the CH_4_ adsorption capacity of MXene (Ti_2_C) under different temperatures. CH_4_ adsorption capacities were measured as 11.58, 18.18, and 52.76 cm^3^ (STP)/g at 25, 40, and 50 °C and 50 bar for as-synthesized MXene, respectively, indicating the positive effect of temperature on adsorption. On the other hand, CH_4_ adsorption improved with the intercalation of NH_3_·H_2_O to 16.81 cm^3^ (STP)/g at 25 °C and 50 bar due to the increase in interlayer spacing. Moreover, theoretical (22.9 wt %) and experimental (28.8 wt %) CH_4_ adsorption capacities agreed well with each other. In addition to CH_4_ adsorption performance, CO_2_ capture of MXene was also identified. Wang et al.^150^ experimentally studied the CO_2_ adsorption performance of Ti_3_C_2_T_*x*_ and V_2_CT_*x*_, which were produced using NaF and HCl as etching solutions at different temperatures. Then, as-synthesized MXenes (as-Ti_3_C_2_T_*x*_ and as-V_2_CT_*x*_) were intercalated with DMSO. This led to the increase in SSA from 21 to 66 m^2^/g for the intercalated Ti_3_C_2_T_*x*_ (int-Ti_3_C_2_T_*x*_) and from 9 to 19 m^2^/g for the intercalated V_2_CT_*x*_ (int-V_2_CT_*x*_). CO_2_ adsorption capacities of Ti_3_C_2_T_*x*_ and V_2_CT_*x*_ were increased from 1.33 to 5.79 mol/kg and from 0.52 to 0.77 mol/kg at 40 bar, respectively, after the intercalation. They emphasized the very exiting conclusion that if Ti_3_C_2_T_*x*_ with theoretical SSA (%100 exfoliated) can be made in the future, the theoretical capacity would be 44.2 mol/kg. On the other hand, they introduced difficulty in the synthesis of V_2_CT_*x*_ and proposed the fabrication of a V_2_CT_*x*_ adsorbent with multilayers rather than a single layer in order to increase its adsorption capacity. However, in a different study, without intercalation, CO_2_ adsorption capacities of Ti_3_C_2_T_*x*_ were measured as 0.603 mol/kg at 10 bar and 25 °C.^169^ Although Petukhov and his co-workers could not reach a high CO_2_ adsorption capacity for Ti_3_C_2_T_*x*_, its superior NH_3_ adsorption performance was revealed as ∼8.2 mol/kg at the same conditions and was related to the occupancy of −(OH)_2_ functional groups on the MXene surface.^169^ By the transformation of Ti_3_C_2_T_*x*_ into nanoscale ionic materials, its CO_2_ adsorption capacities were further improved to 1.80 mol/kg at the same conditions.^164^ Surprisingly, even at severe conditions (0.01 bar and 125 °C) its performance in terms of CO_2_ capture reached to 12 mol/kg. To observe this superior performance, the novel approach was followed to modify the surface termination of MXene as initially treating at high temperature to desorb F atoms, subsequently exposing to H_2_ to remove the persistent O atoms from the surfaces, and then exposing to CO_2_ to synthesize the termination depleted MXene.^170^ Morales-García et al.^151^ theoretically studied MXenes formulated as M_2_C (M = Ti, Zr, Hf, V, Nb, Ta, Cr, Mo, and W) for CO_2_ adsorption. CO_2_ capture increased along the series of MXene following the order of Ti > V > Zr > Nb > Mo > Hf > Ta > W, ranging from 2.34 to 8.25 mol/kg at 25 °C, in accordance with the adsorption bonding energies between CO_2_ and MXene. MXenes exhibited a high CO_2_ adsorption capacity compared to common adsorbents such as zeolites (Ca-X, 13X), GO, and metal oxides. By this exciting study, CO_2_ adsorption capacities of several MXene types have been studied using DFT calculations and their real performances have been revealed for the first time. On the other hand, awesome performance of incompletely etched Ti_2_CT_*x*_ for H_2_ storage was revealed by Liu et al.,^171^ where more than twice the storage performance of previously reported adsorbents under ∼50–60 bar was measured as 8 wt %. Additionally, a positive impact of the narrow interlayer distance and −F_2_ functional groups were introduced on reversible H_2_ storage in Ti_2_CT_*x*_.^171^ It is expected that these pioneer studies will open a new perspective and hopefully accelerate the investigation of synthesis of other MXene types whose gas adsorption performances have not been revealed yet.

In the light of all studies mentioned above, MXene nanomaterials with easily scalable physicochemical properties, unprecedented gas separation capacity, long-term stability, and compatibility with polymeric materials provide an excellent opportunity to develop superior membranes or adsorbents for the gas separation process. For gas separation application, all studies were concentrated on tailoring the interlayer distance of MXene lamellar nanomaterials to obtain zipped in-plane slit-like pores. Especially for the membrane process, to tune the interlayer distance, several strategies were followed with success such as tuning the membrane thickness,^129,146,153−155^ treatment at high temperature,^144,155^ intercalation with ions,^147^ modification with CO_2_ soluble materials like amine^153^ and DES,^159^ and combination with polymeric materials.^160−165^ As a result of such efforts, considerable improvements were achieved, and designed 2D MXene nanomaterials offer new avenues especially for membrane development. However, the most critical issue is the verification of long-term performance of MXene membranes. Instead of testing the pristine MXene membrane, measurements were carried out considering MMMs with a limited period up to a maximum of 8 days. We believe that a longer test period is essential to validate the stability and durability of gas separation MXene membranes. In gas separation applications, the humidity has severe adverse effects on the separation performance of nanomaterials like MOFs. CO_2_ separation improvement with the humidity was revealed with the limited number of studies in MXene membranes.^153,154^ However, further investigation is required for the verification of its effect on performance. Concerning the adsorption process, unlike the general tendency in the membrane process, different types of MXene nanomaterials rather than Ti_3_C_2_T_*x*_ were identified.^149−151^ It is an encouraging manner to experience different members of MXene for gas separation. Collectively, to display the gas separation capacity of the MXene family, Table 1 and Table S1 are tabulated for MXene membranes and adsorbents, which are discussed in this section, respectively. Although the MXene family is still in its initial stage in membrane- and adsorption-based gas separation applications, more efforts should be performed in order to reveal the separation performances of all MXene family via theoretical and experimental studies.

**Table 1:** Survey of Gas Separation Performance of MXene-Based Membranes^a^

membranes	operating conditions	H_2_	CO_2_	N_2_	CH_4_	unit	H_2_/CO_2_	H_2_/N_2_	H_2_/CH_4_	CO_2_/CH_4_	CO_2_/N_2_	ref
Ti_3_C_2_T_*x*_ on AAO (0.005 μm)	1.5 bar, 25 °C	36000	7200			GPU	5					(153)
Ti_3_C_2_T_*x*_ on AAO (0.02 μm)	1.5 bar, 25 °C	1587	51.18	326.8	279.5	GPU	29.19	4.86	5.68	0.183	0.157	(153)
Ti_3_C_2_T_*x*_ on AAO (0.08 μm)	1.5 bar, 25 °C	350	11.29			GPU	31					(153)
Ti_3_C_2_T_*x*_-borate @ 55 °C (0.02 μm)	1.5 bar, 25 °C		436.6		102.3	GPU				4.27		(153)
Ti_3_C_2_T_*x*_-borate @ 75 °C (0.02 μm)	1.5 bar, 25 °C	291.4	322.4	52.9	42.4	GPU	0.9	5.5	6.9	6.1	7.6	(153)
Ti_3_C_2_T_*x*_-borate @ 95 °C (0.02 μm)	1.5 bar, 25 °C		91.19		17.88	GPU				5.10		(153)
Ti_3_C_2_T_*x*_-borate-PEI @ 75 °C (0.02 μm)	1.5 bar, 25 °C	246.7	349.5	28.9	22.8	GPU	0.7	8.5	10.8	15.3	12.1	(153)
Ti_3_C_2_T_*x*_ on AAO (0.8 μm)	1 bar, 25 °C	1209		54.9	110.4	GPU		22	11			(144)
Ti_3_C_2_T_*x*_ on AAO (0.8 μm)	1 bar, 320 °C	890		21.8	149.3	GPU		41	5.96			(144)
Ti_3_C_2_T_*x*_ on AAO (0.2 μm)	1 bar, 25 °C	2176.43	850.57	883.25	766.51	Barrer	2.56	2.46	2.84			(129)
Ti_3_C_2_T_*x*_ on AAO (2 μm)	1 bar, 25 °C	2402.3	10.1	18.6	3.08	Barrer	238	129	780			(129)
Ti_3_C_2_T_*x*_ on AAO (5.1 μm)	1 bar, 25 °C	1796.43	7.12	8.35	2.22	Barrer	252.30	215.14	809.20			(129)
Ti_3_C_2_T_*x*_ on AAO (0.02 μm)	1 bar, 22 °C	4090		201.5		GPU		20.3				(156)
Ti_3_C_2_T_*x*_ on AAO (0.8 μm)	1 bar, 22 °C	698.5		51.9		GPU		13.4				(156)
Ti_3_C_2_T_*x*_ on AAO (1 μm)	1 bar, 22 °C	477.6		24.2		GPU		19.8				(156)
Ti_3_C_2_T_*x*_ (15 wt %)/Pebax on PAN (2 μm)	2 bar, 25 °C		22.23	0.321		GPU					69.2	(160)
Ti_3_C_2_T_*x*_ (0.05 wt %)/Pebax on PVDF (60 μm)	4 bar, 25 °C	325.6	1986.5	47.5	134.2	GPU				14.8	41.8	(161)
Ti_3_C_2_T_*x*_ (0.075 wt %)/Pebax on PVDF (60 μm)	4 bar, 25 °C	313.9	1915.0	46.7	129.4	GPU				14.8	41.0	(161)
Ti_3_C_2_T_*x*_ (0.1 wt %)/Pebax on PVDF (60 μm)	4 bar, 25 °C	312.1	1810.3	43.2	120.7	GPU				15.0	42.0	(161)
Ti_3_C_2_T_*x*_-Ni^2+^ inter. on Al_2_O_3_ hollow fiber (2.7 μm)	1 bar, 25 °C	321	0.53	0.72	1.55	GPU	750	430	240			(147)
Ti_3_C_2_T_*x*_-Pb^2+^ inter. on AAO (0.78 μm)	1 bar, 25 °C	794				GPU	242					(145)
Ti_3_C_2_T_*x*_ (0.5 wt %)/Pebax (50 μm)	4 bar, 25 °C	5	70.24	0.92	2.5	Barrer				28	93.18	(162)
Ti_3_C_2_T_*x*_ on PC (2 μm)	0.6 bar, 25 °C	894.86	223.81	303.46	422.94	GPU				0.1058	0.083	(159)
Ti_3_C_2_T_*x*_-DES inter. on PC (3 μm)	0.6 bar, 25 °C	2.128	26.35	0.083	0.106	GPU				249.01	319.15	(159)
Ti_3_C_2_T_*x*_ (1 wt %)/Pebax (dry) (0.001–0.002 μm)	2 bar, 30 °C		148	2.35		Barrer					63	(163)
Ti_3_C_2_T_*x*_ (10 wt %)/Pebax (humidified) (0.001–0.002 μm)	2 bar, 30 °C		584	9.9		Barrer					59	(163)
Ti_3_C_2_T_*x*_ @ 140 °C on YSZ hollow fiber (0.05 μm)	1 bar, 25 °C	6193	2670			GPU	2.32					(146)
Ti_3_C_2_T_*x*_ @ 140 °C on YSZ hollow fiber (0.22 μm)	1 bar, 25 °C	70.6	2.33			GPU	30.3					(146)
Ti_3_C_2_T_*x*_ (25 wt %)/PEG (400) (0.5 μm)	1 bar, 25 °C		1543			GPU				25.39	30.9	(165)
Ti_3_C_2_T_*x*_ (25 wt %)/PEG (600) (0.5 μm)	1 bar, 25 °C		1627	51	58.38	GPU				27.87	32.18	(165)
Ti_3_C_2_T_*x*_-NIM (60 wt %)/Pebax (80–130 μm)	1 bar, 25 °C		91.9	1.58		Barrer					58.2	(164)
Ti_3_C_2_T_*x*_ (1 wt %)/CTA (∼50 μm)	1.5 bar, 25 °C		7		0.21	Barrer				34		(192)
Ti_3_C_2_T_*x*_ (3 wt %)/CTA (∼50 μm)	1.5 bar, 25 °C		16		0.28	Barrer				57.14		(192)
Ti_3_C_2_T_*x*_ on PES (0.67 μm)	1 bar, 25 °C	379	8.4			GPU	44.4					(166)
Ti_3_C_2_T_*x*_/ZIF-8 on PES (0.45 μm)	1 bar, 25 °C	178.2	2.3			GPU	77.4					(166)
Ti_3_C_2_T_*x*_ on PTFE	1 bar, 25 °C	773	45.5			GPU	17					(167)
Ti_3_C_2_T_*x*_/MOF-801 (intercalated) on PTFE	1 bar, 25 °C	1824	358			GPU	5.1					(167)
Ti_3_C_2_T_*x*_/MOF-801 (in situ growth) on PTFE	1 bar, 25 °C	2334	79.4			GPU	29.4					(167)

a Membrane thickness is given in parentheses. AAO: anodic aluminum oxide, DES: deep eutectic solvent, NIM: nanoscale ionic materials, PAN: polyacrylonitrile, PC: polycarbonate, Pebax: polyether-polyamide block copolymer, PEG: poly(ethylene glycol), PES: polyether sulfone, PTFE: poly(tetrafluoroethylene), PVDF: polyvinylidene fluoride, and YSZ: yttria-stabilized zirconia.

Rather than gas separation and gas capture research, a great number of studies are also concentrated on gas sensing performance of MXenes. Selective gas and vapor sensing of MXenes were studied either experimentally or theoretically in the literature where some examples are as follows: Ti_3_C_2_T_*x*_^172^ for NH_3_, SO_2_, H_2_S, NO, and CO_2_; V_2_CT_*x*_^173^ for C_2_H_5_OH, C_3_H_6_O, NH_3_, CH_4_, H_2_, and H_2_S; thin film Ti_3_C_2_T_*x*_^174^ for N_2_, CO_2_, and C_2_H_5_OH; Ti_3_C_2_T_*x*_^175,176^ and Ti_3_C_2_T_*x*_/polyaniline^177^ for C_2_H_5_OH, CH_3_OH, C_3_H_6_O, and NH_3_; Ti_2_C,^178,179^ V_2_C,^179^ Nb_2_C,^179^ and Mo_2_C^179^ for NH_3_, H_2_, CH_4_, CO, CO_2_, N_2_, NO_2_, and O_2_; V_3_C_2_^180^ and Nb_3_C_2_^180^ for N_2_; cation-intercalated Ti_3_C_2_T_*x*_^181^ for NH_3_; Sc_2_CO_2_^182^ for SO_2_; tyrosinase-Ti_3_C_2_/chitosan^183^ for C_6_H_6_O; Ti_3_C_2_T_*x*_ with Au electrodes^184^ and CTAB-delaminated Nb_2_CT_*x*_^185^ for C_2_H_5_OH, C_3_H_6_O, NH_3_, C_3_H_8_O, NO_2_, SO_2_, and CO_2_; 3D Ti_3_C_2_T_*x*_ framework^186^ for volatile organic compounds (VOCs); Ti_3_C_2_T_*x*_/W_18_O_49_^187^ for ppb-level detection of C_3_H_6_O; and Ti_3_C_2_T_*x*_/Fe_2_(MoO_4_)_3_^188^ for low working temperature detection. Remarkable sensing performances of MXene nanomaterials for several gas types were ascribed to its high surface area, unique structure, and good conductivity. It is not the topic of this review; for detailed information, please refer to these comprehensive reviews.^189−191^

## 3 Solvent Dehydration

In many different industries, organic synthesis is carried out in a solvent environment to produce high value-added products. Therefore, recovery and recycle of high amounts of organic solvents remaining at the end of the chemical reaction are required for the economic development and environmental protection. Both the recovery of a high-value solute from a dilute solution (solute enrichment) and the solvent by removing an impurity dissolved in it (solvent recovery) are achieved successfully by organic solvent nanofiltration (OSN) membranes. In this section, only solvent recovery performance of MXene OSN membranes will be discussed in terms of solvent permeance. On the other hand, water-solvent mixtures (aqueous solutions) were used in some chemical reactions to reduce the cost and save the environment rather than using pure solvents. To separate this mixture, pervaporation membranes were fabricated with a purpose of selectively removing water in a vapor phase.

**Figure 3:**
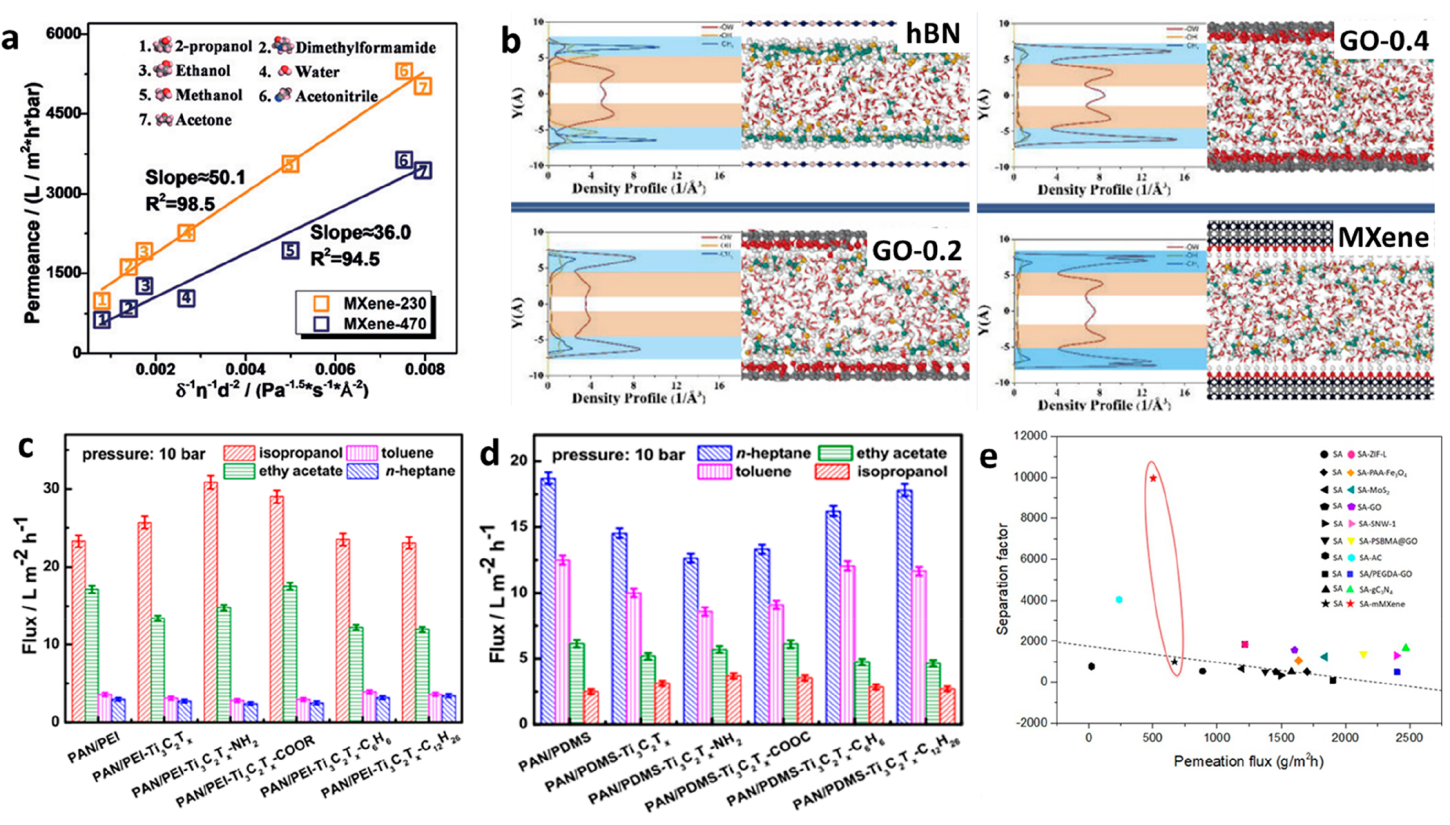
Solvent dehydration performance of MXene membranes. (a) Solvent permeances against the combined solvent property for MXene membranes with two different thicknesses. Adapted with permission from ref (128). Copyright 2018 Wiley Online Library. (b) (Left) Density profiles of methyl (blue line) and hydroxyl groups (yellow line) in ethanol molecules, and oxygen atoms (red line) in water molecules within the hBN, GO-0.2, GO-0.4, and Ti_3_C_2_(OH)_2_. Adapted with permission from ref (195). Copyright 2020 Elsevier. The light blue and orange regions denote the interfacial contact and the subcontact layers, respectively. (Right) The snapshots show the front view of the slits. The solvent fluxes of (c) PEI-based and (d) PDMS-based membranes under 10 bar. Adapted with permission from ref (204). Copyright 2017 Elsevier. (e) Detailed comparison of MXene/SA MMMs with various reported 2D nanomaterial-based membranes for ethanol dehydration performance. Adapted with permission from ref (200). Copyright 2020 Elsevier.

Nowadays, OSN membranes are mostly dominated by organic polymer materials. As an alternative, inorganic membranes were proposed due to their large surface area, high stability in many conditions, and great hydrophilic properties. Recently, MXenes are counted in these inorganic membranes due to their superior hydrophilicity and ultrafast molecular transport. Unique solvent separation performance of MXene membranes was first provided by Wang et al.^128^ They produced Ti_3_C_2_T_*x*_ and GO membranes with varying membrane thicknesses to demonstrate the usage of rigid, regularly stacked nanosheets for the solvent separation. Water permeances of MXene with 0.23 μm and GO with 0.21 μm in solvated (rehydrated) state were reported as 2302 (1703) and 257 (129) L/m^2^ × h × bar, respectively.^128^ In addition to these exceptional water permeances of MXene membranes, unique solvent permeances, which were much greater than the highest reported values in the literature, were also observed for MXenes with two different membrane thicknesses. Solvent permeances of MXene were in the order of acetonitrile-acetone (5022.4) > methanol (3563) > ethanol (1916) > dimethylformamide (1616) > 2-propanol (983 L/m^2^ × h × bar) as given in Figure 3(a).^128^ Similarly, Kang et al.^193^ reported that the permeances of MXene with the thickness of 0.09 μm were 25, 6.62, 3.17, 2.14, and 0.79 L/m^2^ × h × bar at 5 bar for water, hexane, toluene, c-hexane, and isopropanol (IPA), respectively, indicating superior selectivity of MXene for water-IPA mixture due to the existing hydrogen bonding between IPA and Ti_3_C_2_T_*x*_. Although the solvents have different sizes, these two preliminary studies revealed the importance of the interaction between MXene and solvents in solvent separation. Since it has been demonstrated that MXene membranes display superior solvent permeances compared to the well-known 2D inorganic membranes such as GO and graphene, Wei et al.^194^ targeted to fabricate a composite lamellar membrane of Ti_3_C_2_T_*x*_ and GO with various MXene loadings of 0, 50, 60, 70, 80, and 100 wt % in order to optimize the solvent separation performance of inorganic membranes. The solvent flux of the nanocomposite membrane increased with MXene loadings. Nanocomposite membranes with MXene loading of 70 wt % displayed nearly ten times higher acetone flux as 48.32 L/m^2^ × h than GO membranes, followed by the methanol, ethanol, and IPA fluxes of 25.03, 10.76, and 6.18 L/m^2^ × h, respectively. Qin et al.^195^ evaluated the separation performances of MXene, hexagonal boron nitride (hBN), and GO for the water–ethanol mixture by identifying the microstructure of solvent confinement within the nanochannels via MD simulations. They suggested that while ethanol molecules were observed as an accumulated layer near the solid surfaces of all 2D membranes, water molecules exhibited balanced distribution within the slits, as represented in Figure 3(b). Diffusion coefficients of water molecules for Ti_3_C_2_(OH)_2_, hBN, and GO in the subcontact layer were reported as 0.85, 0.81, and 0.60 × 10^–9^ m^2^/s, respectively.^195^ Since the average numbers of hydrogen bond (HB) per water molecules between the interfacial and sub contact layers for Ti_3_C_2_(OH)_2_ (∼2.5) was greater than both hBN and GO (<0.8), Ti_3_C_2_(OH)_2_ was suggested as the best membrane within the investigated membranes for solvent separation.^195^ Hereby, it has been evidently demonstrated that MXene membranes have a promising potential for solvent separation (see Tables S2 and S3).

To enhance the solvent-specific separation performance, different strategies were developed for the MXene membranes used in pervaporation and OSN processes. Liu et al.^196^ embedded macromolecules into MXene nanosheets to investigate its solvent dehydration performance via the pervaporation process. They introduced hyper-branched polyethyleneimine (HPEI) into the Ti_2_CT_*x*_ nanosheets and then carried out interfacial polymerization. Its water fluxes for methanol, ethanol, and IPA dehydration systems were reported around 2240, 1430, and 1020 g/m^2^ × h with water contents of 12.6, 82.4, and 99.2 wt %, respectively.^196^ The comparison of separation properties between pristine and intercalated Ti_2_CT_*x*_ membranes for IPA dehydration provided that while the water content of the intercalated Ti_2_CT_*x*_ membrane was significantly greater than that of the pristine one (11 wt %), the water flux of the pristine Ti_2_CT_*x*_ membrane (6378 g/m^2^ × h) surpassed that of intercalated Ti_2_CT_*x*_, proving the insertion of HPEI molecules resulted in more stacked, regular membranes without defects.^196^ Liu et al.^197^ in their further study examined the effect of modification of Ti_2_CT_*x*_ with different loadings of positively charged polyelectrolyte, polydiallyl dimethylammonium chloride (PDDA), on dehydration of IPA. Their aim in modification of MXene was to build electrostatic interaction between MXene nanosheets and macromolecules and consequently improve the water affinity of membranes by manipulating the water flux. With the increasing PDDA concentration and MXene loading amounts, the total flux decreased because of the enhanced membrane thickness, and the separation factor first increased and then decreased because of the aggregation of PDDA and improper distribution of MXene.^197^ Optimum separation performance with the total flux of 1237 g/m^2^ × h and separation factor of 1932 belonged to the MXene/PDDA membrane with the PDDA loading of 0.2 mg/mL and MXene amount of 288 mg/m^2^.^197^ Accordingly, while embedment of macromolecules within the nanochannels increased the flux, the separation performance of the 2D inorganic membrane was hindered. Therefore, the studies on gaining improvement in separation performances were escalated. Wu et al.^198^ studied the pristine MXene membrane having various thicknesses from 0.5 to 2.0 μm to separate an ethanol–water mixture via a pervaporation process. The separation factor increased from ∼20 to ∼83, and the total flux decreased from ∼592 to ∼221 g/m^2^ × h for 90% ethanol aqueous solution with membrane thickness, indicating the prolonged mass transfer path with increased mass transfer resistance.

MXene-based MMMs were synthesized with several organic polymers to investigate the improved solvent dehydration performance in the pervaporation process. Xu et al.^199^ fabricated Ti_3_C_2_T_*x*_/chitosan (CS) MMMs with different MXene loadings of 0, 1, 3, and 5 wt % and evaluated the separation of three typical azeotropic mixtures: water-ethanol, water-ethyl acetate, or water-dimethyl carbonate. The total flux and ethanol separation factor of MMMs increased with MXene loading up to 3 wt % at 50 °C from ∼1150 to 1424 g/m^2^ × h and from 407 to 1421, respectively. For 98 wt % water-ethyl acetate and water-dimethyl carbonate mixtures, solvent separation factors of MMM with a MXene amount of 3 wt % were totally different, reaching to 4898 and 906 at 50 °C, respectively. Li et al.^200^ investigated the pervaporation performance of Ti_3_C_2_T_*x*_/sodium alginate (SA) MMMs with MXene amounts of 0, 0.06, 0.12, 0.18, and 0.24 wt % for ethanol dehydration. Lower optimum MXene loading was observed for the system of Ti_3_C_2_T_*x*_/SA than Ti_3_C_2_T_*x*_/CS as 0.12 wt % with a separation factor of 9946 and total flux of 514 g/m^2^ × h.^200^ Since the hydrophilicity of membrane increased with MXene content, the interaction of water molecules with a membrane matrix enhanced through strong hydrogen bonds, leading to an increase in the adsorption of water and confinement of ethanol molecules. Compared to the other modified SA membranes for pervaporation performance, MXene/SA MMMs exhibited the highest separation factor [see Figure 3(e)] but comparable water flux, probably due to the uniform distribution of MXene sheets within the matrix and excellent compatibility as a result of cross-linking. Similarly, Cai et al.^201^ also studied the effect of MXene amounts of 0, 0.5, 1, 2, 3, and 4 wt % for ethanol dehydration in Ti_3_C_2_T_*x*_/poly(vinyl alcohol) (PVA) MMMs. As the amount of MXene increased from 0 to 3.0 wt %, total flux decreased from 97 to 75 g/m^2^ × h and the separation factor increased from 144 to 2585, again suggesting an optimum MXene content in MMMs which was related to the improved cross-linking density. A greater separation factor was reported by Yang et al.^202^ for Ti_3_C_2_T_*x*_/PVA MMM cross-linked with sulfosuccinic acid (SFA). MMM including 20 wt % SFA and 2 wt % MXene revealed water content in the permeate as 97.6, 99.5, 99.7, and 99.9 wt % with separation factors of 968, 4738, 7913, and 23,786 for methanol, ethanol, isopropanol, and tert-butanol aqueous solutions, respectively. However, compared to the uncross-linked one,^201^ ethanol dehydration performance was diminished, displaying a water flux of 1489 g/m^2^ × h and a separation factor of 4738.^202^ These studies paved a new way toward fabricating MMMs in order to combine favorable properties of MXene and the polymeric phase, thereby enhancing flux without sacrificing separation factor for solvent dehydration. Although the solvent separation performance of MXene is promising for pervaporation membranes, more studies are required for the complete understanding.

Similarly, in the OSN process, to improve the separation performance of MXene membranes, either surface functionalization to design the interlayer spacing of MXene or fabrication of composite membranes by incorporating MXene nanomaterials into the polymeric phase were performed. The experts from Zhengzhou University published successive papers related to these strategies. Initially, thin film nanocomposite (TFN) membranes were fabricated using hydrophilic PEI or hydrophobic polydimethylsiloxane (PDMS) polymers and Ti_3_C_2_T_*x*_ having abundant −(OH)_2_ groups at various weight ratios. Then, their solvent fluxes [ethanol, isopropanol, butanone (polar) and ethyl acetate, toluene, *n*-heptane (nonpolar)] were examined.^203^ With the increase of MXene content in both hydrophilic and hydrophobic composite membranes, flux for nonpolar solvents was reduced, whereas it was initially increased and then either decreased or kept stable for polar solvents. For instance, the greatest isopropanol flux for PEI-based TFN membrane was observed at 2 wt % MXene loading as 33.5 L/m^2^ × h at 10 bar.^203^ In their following study, they fabricated TFN membranes using the same type of polymers and functionalized Ti_3_C_2_T_*x*_ with −NH_2_, −COOR, −C_6_H_6_, and −C_12_H_26_ groups.^204^ For both PEI- and PDMS-based TFN membranes, −NH_2_ and −COOR functionalization led to improvement in polar and decrease in nonpolar solvents compared to unfunctionalized TFN membranes [Figure 3(c–d)]. Then, the same group in another study^205^ proposed a novel approach for the membrane fabrication and investigated separation of solvent mixtures. Heterostructured membranes were fabricated using hydrophilic pristine Ti_3_C_2_T_*x*_ and PEI as well as hydrophobic Ti_3_C_2_T_*x*_ functionalized with a −C_6_H_5_ group and PDMS via initial vacuum filtration of MXene onto a support and then coating its surface with polymer. They utilized from this novel membrane formation by selectively capturing polar solvents from the mixture via PEI and then introducing them into hydrophilic MXene nanochannels. This leads to the fast transport of polar solvents and hindered movement of nonpolar solvents, resulting in selective separation of the solvent mixture. Specifically, they reported a toluene separation factor of ∼4.46, 3.31, 2.34, and 2.0 for Ti_3_C_2_T_*x*_/PEI membranes from acetonitrile, methanol, acetone, and ethyl acetate solutions, respectively.^205^ However, those for Ti_3_C_2_T_*x*_/PDMS membrane functionalized with the −C_6_H_5_ group were 3.41, 4.68, 2.39, and 1.12, respectively.^205^ Finally, they investigated free-standing MXene membranes functionalized with −NH_2_, −C_6_H_5_, and −C_12_H_25_ groups to identify the effect of only functionalization on solvent flux.^206^ They reported that functionalization with either hydrophilic or hydrophobic groups did not influence the transport of nonpolar solvents, whereas hydrophobic functionalization resulted in the considerable decrease in the flux of polar solvents compared to pristine and hydrophilic functionalized MXene. Using molecular simulation approaches, they proposed that nonpolar solvents interacted weakly with the MXene surface compared to polar solvents and, hence, randomly orientated within the MXene channels, leading to almost similar permeation performance. However, polar solvents displayed ordered molecular alignment especially within hydrophilic MXene nanochannels, resulting in fast transport. Collectively, these studies evidently prove that functional groups attached to the surface of MXene nanosheets are capable of solvent-specific separation with a high solvent flux.

Besides, to overcome trade-off between flux and separation factor in solvent dehydration or solvent mixture separation, the other most important point is their durability/stability/swelling for long-term operation even under harsh conditions. Wang et al.^128^ proved the excellent permeance of pristine MXene with the thickness of 0.23 μm for water and isopropanol, which were decreased only 4 and 11%, respectively, for 25 h. Likewise, Wu et al.^198^ examined the pristine MXene membrane with a thickness of 2 μm for ethanol dehydration at room temperature and observed a stable total flux during 48 h. Accordingly, it can be deduced that the thickness of MXene does not affect the membrane stability for solvent dehydration. However, composite membranes with different polymers led to the changes in flux. For instance, for the HPEI intercalated Ti_2_CT_*x*_ membrane^196^ in IPA dehydration, flux decrement was reported around 18% for the 120 h of continuous operation at 50 °C. However, for the PPDA-intercalated membrane^197^ the flux decline was around 8% within 120 h at 50 °C. On the other hand, solvent flux along 12 h under 10 bar for Ti_3_C_2_T_*x*_–NH_2_/PEI (IPA) and Ti_3_C_2_T_*x*_–C_12_H_26_/PDMS (*n*-heptane) composite membranes resulted in 29.3 and 31.0% drops, respectively.^204^ This situation is more promising in MMMs where stable total flux of Ti_3_C_2_T_*x*_/CS^199^ for ethyl acetate dehydration was observed within 30 h and Ti_3_C_2_T_*x*_/PVA^201^ preserved its flux and separation factor for ethanol dehydration about 7 days. Because of their superior flux stability for long-term operations, MXene membranes emerged as promising inorganic materials in solvent separation processes.

Concerning the OSN and pervaporation MXene membranes applied for solvent dehydration, it successfully benefited from the regularly stacked form of MXene nanosheets. For this separation application, primarily importance was claimed to be the interactions between MXene and the solvents. Therefore, to tailor these interactions, two main attitudes were followed such as intercalation of bulky and charged molecules within the MXene nanochannels and production of composite or mixed matrix membranes via combining MXene with different polymers. The former enables us to benefit from the electrostatic interaction between them via a charged surface and to tune the interlayer distance specific to the solvent molecule via its molecular weight. However, the highly studied strategy is the latter one, which utilizes the polymer’s performance. Although pristine MXene membranes were preferred due to enabling the fabrication in a form of few-nanometer nanosheets and hence the reduction in the mass transfer resistance, flux decrements were recorded for them. However, MMMs or composite membranes have an outstanding potential in practical use of solvent separation, as a result of their high stability in long-term operation even under harsh conditions. Therefore, it is not surprising to focus on MXene-based MMMs or composite membranes on solvent separation.

## 4 Dye Removal

**Figure 4:**
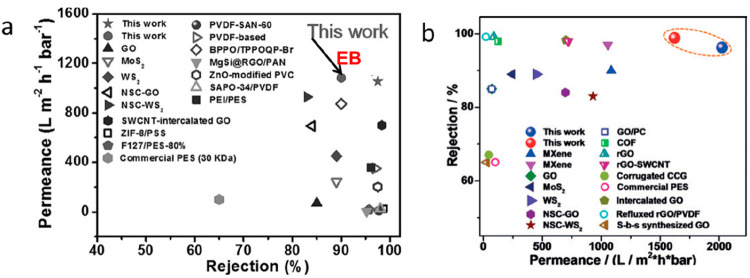
Performance comparison between MXene membranes and various previously reported membranes for various dyes. (a) EB separation data measured by Ding et al. Adapted with permission from ref (127). Copyright 2017 Wiley Online Library. (b) AY79 separation data measured by Wang et al. Adapted with permission from ref (128). Copyright 2018 Wiley Online Library.

Considering that more than several tons of dyes are produced annually by textile, paint, and pigment industry and nearly 10–15% of dyes are released to the water, dyes are the major pollutants for water and the environment. Thereby, removal of dyes from wastewater safely and effectively is the vital importance for a sustainable green ecosystem. The removal methods of dyes from water are the physical, chemical, and biological methods such as coagulation, flocculation, membrane filtration, adsorption, ion-exchange, oxidation, electrochemical process, photocatalysis, and biodegradation. Among these processes, membrane and adsorption technologies are the most demanding processes due to their high efficiency and operation simplicity. Inorganic nanomaterials have gained value in the application of these two technologies with their strength, high surface area, and low mass. Since MXenes possess a layered structure providing large and highly accessible surfaces,^207,208^ the use in dye removal as an inorganic membrane and adsorbent material emerged, and their current performances are tabulated in Table S4 and Table 2, respectively. Within this context, to benefit from short transport pathways and large amounts of nanochannels of MXenes in order to enhance water transport, Ding et al.^127^ fabricated MXene (Ti_3_C_2_T_*x*_) membranes by intercalating Fe(OH)_3_ nanoparticles. They investigated the rejection of several dyes such as rhodamine B (RhB), 5,10,15,20-tetrakis(n-methyl-4-pyridyl)-21,23-h-porphyrin tetratosylate (TMPyP), and Evans Blue (EB). The MXene membrane prepared by embedding Fe(OH)_3_ at the initial step and then removing Fe(OH)_3_ exhibited extremely high water permeance, 1084 L/m^2^ × h × bar and moderate EB rejection rate, 90% due to the enlargement of interspace between nanosheets by the insertion of Fe(OH)_3_. Similar dye separation performance was also observed for RhB (805 L/m^2^ × h × bar, 85%) and TMPyP (921 L/m^2^ × h × bar, 93%).^127^ The observed superior dye separation performance for MXene membranes compared to the existing nanofiltration membranes [see Figure 4(a)] encouraged the membrane community and advanced membrane technology. On the other hand, before the application of MXene as a membrane material, it was used as an adsorbent to remove dyes from wastewater. For the first time, Mashtalir et al.^209^ fabricated multilayered MXene (Ti_3_C_2_T_*x*_) and used it as an adsorbent to reveal its dye adsorption and degradation performance. They tested methylene blue (MB) (0.05 mg/mL) and acid blue (AB) (0.06 mg/mL) as cationic and anionic dyes, respectively, under dark and UV light. Under UV light, 62 and 81% decreases in concentration were observed for AB and MB, respectively. However, the decrease was only 18% for MB in the dark environment, indicating that UV light promoted the degradation of dyes significantly. Therefore, Mashtalir et al.^209^ highlighted the photocatalytic property of MXene and showed its high adsorption efficiency for cationic dye, MB. After these preliminary studies, MXene nanomaterials have drawn considerable attention as both membrane and adsorbent materials for dye removal.

**Table 2:** Survey of Dye Removal Performance of MXene Adsorbents^a^

adsorbent	*d*-spacing/interlayer spacing (Å)	surface area (m^2^/g)	test conditions (P: atm, T: °C, pH)	adsorbent dosage (mg)	dye	initial dye concentration (mg/L)	adsorption capacity (mg/g)	ref
Ti_3_C_2_T_*x*_	9.3	—	1, 25, 7	10	MB	50	21	(247)
Ti_3_C_2_T_*x*_ (functionalized with −SO_3_H)	14.3	—	1, 25, 7	10	MB	50	111	(247)
Ti_3_C_2_T_*x*_ (DMSO intercalated and hydrated)	20.18	—	1, 25, 5	12	MB	100	125	(254)
Ti_3_C_2_T_*x*_ (hydrated)	7.52	—	1, 25, 5	12	MB	100	78	(254)
Ti_3_C_2_T_*x*_ (dry)	1.52	—	1, 25, 5	12	MB	100	7.8	(254)
Ti_3_C_2_T_*x*_	—	9	1, 20, 9	25	MB	10	140	(252)
Ti_3_C_2_T_*x*_/PhA (hydrothermal treatment with 12 h)	—	—	1, 25, 7	10	MB	12	42.5	(242)
Ti_3_C_2_T_*x*_/PhA (hydrothermal treatment with 12 h)	—	—	1, 25, 7	10	RhB	6	22.8	(242)
Ti_3_C_2_T_*x*_ (stirring-assisted)	—	—	1, 20, 7	20	MB	5	85	(245)
Ti_3_C_2_T_*x*_ (UV-assisted with 28 kHz)	—	—	1, 20, 7	20	MB	5	130	(245)
Ti_3_C_2_T_*x*_ (UV-assisted with 580 kHz)	—	—	1, 20, 7	20	MB	5	110	(245)
Ti_3_C_2_T_*x*_ (functionalized with −COOH)	—	—	1, 25, —	10	MB	10	39.4	(246)
Ti_3_C_2_T_*x*_ (functionalized with −COOH)	—	—	1, 25, —	10	NR	20	20.2	(246)
Ti_3_C_2_T_*x*_ (functionalized with −COOH)	—	—	1, 25, —	10	ST	30	31.6	(246)
Ti_3_C_2_T_*x*_-COOH (treated with (PEI/PAA)_10_	—	—	1, 25, —	10	MB	10	40.4	(246)
Ti_3_C_2_T_*x*_-COOH (treated with (PEI/PAA)_10_)	—	—	1, 25, —	10	NR	20	46.1	(246)
Ti_3_C_2_T_*x*_-COOH (treated with (PEI/PAA)_10_)	—	—	1, 25, —	10	ST	30	35.6	(246)
Ti_3_C_2_T_*x*_ (treated with terephthalate)	—	135.7	1, 20, 7	10	MB	100	209.5	(248)
Ti_3_C_2_T_*x*_	20.44	—	1, 25, 7	100	MB	50	99.9	(249)
Ti_3_C_2_T_*x*_ (treated with NaOH)	26.2	—	1, 25, 7	100	MB	50	184.2	(249)
Ti_3_C_2_T_*x*_ (treated with LiOH)	26.4	—	1, 25, 7	100	MB	50	118.9	(249)
Ti_3_C_2_T_*x*_ (treated with KOH)	24.92	—	1, 25, 7	100	MB	50	74.2	(249)
Ti_3_C_2_T_*x*_ (decorated with Fe_3_O_4_)	—	—	1, 25, 7	25	MB	40	2.1	(250)
Ti_3_C_2_T_*x*_ (decorated with Fe_3_O_4_)	—	—	1, 40, 7	25	MB	40	4.2	(250)
Ti_3_C_2_T_*x*_ (decorated with Fe_3_O_4_)	—	—	1, 55, 7	25	MB	40	11.7	(250)
Ti_3_C_2_T_*x*_ (traditional etching)^b^	—	8.9	—	100	MB	50	8.5	(258)
Ti_3_C_2_T_*x*_ (hydrothermal etching)^b^	—	44.6	—	100	MB	50	12.5	(258)
Ti_3_C_2_T_*x*_/PEI (modified with SA)	—	16.31	1, 25, 3	10	CR	150	1300	(262)
Ti_3_C_2_T_*x*_	—	—	1, 25, 4	10	MB	5	121.6	(263)
Ti_3_C_2_T_*x*_(grafted with polyelectrolyte (AMPS-co-AA))	—	—	1, 25, 4	10	MB	5	68.03	(263)
Ti_3_C_2_T_*x*_ (grafted with polyelectrolyte (DAMPS-co-AA))	—	—	1, 25, 4	10	MB	5	67.88	(263)
Ti_3_C_2_T_*x*_	—	—	1, 25, 2	40	MB	6.4	64.3	(264)
Ti_3_C_2_T_*x*_ (modified with SA (30%))	—	12.0	1, 25, 7	40	MB	100	92.0	(259)
Ti_3_C_2_T_*x*_ (functionalized with peroxo)	—	—	1, 25, 5.6	25	MB	200	558.0	(253)
Ti_3_C_2_T_*x*_ (functionalized with peroxo)	—	—	1, 25, 5.6	25	RhB	200	524.6	(253)
Ti_3_C_2_T_*x*_ (functionalized with peroxo)	—	—	1, 25, 5.6	25	CR	100	258.2	(253)
Ti_3_C_2_T_*x*_ (functionalized with peroxo)	—	—	1, 25, 5.6	25	MO	100	292.6	(253)
Ti_3_C_2_T_*x*_	—	—	1, 25, 2	90	MG	10	4.8	(265)
Ti_3_C_2_T_*x*_/PDA (functionalized with cellulose)	—	38.43	1, 25, 7	50	MB	100	112.45	(266)
Ti_2_CT_*x*_	12.8	18.6	1, 35, 6	35	MB	300	544.1	(266)
Ti_3_C_2_T_*x*_	15.97	—	1, 25, 8	4	MR	50	61.56	(267)
Ti_3_C_2_T_*x*_	15.38	—	1, 25, 8	4	MO	50	12.29	(267)
Ti_3_C_2_T_*x*_	14.72	—	1, 25, 8	4	OG	50	1.15	(267)
Ti_3_C_2_T_*x*_	—	12.45	1, 25, 7	—	CR	90	20	(253)
Ti_3_C_2_T_*x*_	—	12.45	1, 25, 7	—	MB	90	60	(253)
Ti_3_C_2_T_*x*_ (alkalized with acrylic acid)	—	95.51	1, 25, 7	—	CR	90	265	(253)
Ti_3_C_2_T_*x*_ (alkalized with acrylic acid)	—	95.51	1, 25, 7	—	MB	90	195	(253)
Ti_3_C_2_T_*x*_/Co_3_O_4_	—	—	—, 25, —	10	RhB	5	47.1	(256)
Ti_3_C_2_T_*x*_/Co_3_O_4_	—	—	—, 25, —	10	MB	12.5	128.9	(256)
Ti_3_C_2_T_*x*_	—	4.71	1, 25, 7	20	MB	200	105	(255)
Ti_3_C_2_T_*x*_	—	4.71	1, 25, 7	20	RhB	200	58	(255)
Ti_3_C_2_T_*x*_/Fe_3_O_4_	—	8.77	1, 25, 7	20	MB	200	153	(255)
Ti_3_C_2_T_*x*_/Fe_3_O_4_	—	8.77	1, 25, 7	20	RhB	200	86	(255)
Ti_3_C_2_T_*x*_	10.01	—	1, 25, —	15	MB	—	9.0	(257)
Ti_3_C_2_T_*x*_/ZIF-8	13.43	—	1, 25, —	15	MB	—	107	(257)
V_2_CT_*x*_	7.9	26.6	1, 25, 11	15	MB	20	111.11	(260)
Nb_2_CT_*x*_ (traditional etching)^b^	—	—	—	100	MB	50	3.5	(258)
Nb_2_CT_*x*_ (hydrothermal etching)^b^	—	—	—	100	MB	50	6.5	(258)
Nb_2_CT_*x*_	9.7	44.69	1, 25, 7	100	MO	500	493	(261)
Nb_2_CT_*x*_	9.7	44.69	1, 25, 7	100	MB	500	496	(261)
Ti_3_C_2_T_*x*_	—	—	1, 30, 6	20	MB	100	63.07	(268)
Ti_3_C_2_T_*x*_ (modified with EHL (50%))	—	—	1, 30, 6	20	MB	100	102.5	(268)

a AA: acrylic acid, Alk: alkalized, AMPS: 2-acrylamido-2-methylpropane sulfonic acid, DMAPS: (2-(methacryloyloxy) ethyl] dimethyl-(3-sulfopropyl) ammonium hydroxide, DMSO: dimethyl sulfoxide, EHL: enzymatic hydrolysis lignin, PhA: phytic acid, PAA: poly(acrylic acid), PEI: polyethylene polyimide, SA: sodium alginate. CR: Congo Red, MB: methylene blue, MG: malachite green, MO: methyl orange, MR: methyl red, NR: neutral red, OG: orange G, RhB: rhodamine B, and ST: safranine T.

b Calculated based on initial dye concentration.

### 4.1 Membrane-Based Separation

As it was clarified in the previous section, OSN membranes are used for both solvent recovery and separation of high-value solutes such as dyes, pharmaceuticals, etc. from solvents and/or water. However, for simplicity, generally in lab scale tests, solute separation from water rather than solvents is tested for OSN membranes. Especially, since the MXene family is a new type of nanomaterial, the first trials for dye separation were carried using aqueous solutions. To define dye separation performance of membranes, mainly, free-standing MXene membranes were easily fabricated by a vacuum filtration method on various polymeric membrane supports such as polyethersulfone (PES),^210^ mixed cellulose ester (MCE),^211,212^ polyvinylidene difluoride (PVDF),^213^ nylon 66,^214,215^ etc. Compared to the first study performed by Ding et al.^127^ where free-standing MXene membrane fabricated by initially embedding and subsequently removing Fe(OH)_3_ nanoparticles, there are few studies which have found almost the same performances for MXene membranes. Wang et al.^128^ reported water permeances of Ti_3_C_2_T_*x*_ for the solvated state and the state of dried and subsequently hydrated as 2302 and 1703 L/m^2^ × h × bar with acid yellow 79 (AY79) rejection rates of 96.3 and 98.9%, respectively [see Figure 4(b)]. For different MXene type, Hu et al.^216^ prepared Nb_2_CT_*x*_ MXene membranes composed with SA under vacuum-filtration and measured their separation performance for various dyes such as basic blue (BB), rhodamine 6G (Rh6G), and toluidine. Water fluxes of Rh6G, BB, and toluidine were reported as 2164, 2001, and 2209 L/m^2^ × h × bar, respectively, with ∼100% rejection rates for each dye.^216^ However, following studies where pure MXene membranes were synthesized revealed almost one order of magnitude lower water permeance with almost the same dye rejection rates for various dyes.^210,211^ Han et al.^210^ reported rejection rates of 92.3 and 80.3% for congo red (CR) and gentian violet (GV) with a water flux of 115 L/m^2^ × h at 1 bar, respectively. Similarly, Zhang et al.^211^ proposed the same order of water transport property as 120 and 84 L/m^2^ × h for pure water and MB aqueous solution, respectively. While water permeance decreased to 45 L/m^2^ × h, rejection rate increased to 100% with the increase in MXene loading at 1 bar.^211^ Then, to improve water permeance, the intercalation of nanoparticles within MXene nanochannels was examined. For instance, Pandey et al.^213^ used silver nanoparticles to prepare nanocomposite membranes by varying the silver content (0, 7, 14, 21, 28, and 35 wt %). While pristine MXene membrane displayed comparably lower water fluxes as 90 and 85 L/m^2^ × h × bar for solutions including RhB and methyl green (MG), respectively, the nanocomposite membrane with 21 wt % Ag exhibited water fluxes of 387 and 354 L/m^2^ × h × bar without sacrificing the dye rejection rate. Alternatively, He et al.^214^ synthesized nanocomposite membranes via the intercalation of various amounts of UiO-66, which is a new class of zirconium-based porous MOF, into MXene nanosheets. For different MB concentrations, flux of the nanocomposite membrane including 1.5 mg of UiO-66 was reported around 750 L/m^2^ × h × bar with the rejection rate greater than 99.2%. However, in the presence of various oils such as *n*-hexane, isooctane, 1,3,5-trimethyltoluene, and toluene in emulsions, its fluxes decreased to about 443, 488, 328, and 446 L/m^2^ × h × bar, respectively, without the alteration in dye rejection rates. Long et al.^215^ inserted Al_2_O_3_ nanoparticles in a different amount within the channels of Ti_3_C_2_T_*x*_. Excellent improvement (%408) in water permeability of nanocomposite membrane at a 1/1 mass ratio of MXene/Al_2_O_3_ was observed increasing from ∼21 to 86 L/m^2^ × h × bar with a MB rejection rate of 99%. Spectacular design was proposed by Tao et al.^217^ via the intercalation of carboxymethyl-β-cyclodextrin (CM-β-CD) into Ti_3_C_2_T_*x*_ channels. Considerable enhancement in total permeance of aqueous MB solution was achieved for Ti_3_C_2_T_*x*_/CM-β-CD membrane compared to the pristine MXene from 18.5 up to 431 L/m^2^ × h × bar (23-fold) with the rejection rate greater than 99% for five different dyes.^217^ These studies evidently display the importance of intercalation of specific nanoparticles into MXene channels to modulate the interlayer distance and, hence, the flux. Although the performance of membranes fabricated via the intercalation of some nanoparticles within the MXene nanochannels could not exceed those of membranes proposed by Ding et al.,^127^ it was suggested that they surpassed the water permeance of some commercial and novel nanocomposite membranes.^211,213^

To further improve separation performance of dye as well as alter the interlayer spacing of MXenes, surface functionalization was applied. Wu et al.^206^ modified the Ti_3_C_2_T_*x*_ surface with hydrophilic (−NH_2_) and hydrophobic groups (−C_6_H_5_, −C_12_H_25_). Compared to the various dye rejection rates of hydrophilic and hydrophobic MXenes, Ti_3_C_2_T_*x*_-NH_2_ displayed higher rejection rates. Acid–base functionalization of the MXene surface is the other strategy to tailor the interlayer distance.^218,219^ Yi et al.^218^ modified Ti_3_C_2_T_*x*_ with a various number of organic phosphonic acid (OPA) groups. As expected, with the increase in the number of OPA in the functional group, *d*-spacing of MXene was improved from 12.2 to 16.5 Å. For the MXene functionalized with bulky OPA groups, water fluxes in aqueous solutions including CR (*M*_W_: 696.66 g/mol) and eriochrome black T (BT) (*M*_W_: 461.38 g/mol) were reported as 514.5 and 508.6 L/m^2^ × h × bar with the rejection rates of 99.6 and 98.3%, respectively.^218^ Functionalization with bulky groups were also carried out by the study of Yousaf et al.,^212^ where they reported the removal efficiency of several dyes in the range of 52–98% for membranes coupled with three different silane agents. Tong et al.^219^ was aimed not only to alter the interlayer distance by functionalizing with acid–base groups (tannic acid) but also to synthesize the Ti_3_C_2_T_*x*_ membrane having dye-selective ability by modifying acid groups with multivalent ion salts such as FeCl_3_, CuCl_2_, and ZnCl_2_. Regardless of the type of ion salt, all surface modified MXene membranes exhibited high CR rejection ratios which were above 92% and similar water permeability (∼260 L/m^2^ × h × bar).^219^ However, the MXene membrane modified with different multivalent ion salts displayed the best rejection performance for different dye types. Rather than the effect of molecular weight of dye molecules, it was attributed to their charges, where positively charged one was attracted and negatively charged one was repelled from the membrane surface, arising from the negative surface charge of MXene. As highlighted with these preliminary studies, the effect of functionalization of the MXene surface for dye separation is still more complex, where several factors should be considered.

To further enhance the water flux of MXene membranes, instead of fabrication of free-standing membranes, composite membranes via different polymers were proposed. For instance, Pandey et al.^220^ prepared chemically cross-linked composite membranes consisting of cellulose acetate (CA) and MXene via a phase inversion method. Among the various MXene contents, membranes including 10 wt % MXene over CA revealed pure water flux of 256 L/m^2^ × h × bar with RhB and MG rejections of 92 and 98%, respectively. Alternatively, the composite membrane prepared by the encapsulation of MXene via silk fibroin displayed greater dye rejection reaching to 99% and water permeance of 324.7 L/m^2^ × h × bar.^221^ Additionally, Han et al.^222^ synthesized chemically cross-linked MMMs using copolyimide (P84) as a continuous phase and MXene as a filler. Total fluxes of MMM containing 1 wt % of MXene for GV and CR were 268 and 380 L/m^2^ × h × bar, respectively, with a 100% rejection rate for GV and 78.5% for CR. Alternative to the use of commercial and highly preferred polymer type in the fabrication of MMMs, polydopamine (PDA)^223^ and poly(ionic liquid)^224^ polymers were also tested combining with MXene as a filler for the separation of several dyes. Due to the synergy between these novel polymers and MXene, which leads to the enhancement in hydrophilicity of the membrane, water flux improved almost twice. Interestingly, poly(ionic liquid)s including counteranions was proposed as a hydrophilicity modifier, which alters the water transport and rejects dyes selectively via a precise selection of the counterion.^224^ An intriguing approach was also suggested by the group of Wu,^225^ who fabricated MMM using PES and Ti_3_C_2_T_*x*_/ZIF-8 nanocomposite synthesized via a facile one-pot microemulsion strategy. However, the dye separation performance of MMM with 3% microemulsion content was in the order of other MMMs with only MXene. Rather than mixing MXene with a polymer matrix, the final strategy is to intercalate MXene into nanofibers. For instance, Li et al.^226^ reinforced the Kevlar nanofibers with Ti_3_C_2_T_*x*_ and observed improved rejection performance for several dyes. The average molecular weight cutoff (MWCO) for the composite membrane was reported as in between 600 and 800 Da considering the analysis based on the different molecular weight of PEG molecules.^226^ Compared with the free-standing membranes where only the pure MXene was fabricated on a polymeric support,^210,211^ MXene/polymer composite membranes^220−224^ offered almost twice the water flux with stable dye rejection performance.

On the other hand, it is worth mentioning that although MXene/polymer composite membranes^220,222^ provided a lower water flux compared to free-standing nanocomposite membranes (Ti_3_C_2_T_*x*_/Ag^213^ and Ti_3_C_2_T_*x*_/UiO-66^214^), they are free from leaching of the MXene layer as a result of weak binding of MXene to the support layer. Therefore, they are comparably more mechanically stable and have constant water transport properties during several operation times. This topic will be discussed in the following paragraphs.

Alternative to silver nanoparticle and porous inorganic nanomaterials such as MOFs that were used to fabricate MXene-based nanocomposite membranes, among the pool of nanomaterials, GO has been remarkably used to prepare a nanocomposite membrane with MXene for the removal of dyes from water. The main reason for this preference was its excellent properties such as large surface area (2600 m^2^/g), small interlayer spacing, varied functional groups, high mechanical strength, and plain structure. Considering excellent properties of MXene and GO, the design of nanocomposite membranes by combining them has become an alternative strategy in order to obtain high separation performance for dyes. Kang et al.^193^ prepared a free-standing Ti_3_C_2_T_*x*_/GO nanocomposite membrane including 30 wt % of GO on a polycarbonate (PC) support to demonstrate its separation potential for methyl red (MR), MB, rose bengal (RB), and brilliant blue (BB). Total permeances of Ti_3_C_2_T_*x*_/GO nanocomposite membranes were 2.1, 0.3, 0.67, and 0.23 L/m^2^ × h × bar at 5 bar with the rejection rates of 68, 99.5, 93.5, and 100% for MR, MB, RB, and BB solutions, respectively. It was suggested that dye molecules with a hydrated radii larger than 5 Å (MB: 5.04 Å, RB: 5.88 Å, BB: 7.98 Å) displayed lower permeation with higher rejection rates due to the hindrance of transport through nanochannels. Wei et al.^194^ tested dye separation performance of several Ti_3_C_2_T_*x*_/GO nanocomposite membranes prepared by varying MXene loadings as 0, 50, 60, 70, 80, and 100 wt % for five different dye types. With the alteration of the ratio of MXene over GO, fluxes of the nanocomposite membrane for both pure water and aqueous dye solution reversely changed with dye rejection, indicating the requirement of a well-thought-out design for the nanocomposite membrane. Ti_3_C_2_T_*x*_/GO nanocomposite membrane with the MXene amount of 70 wt % exhibited the optimum performance for the removal of different dyes with the rejection rates greater than 90% and total flux ranging between 18 and 24 L/m^2^ × h × bar. Similar behavior was also supported by the study of Liu et al.^227^ where the separation performances of Ti_3_C_2_T_*x*_/GO nanocomposite membranes for five dyes, which were different than those used in the study of Wei et al.,^194^ were investigated. Similarly, the change of the MXene/GO ratio inversely effected total flux and dye rejection. As a consequent, the optimum performance was observed for the nanocomposite membrane having a MXene/GO ratio of 4/1 as an average total flux of 71.9 L/m^2^ × h × bar and dye rejection rates greater than 99.5%. A different strategy was applied to improve dye separation performances of Ti_3_C_2_T_*x*_/GO nanocomposite membranes by Han et al.^228^ They fabricated nanocomposite membranes with different MXene loadings and then applied H_2_O_2_ to convert MXene into TiO_2_ nanocrystals via in situ oxidation. However, separation properties of the nanocomposite membrane with optimum performance were reported in the order of the results obtained by Liu et al.^227^ who did not apply any of the oxidation process. To enhance the adhesion between MXene and GO layers, as it was applied for other nanocomposite membranes, Feng et al.^229^ and Zeng et al.^230^ used dopamine as a cross-linking agent. Compared to the previously reported total flux values for Ti_3_C_2_T_*x*_/GO nanocomposite membranes having optimum performances, higher fluxes were reported as 174, 90, 89, 116, and 109 L/m^2^ × h × bar for MB, MR, MO (methyl orange), CR, and EB dye solutions with similar dye rejection performances.^229^ Collectively, by varying MXene and GO content simultaneously, optimal balance was caught for the MXene/GO nanocomposite membrane. The main reason for the better separation properties of MXene/GO nanocomposite membranes compared to pure MXene and pure GO membranes was related among the above studies to the nonselective regions existing in MXene and a critical role of GO in ensuring the selective filtration of MXene. Alternative to GO, as a carbon-based material, carbon nanotubes (CNTs) were proposed to fabricate nanocomposite membranes with MXene due to the possible size-sieving ability of CNTs.^231,232^ Ding et al.^231^ reported excellent water permeance of 1270 L/m^2^ × h × bar for the Ti_3_C_2_T_*x*_/CNT nanocomposite membrane and related it to the unique fusiform structures between CNT bundles and MXene nanochannels, leading to the fast water transport. This structural organization was not surprising and observed also with the insertion of COF^233^ and Bi_2_S_3_^234^ nanoparticles in MXene nanochannels. However, Sun et al.^232^ displayed that with thermal cross-linking of CNT and MXene nanomaterials, interlayer spacing within the membrane decreased, yielding in the shrinkage of channels and drop in water flux, which was in the order of the performance of MXene/GO nanocomposite membranes.

In addition to pressure-assisted permeation of water and dye separation in pristine MXene nanofiltration membranes, electric field-assisted permeation was also studied via the application of negative and positive voltages. It was also suggested that on top of the size-sieving ability of MXene membranes, their voltage-gated sieving ability could be used for dye separation benefiting from the electrostatic interactions between dyes and MXene, which are regulated by changing the applied external voltage.^235,236^ Therefore, it reveals that the separation performance of MXene membranes can be enhanced via mean contribution of different driving forces.

**Figure 5:**
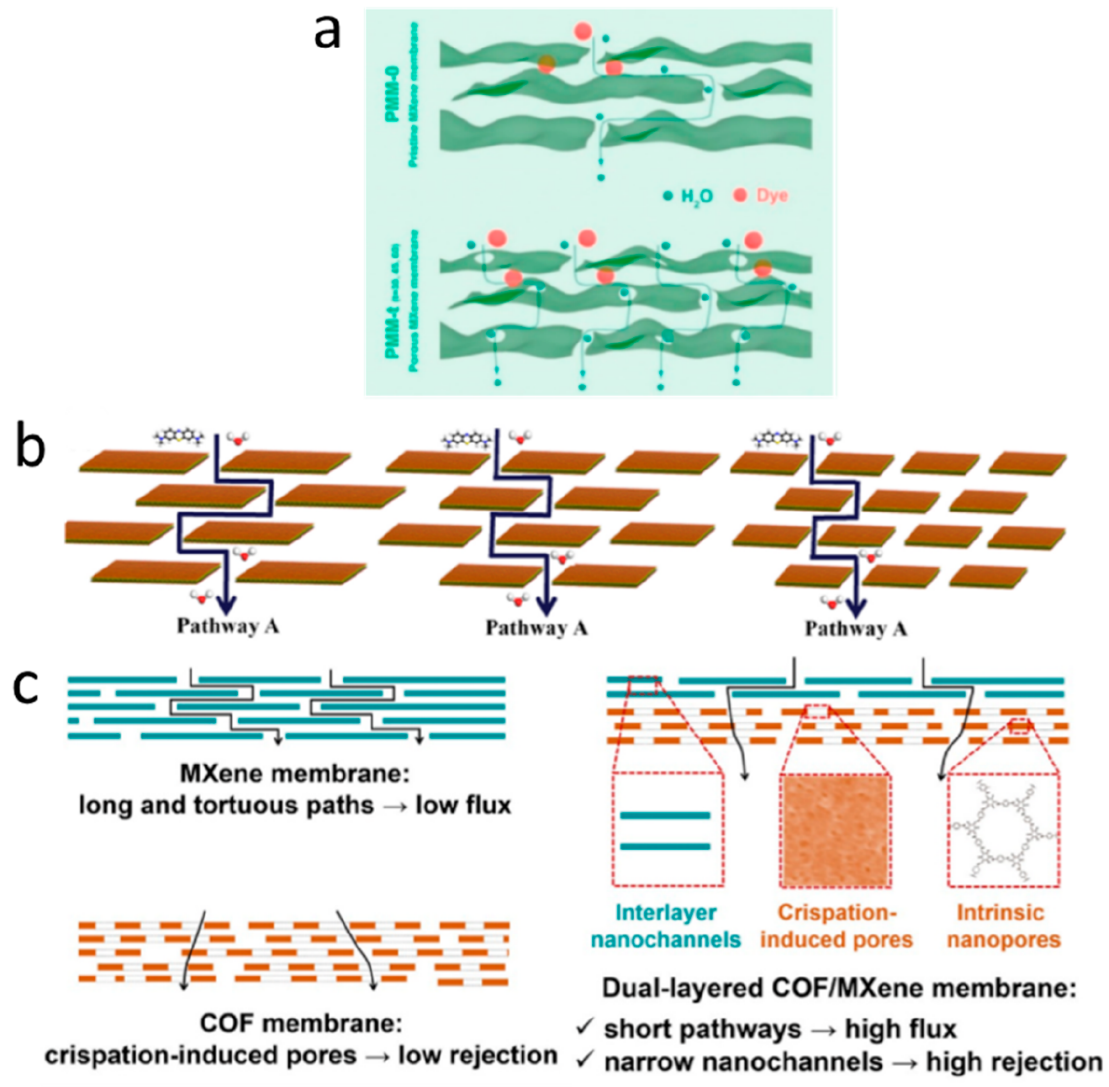
Schematic illustration of the possible pathways for water in (a) porous MXene, Adapted with permission from ref (239). Copyright 2021 Elsevier, (b) MXene having different lateral sizes, Adapted with permission from ref (240). Copyright 2022 Elsevier, and (c) MXene having different lateral size combined with porous COF. Adapted with permission from ref (241). Copyright 2022 Elsevier.

According to the above-mentioned studies, the dye separation performances of MXene membranes are accelerated by the modification of either the surface or interlayer distance of MXene via intercalation, functionalization, or composite and nanocomposite membrane fabrication. However, some basic analysis such as structural or methodological analysis in the synthesis of MXene should also be examined in detail, probably to enhance membrane performance. Although there are some studies revealing the effect of synthesis method, lateral size, or structural deformations of MXene on its dye separation performance, to gain more understanding, different aspects should be evaluated. For instance, Kim et al.^237^ fabricated pristine MXene membranes via both vacuum-filtration and slot-die coating methods. They reported three-times greater pure water permeance as 190 L/m^2^ × h × bar with the dye rejection rate greater than 90% for several dyes for the membrane fabricated by the slot-die coating method, compared to the one synthesized with the highly preferred and simple vacuum filtration method (66 L/m^2^ × h × bar and 77.9–97.9%).^237^ On the other hand, regarding the structural properties, Xing et al.^238^ identified that without following any modification methods discussed above, just fabrication crumbled MXene membranes improved the dye separation capacity of MXene compared to the flat MXene membranes. Crumbled MXene membranes fabricated via the freeze-drying method displayed a two orders of magnitude greater solvent (water, acetone, methanol, ethanol, 1-propanol, and *n*-butanol) permeation and with a little compromise from the dye rejection at high MXene loading.^238^ A similar observation was proposed by the study of Li et al.^239^ where pores or, in other words, new pathways [see Figure 5(a)] for the transport of water were introduced via the chemical etching of MXene with hydrogen peroxide (H_2_O_2_). Xiang et al.^240^ examined the effect of lateral-size of MXene nanosheets on dye separation. Since the MXene membrane having small lateral size offered a short pathway [see Figure 5(b)] for water transport, they reported better water permeance and comparable dye rejection compared to the MXene having large lateral size. Feng et al.^241^ combined these two strategies [Figure 5(c)] by introducing porous material as COF and fabricating a MXene/COF membrane top to each nanomaterial having different lateral sizes. Collectively, handling the pathway of molecules for their transport within MXene membranes is an efficient strategy to improve dye removal capacity of MXene membranes without following any modification approaches.

**Figure 6:**
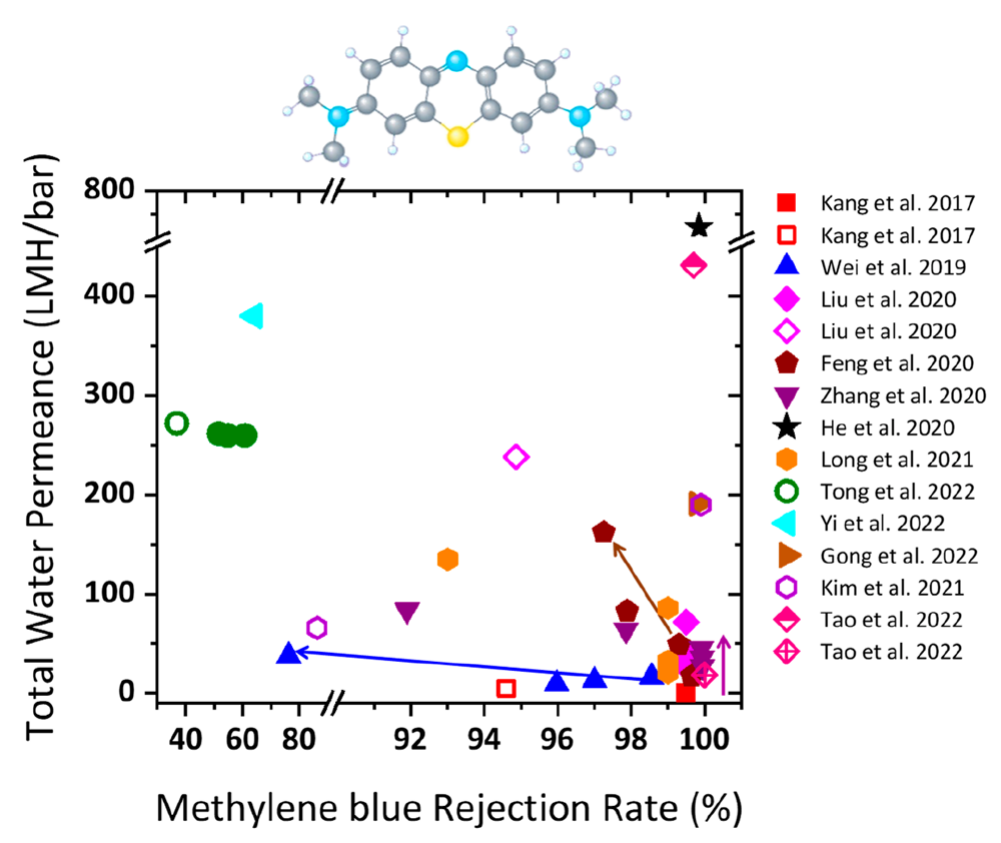
Comparison of methylene blue separation performance of pristine MXene (empty symbols) and the MXene nanocomposite (full/partially full symbols) membranes measured by several groups (Kang et al.,^193^ Wei et al.,^194^ Liu et al.,^227^ Feng et al.,^229^ Zhang et al.,^211^ Long et al.,^215^ Tong et al.,^219^ Yi et al.,^224^ Gong et al.,^233^ Kim et al.,^237^ He et al.,^214^ and Tao et al.^217^). Arrows represent the alteration in MXene content in a nanocomposite membrane. LMH: L/m^2^ × h.

MB (3,7-bis(dimethylamino)-phenothiazin-5-iumchloride) is a cationic dye widely used for biological staining, dyeing of paper, wool, cotton, tannin, clothes, and for the treatment of methemoglobinemia and urinary tract infections. However, above a certain concentration causes carcinogenicity and health problems such as breathing, vomiting, eye burns, etc. Therefore, it is necessary to develop effective strategies for the separation of MB from wastewater. Several studies have been carried out to remove MB effectively using MXene OSN membranes.^193,194,211,214,215,227,242^ Since we aimed to display the limits of MXene membranes for each separation application, we plot MB separation performances of several MXene membranes to draw a frame for a specific particle in Figure 6. In accordance with the collected data from the literature, a big portion of data dominated mainly on the region of rejection rate greater than 99%. This evidently reveals the applicability of MXene for the dye separation from wastewater, despite the varying of total water permeances of MXene membranes in the vicinity of 50 L/m^2^ × h × bar. Even so, fortunately, thanks to unique MXene modification approaches, there are some promising MXene membranes having the water permeance in the order of hundreds with greater MB rejection than 95%.

Not only is superior dye separation performance of MXene-based membranes necessary but also is their long-term stability vital for the long-term use of membranes. Since long-term stability is directly related to the applicability of membranes in industrial processes, several studies concentrated on this issue for MXene membranes.^211,220,222,229,237^ Feng et al.^229^ reported a 17% decrease in total flux of the Ti_3_C_2_T_*x*_/GO nanocomposite membrane cross-linked with dopamine after six cycles with a stable MB rejection rate and flux recovery rate greater than 97.7% at each cycle. Similarly, Pandey et al.^220^ revealed the excellent flux recovery rate of 98% for thin film membranes prepared with MXene and CA during third cycle filtration of RhB solution with only a 3.5% flux decline. Stability in water permeance and slight drop in MB rejection of Ti_3_C_2_T_*x*_ intercalated with cucurbit[5]uril were reported considering great number of filtration cycles, as 30.^243^ Long-term stability of MMM fabricated by Han et al.^222^ was investigated under harsh conditions and comparably long operation times. MMMs immersed in different solvents and then GV separation performances were measured for 18 days. Dye rejection rates of each membrane soaked in each solvent kept stable. However, approximate flux decline ranging between 50 and 60% was observed after immersion.^222^ It was attributed to the membrane fouling by dyes, which garners attention to the importance of fouling behavior of membranes. Rather than composite or nanocomposite MXene membranes, pristine MXene membrane fabricated via the slot-die-coated method displayed high stability for 30 days without extra post-treatment,^237^ although it is well-known that MXene can be easily oxidized in aqueous solution to form TiO_2_.

One of the major limitations, which restrains the membrane applicability is the fouling described as the filtration performance decline over time. The pores of a membrane are blocked by particles and these particles start to accumulate on the surface during nanofiltration. To overcome this challenge, Pandey et al.^213^ used a different content of silver nanoparticles (0, 7, 14, 21, 28, and 35 wt %) and produced nanocomposite membranes. Flux recovery rates of MXene loaded with 21 wt % of Ag and pristine MXene were reported as 97 and 86% for MG, respectively. This superior performance was ascribed to the ability of Ag to improve the resistance of membrane towards organic foulants via its hydrophilic nature. Most importantly, this nanocomposite membrane exhibited high antimicrobial activity with a bacteria growth inhibition as >99%, whereas that of pristine MXene was ∼60% which is still high and that cannot be underestimated. This was the first study that indicated the importance of designing antifouling and antibiofouling membrane concurrently. In another study of the same group,^220^ as we mentioned above, they revealed excellent flux decline resistance to RhB with the composite membrane fabricated via the phase inversion method using MXene and CA. Additionally, they proposed that with the increase of MXene content, growth inhibition for two different bacteria (*E. coli* and *B. subtilis*) increased and reached to 98 and 96% for the membrane having a 10 wt % of MXene content, revealing a high antibiofouling performance of MXene. Flux recovery ratio is the other parameter used to define the fouling behavior of a membrane. Kallem et al.^244^ reported a considerably high flux recovery rate of the solution including humic acid (HA), SA, and BSA (94–97%) for the membrane fabricated with the grafting MXene via zwitterions and then mixing with PES. However, these values ranged around 60% for the pristine PES membrane and 80% for the unmodified MXene/PES nanocomposite membrane.^244^ Mainly, the modification to repel the adsorption of foulants on the membrane surface via electrostatic repulsion forces was suggested in the literature.^213,244^ With these exciting studies, the requirement to produce an antifouling membrane has been underlined significantly again.

### 4.2 Adsorption-Based Separation

An alternative to membrane technology, MXene nanomaterials were also used as adsorbents to remove dye from wastewater. The adsorption process is a highly efficient separation technique with its initial cost, simplicity of design, and ease of operation. Since the main target is to compose an adsorbent with high adsorption performance for dyes, MXene nanomaterials were aimed to design with a large interlayer spacing in order to use their full adsorption capacity by either functionalization via various groups^242,245−248^ or physical treatment to intercalate various particles.^249−251^ Jun et al.^252^ compared the removal performance of unmodified commercial MXene with Al-based (A100) MOF for dyes of MB (cationic) and AB (anionic). Although MXene and MOF had different surface areas as 9 and 630 m^2^/g, respectively, they revealed opposite removal rates for each dye. More specifically, removal rate of MB was achieved as 80% (140 mg/g) and 40% by MXene and MOF, respectively. On the other hand, the removal rate of AB was above 80% (200 mg/g) for MOF, whereas no AB was removed by MXene. Therefore, selective separation can be achieved with MXene adsorbents. To design efficient MXene as an adsorbent for the removal of dyes, the widely preferred strategy in the literature was to functionalize the MXene surface via either short or bulky groups. Jun et al.^245^ performed chemical modification by using ultrasonication to observe oxygenated functional groups on the surface of MXene and evaluated its adsorption performance for MB and MO removal. Compared to the pristine MXene (90 mg/g at pH 7 and 20 °C), MB adsorption capacities of MXene treated with two different frequencies (28 kHz and 580 kHz) were increased as 30.8 and 18.2%. Similarly, to increase the oxygenated functional groups on the surface of MXene, Li et al.^253^ observed peroxo-functionalized Ti_3_C_2_T_*x*_ with the exposure of H_2_O_2_, which revealed excellent dye adsorption performances of 558.0, 524.6, 292.6, and 258.2 mg/g for MB, RhB, MO, and CR, respectively. On the other hand, Cai et al.^242^ observed greater enhancement with the functionalization of MXene using phytic acid (PhA) by the hydrothermal process under different operation times (0.5, 3, 6, 12, and 30 h). Although pristine MXene had a very low adsorption performance for MB and RhB, PA-functionalized MXene for 12 h revealed 54 and an 80% improvement in dye adsorption performance and reached to 42.51 and 22.79 mg/g, respectively, at 25 °C and pH 7. The same strategy was followed by the study of Lei et al.^247^ where arenediazonium salts were used to intercalate and functionalize MXene via −SO_3_H groups. Since *d*-spacing of MXene increased from 9.3 to 14.3 Å after the intercalation of aromatic compounds within nanosheets, MB adsorption capacity of functionalized MXene enhanced 81% from 21 to 111 mg/g. It can be deduced that the attachment of comparably similar size of functional groups has led to almost the same adsorption capacity improvement regardless of dye type and initial performance of MXene. This information was also verified by Li et al.^246^ who modified MXene by −COOH groups and more bulky groups as polyethylene polyimide and then poly(acrylic acid) (PAA) using a layer-by-layer assembly method. While −COOH modified MXene revealed similar improvement as 81 and 89% in the adsorption capacity for MB and safranine T (ST), respectively, direct functionalization with a bulkier group did not lead to further significant enhancement. However, Hao et al.^253^ revealed that before functionalization with acrylic acid, treatment with an alkaline solution suggested a tremendous enhancement in dye adsorption performance as 3- and 13-times greater than pristine MXene for MB and CR dyes, respectively. Collectively, it can be suggested that to improve the surface adsorption capacity of MXene, functional groups should be interpenetrated through MXene channels causing the enlargement of interlayer spacing between nanosheets and serving as favorable adsorption sites. Therefore, a moderate size of functional groups is more efficient compared to a bulky one. Additionally, the stability of this enlarged interlayer spacing during the adsorption process was the other concern. Therefore, Vakili et al.^248^ proposed a pillaring approach to cross-link two MXene surfaces using terephthalate and reported excellent increase in the surface area from 5.4 to 135.7 m^2^/g and MB uptake of 209.5 mg/g.

The second way proposed to increase dye adsorption capacity of MXene in the literature was the intercalation of MXene via several molecules or nanoparticles by physical treatment. Wei et al.^249^ treated MXene (Ti_3_C_2_T_*x*_) with different alkaline solutions including LiOH, NaOH, and KOH to benefit from the ionic radius of alkaline ions for the enlargement of interlayer spacing. MB adsorption capacities of NaOH, LiOH, and KOH treated and pristine MXene were reported as 189, 121, 77, and 100 mg/g, yielding change of 47, 17, and −30%, respectively.^249^ Alternatively, Wang et al.^254^ treated Ti_3_C_2_T_*x*_ with DMSO and NaOH solutions. Specifically, after DMSO treatment, an extreme increase in MB uptake was observed from 8 to 125 mg/g. Several researchers^250,251,255^ synthesized the MXene (Ti_3_C_2_T_*x*_)/Fe_3_O_4_ nanocomposite material using Fe_3_O_4_ nanoparticles via the in situ growth method and investigated for MB adsorption performance. Zhu et al.^250^ measured the maximum MB removal performance of nanocomposite adsorbent as 11.68 mg/g at 55 °C while 2.10 mg/g at 25 °C. The same performance was also observed by the nanocomposite adsorbent fabricated by Zhang et al.^251^ as 3.8 and 11.68 mg/g at 25 and 55 °C, respectively. More importantly, Zhang et al.^251^ investigated the dye removal from binary dye solutions including separately MO and RhB along with MB. Removal efficiencies of MXene/Fe_3_O_4_ for MB from both binary solutions were 94%, whereas those for MO and RhB were reported as 17% and 5%, respectively, proving the selective adsorption performance of the nanocomposite towards MB. Alternative to the Fe_3_O_4_ nanoparticle, Luo et al.^256^ coordinated cubelike Co_3_O_4_ nanoparticles by the solvothermal method and observed equilibrium adsorption capacities of MB and RhB as 128.9 and 47.1 mg/g, respectively. Moreover, Gu et al.^257^ proposed a unique adsorbent having one order of magnitude greater MB adsorption capacity by decorating tiny ZIF-8 nanoparticles within the interlayer of Ti_3_C_2_T_*x*_.

Since 2011, researchers have focused dominantly on one common MXene type as Ti_3_C_2_T_*x*_. However, other members of the MXene family are waiting for their real performances to be discovered for membrane- or adsorption-based separation processes. Only Nb_2_CT_*x*_^216^ was tested for membrane-based separation, whereas there are few studies examining the dye adsorption performance of other MXene types such as Nb_2_CT_*x*_^258,259^ and V_2_CT_*x*_.^260^ Different strategies to modulate the interlayer distance between MXene nanosheets have been discussed up to now. However, the examination of the effect of the etching method in the MXene synthesis on dye separation performance was a weighty matter, which may accelerate the use of a safe and environmentally friendly synthesis approach. Peng et al.^258^ fabricated common MXene (Ti_3_C_2_T_*x*_) and Nb_2_CT_*x*_ by a traditional etching method using HF (t-Ti_3_C_2_T_*x*_ and t-Nb_2_CT_*x*_) and by a hydrothermal etching method using a mixture solution consisting of NaBF_4_ and HCl (h-Ti_3_C_2_T_*x*_ and h-Nb_2_CT_*x*_). SSA of h-Ti_3_C_2_T_*x*_ and t-Ti_3_C_2_T_*x*_ were 44.6 and 8.9 m^2^/g. The MB concentration in solution dropped to 76.4 and 82.7% for h-Ti_3_C_2_ and t-Ti_3_C_2_, respectively, whereas it reduced to 86.2 and 92.4% for h-Nb_2_CT_*x*_ and t-Nb_2_CT_*x*_, proving the efficiency of the hydrothermal method. Although Peng et al.^258^ observed low MB adsorption capacity for Nb_2_CT_*x*_ by either traditional or hydrothermal treatment methods, Yan et al.^261^ reported greater MB and MO adsorption capacities as 99.6 and 100.7 mg/g. Similar performance for MB was also suggested for V_2_CT_*x*_ by Lei et al.^260^ as 111.1 mg/g at 25 °C.

MXene nanomaterials were also fabricated in the form of aerogel to further increase their dye adsorption performances.^259,262^ Ti_3_C_2_T_*x*_ at different loadings was immobilized by SA to form aerogel in order to remove MB.^259^ Comparable adsorption performance was reported for MB as 92.17 mg/g at pH 7 and 25 °C.^259^ However, Feng et al.^262^ reported an excellent CR adsorption capacity of 3568 mg/g for the mixture of Ti_3_C_2_T_*x*_ and PEI, immobilized by SA. It is worth noting that there is not any evidence ascribed to the observed improvement in dye adsorption performance after MXene incorporation into an aerogel during its formation. Therefore, it is not easy to provide the effect of MXene after immobilization on dye removal.

Prominent process design was proposed by utilizing from the potential separation performance of both membrane and adsorbent technology from Kim et al.^263^ For this novel process design, they created a hybrid system by combining Ti_3_C_2_T_*x*_ as an adsorbent with an ultrafiltration membrane (UF) to evaluate its removal performance for MB and MO. In the presence of MB and MO, normalized fluxes of MXene-UF were reported as 0.90 and 0.92, whereas those of single UF were 0.86 and 0.90, respectively. Likewise, when the synthetic dye wastewater was applied including MB (2 mg/L), humic acid, and salts, MXene-UF exhibited a high retention rate of 99.1% with a normalized flux of 0.90. Kim et al.^263^ not only suggested an alternative hybrid model for the separation of dyes but also made it to function as a bridge in transition to membrane technology.

Layered architecture of MXene not only provided shortcuts for enhanced water flux but also improved the removal of dyes thanks to their large amounts of nanochannels with tailorable interlayer distance via intercalation of nanoparticles. Surprisingly, early studies revealed superior permeances for pristine MXene membranes, whereas it could be reached by the following studies later. Nevertheless, the intercalation of some nanoparticles within the MXene nanochannels led to the considerable improvement in water permeances outperforming some commercial and novel nanocomposite membranes.^211,213^ Functionalization was proposed as another strategy not only to adjust the interlayer distance between MXene nanochannels but also to ensure that MXene has a dye-selective ability. However, the real improvement in water permeances was achieved by the synergy between novel polymers and MXene, boosting up the hydrophilicity of the membrane. On the other hand, GO is the nanomaterial that excessively tested with a combination of MXene as a nanocomposite membrane for the removal of dyes to catch the optimum separation performance. The main drawback in dye removal via the membrane process is the fouling phenomena, which was not overcome by MXene membranes despite the exceptions^220,237^ and requires more in-depth studies. Similar strategies were also followed for MXene-based adsorbents, in addition to handling different types of MXene such as Nb_2_CT_*x*_^258,259^ or V_2_CT_*x*_^260^ as adsorbents for dye removal. Collectively, although of all separation applications the most publications have been published on identifying the performance of MXene for dye removal, still there have been some aspects that need to be investigated in detail due to the large differences between dye separation performance in reported studies.

## 5 Separation of Oil-in-Water Emulsions

Unfortunately, industrial wastewater discharges include different types of contaminants that can pose significant long-term risks to human health and environmental safety and need to be removed before discharge. The second-ranking contaminant after dyes, which is produced in huge amounts by many industries, such as textiles, pharmaceuticals, petrochemicals, and metal/steel industries daily, is the oil–water mixtures. Depending on the region, oily wastewater effluent discharge limit is varied within 5–100 mg/L range.^269^ Therefore, finding effective separation or demulsification methods for oily wastewater has gained importance. Alternative to the common separation processes, membrane filtration is proposed for the removal of oil from the aqueous phase. Not only in membrane nanofiltration^229,270−273^ which is considered as the heart of the membrane-based separation processes but also in membrane distillation^130^ and adsorption processes,^273^ the enhancement in separation of oil–water mixtures with the use of MXene nanosheets were proposed. In addition, to enhance the separation performance in these processes, MXene nanosheets were also used to solve the problem related to the concentration polarization, polarized layer, and especially membrane fouling. Since oily wastewater contains highly toxic substances, hydrocarbon compounds, heavy metals, and suspended solid particles, to keep flux rates at a high level throughout the separation process is the main problem in membrane-based oil-in-water-emulsion separation. For instance, Saththasivam et al.^270^ proposed an antifouling membrane, which was fabricated by coating Ti_3_C_2_T_*x*_ nanosheets on conventional print paper. They suggested that favorable characteristic properties of MXene will help to mitigate the fouling by the help of the hydrophilic nature of MXene which increases the interaction with water, leading to the formation of a water layer on the membrane surface and hence the decrease in interaction of membrane with oil.^270^ Their strategy was also confirmed by Tan et al.^130^ who used Ti_3_C_2_T_*x*_ as a coating material to fabricate a membrane via vacuum filtration onto a PVDF support for test in the direct contact membrane distillation (DCMD) process. They compared to the flux decline of PVDF and MXene-coated PVDF membranes after 21 h continuous filtration process using BSA (0.2 g/L) and NaCl (10 g/L) and reported that percentage of flux declines were 18.8 and 8.3% for PVDF and MXene-coated PVDF membranes, respectively.^130^ These preliminary results evidently prove the efficiency of MXene in mitigating the membrane fouling in oil-in-water-emulsion separation. The other purpose of the use of MXene was to benefit from its photothermal property. Tan et al.^130^ also used MXene-coated PVDF membrane to reduce the energy requirement for the DCMD process. While temperature increase for the PVDF membrane was observed approximately 6 °C, it was reported as 49 °C for PVDF coated with MXene. In view of this, Ti_3_C_2_T_*x*_ was suggested as promising nanomaterials to decrease the fouling effect and the energy requirement in addition to gaining an enhancement in separation performance.

The vacuum assisted self-assembly process is the mainly preferred method to prepare free-standing MXene membranes on different polymeric membrane supports. Membrane prepared with this method is widely studied in the oil-in-water-emulsion separation. Additionally, the mostly used MXene type was Ti_3_C_2_T_*x*_ for the fabrication of free-standing membranes. Saththasivam et al.^270^ reported average total fluxes for Ti_3_C_2_T_*x*_ as 544, 682, 649, 638, and 574 L/m^2^ × h × bar for oil-in-water emulsions including sunflower oil, hexane, petroleum ether, silicone oil, and diesel, respectively, with the oil concentration below 12 mg/L. More surprisingly, Li et al.^273^ observed much greater total flux around 6000 L/m^2^ × h × bar without any trace of oil in the permeate side for ultrathin membrane (about 0.03 μm) fabricated by a different kind of MXene (Ti_2_CT_*x*_) on the PES. However, the total flux decreased to 540, 488, and 437 L/m^2^ × h × bar for oil-in-water emulsions including toluene, soybean oil, and pump oil, respectively, with the oil level lower than 10 mg/L, when the tween-80 was used to stabilize the emulsions.^273^ Similar high total fluxes in four kind of oil-in-water emulsions such as toluene, petroleum ether, kerosene, and *n*-hexane were observed for the cracked-earth-like Ti_3_C_2_T_*x*_ membrane surface-enriched with −(OH)_2_ groups.^274^ Much more extreme dye separation performance was achieved for the membrane consisting of free-standing MXene fabricated in the form of nanoribbons by the treatment of KOH.^275^ Its total fluxes of oil-in-water emulsions for gasoline, *n*-hexane, diesel and edible oil were reported around 15,000 L/m^2^ × h × bar for each emulsion.^275^ Disappointingly, its fluxes for each oil-in-water emulsions decreased dramatically after the second cycle.

To reveal the outstanding performance of MXene-based membranes, the more challenging conditions were examined like oil/salt-water emulsion systems. Earlier studies have indicated that the MXene membrane separation performance for oil was deteriorated by the presence of both surfactant and salt in oil–water solution.^273,276,277^ Therefore, in order to fabricate MXene-based membranes preserving its performance in those harsh conditions, different strategies were applied and finally encouraging performances were revealed for MXene membranes. For instance, the effect of different corrosive conditions on the oil–water separation performance of MXene-based membranes was tested in the study of Zhang et al.^271^ They prepared a free-standing MXene (Ti_3_C_2_T_*x*_) membrane on PVDF via cross-linking with SA to identify the separation performance of crude oil–water in acidic (HCl-3M), alkaline (NaOH-3M), and salty (NaCl-3.5 wt %) environments. Its total fluxes increased from 887 to 969, 1043, and 906 L/m^2^ × h × bar at each challenging conditions, respectively, with the oil removal efficiency greater than 99.4%.^271^ Similar analysis was performed for the cracked-earthlike, free-standing Ti_3_C_2_T_*x*_ membrane surface-enriched with −(OH)_2_ groups.^274^ However, total flux was altered from 4720 to 6957, 2796, and 2482 L/m^2^ × h × bar for 1 M NaOH, 1 M NaCl, and 1 M HCl emulsion, respectively.^274^ Although cross-linked MXene^271^ yielded in flux improvement in each emulsions, uncross-linked MXenes^273,274^ revealed a decrease depending on the oil-in-water emulsion system. In order to compare the uncross-linked and cross-linked MXene, Liu et al.^278^ modified Ti_3_C_2_T_*x*_ with 3-aminopropyltriethoxysilane (APTES) and various tannic acid (TA) concentrations for the separation of petroleum ether, lubricating oil, and vegetable oil emulsions. Without sacrificing from the rejection performance (≥98%), more than one order of magnitude enhancement was observed for each emulsion system after cross-linking.^278^ Therefore, we can conclude that the stabilization of MXene layer on the support membrane via cross-linking enhanced the total flux without changing the oil separation efficiency at harsh conditions.

This cross-linking methodology was also used to stabilize the nanocomposite membrane composed of the combination of MXene (Ti_3_C_2_T_*x*_) and reduced graphene oxide (rGO) by Feng et al.^279^ PDA was used as a cross-linking agent to increase the adhesion not only between MXene and graphene but also between nanosheets and support membrane. They designed a MXene/rGO/PDA (160/40/100 mg) nanocomposite membrane to determine separation performance for dodecane, lubricating oil, and petroleum ether stabilized with a sodium dodecyl sulfate (SDS) surfactant. However, total fluxes were reported as 50.03, 56.34, and 72.31 L/m^2^ × h × bar for these oil-in-water emulsions, respectively.^279^ These values are one order of magnitude lower compared to the previous studies due to the formation of oil layer on the membrane surface related to the particle size of oil droplets. Since as a result of cross-linking, the membrane pore size was observed in the order of particle size of oil droplets, oil rejection performance was reported greater than 95% due to the blockage of membrane pores with oil droplets. Promisingly, better performance in terms of total flux was achieved in the combination of MXene with one-dimensional (1D) nanotubes, instead of 2D nanosheets.^272,280^ Total flux of lubricating oil-in-water emulsion reached to 4116 L/m^2^ × h × bar for MXene/halloysite nanotube/PDA (2/5/80 mg)^272^ and 1887 L/m^2^ × h × bar for MXene/aminated-carbon nanotube(ACNT)/APTES (2/10/10 mg)^280^ nanocomposite membranes, exhibiting excellent rejection rates (over 98%).

**Figure 7:**
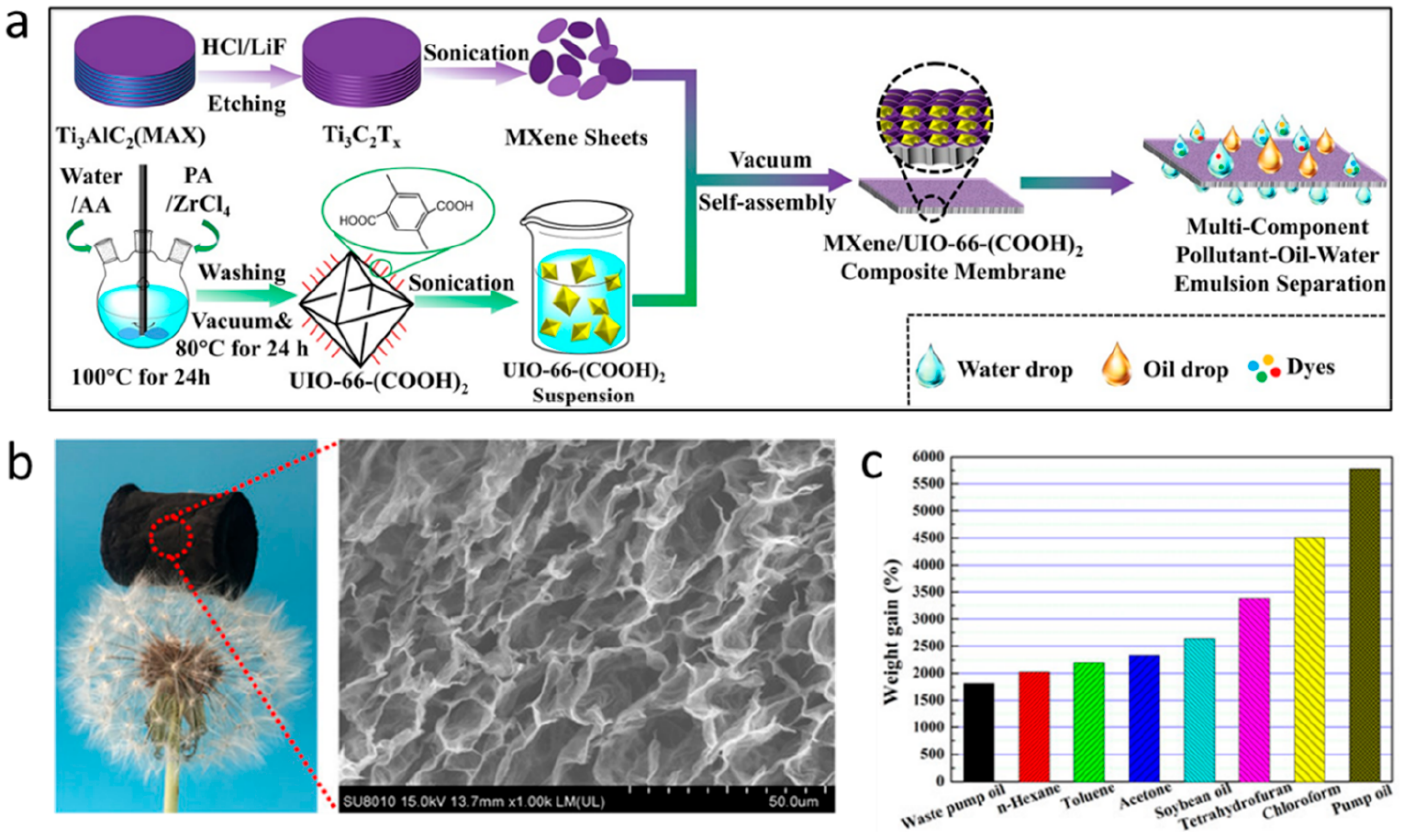
Oil-in-water emulsion separation performance of MXene membranes. (a) Fabrication and separation processes of MXene/UIO-66-(COOH)_2_ nanocomposite membrane for multicomponent pollutant–oil–in-water emulsion. Adapted with permission from ref (214). Copyright 2020 Elsevier. (b) (Left) Photograph and (Right) SEM image of MXene/polyimide hybrid aerogel. Reproduced with permission from ref (288). Copyright 2019 American Chemical Society. (c) Its absorption capacities for various organic liquids. Reproduced with permission from ref (288). Copyright 2019 American Chemical Society.

It is claimed in the literature that MXene nanocomposite membranes were effective for the separation oil-in-water emulsions and dyes simultaneously.^279^ Accordingly, a new question appeared for researchers: “Can we develop a highly effective nanocomposite membrane to separate multi-component pollutants-oil-in-water emulsion even under harsh conditions?” To find an answer to this question, He et al.^214^ synthesized a free-standing nanocomposite membrane composed of the combination of MXene (Ti_3_C_2_T_*x*_) and UiO-66 without cross-linking. They proved that the MXene/MOF nanocomposite membrane can be preferred for the concurrent separation of oil and dye from aqueous solutions [see Figure 7(a)]. While total flux and rejection rates varied between ∼307 and 497 L/m^2^ × h × bar and ∼99.4 and 99.6% for various oil-in-water emulsions, respectively, these properties altered slightly between ∼328 and 488 L/m^2^ × h × bar for various oil-in MB water emulsions and MB rejection rates greater than 99%. Moreover, the MXene/MOF nanocomposite membrane displayed only a slight decrease in total flux (in the order of 7–10%) without a change to its rejection rates even under acidic (HCl-3M), alkaline (NaOH-1M), and salty (NaCl-saturated) environment. Thanks to these studies, which clearly demonstrate the potential of MXene-membranes, we can deduce the competence of MXene membranes in the separation of oil-in-water emulsions as well as dyes even under these very harsh environments, which may represent the actual wastewater conditions in nature. Considering separation properties of MXene-based membranes given in Table 3 for oil-in-water emulsions, we can conclude that MXene-based membranes provide an oil rejection rate greater than approximately 99.5% and total flux of around 500 L/m^2^ × h × bar or higher for specific oils.

**Table 3:** Survey of Oil-in-Water Emulsion Separation Performance of MXene-Based Membranes^a^

membranes	oil-in-water emulsion	rejection (%)	total flux [L(kg*)/m^2^ × h × bar]	ref
Ti_3_C_2_T_*x*_ on print paper (1.2 μm)	sun flower oil	>99	543.5	(270)
Ti_3_C_2_T_*x*_ on print paper (1.2 μm)	hexane	>99	682.3	(270)
Ti_3_C_2_T_*x*_ on print paper (1.2 μm)	petroleum ether	>99	648.7	(270)
Ti_3_C_2_T_*x*_ on print paper (1.2 μm)	silicone oil	>99	638	(270)
Ti_3_C_2_T_*x*_ on print paper (1.2 μm)	diesel	>99	573.7	(270)
Ti_2_CT_*x*_ on PES (0.03 μm)	toluene	―	6149	(273)
Ti_3_C_2_T_*x*_ on PES (0.03 μm)	pump oil	―	6098	(273)
Ti_3_C_2_T_*x*_ on PES (0.03 μm)	soybean oil	―	6115	(273)
Ti_3_C_2_T_*x*_ on PES (0.03 μm)	toluene/Tween 80	99.94	540	(273)
Ti_3_C_2_T_*x*_ on PES (0.03 μm)	pump oil/Tween 80	99.94	437	(273)
Ti_3_C_2_T_*x*_ on PES (0.03 μm)	soybean oil/Tween 80	99.94	488	(273)
Ti_3_C_2_T_*x*_ on PES (0.03 μm)	toluene/Tween 80/salt	99.98	505	(273)
Ti_3_C_2_T_*x*_ on PES (0.03 μm)	pump oil/Tween 80/salt	99.98	392	(273)
Ti_3_C_2_T_*x*_ on PES (0.03 μm)	soybean oil/Tween 80/salt	99.98	465	(273)
Ti_3_C_2_T_*x*_ on PVDF (18.15 μm)	kerosene	99.8	444	(271)
Ti_3_C_2_T_*x*_ on PVDF (18.15 μm)	crude oil	99.7	887	(271)
Ti_3_C_2_T_*x*_ on PVDF (18.15 μm)	heptane	99.5	668	(271)
Ti_3_C_2_T_*x*_ on PVDF (18.15 μm)	hexane	99.4	707	(271)
Ti_3_C_2_T_*x*_ on PVDF (18.15 μm)	petroleum ether	99.6	762	(271)
Ti_3_C_2_T_*x*_ on PVDF (18.15 μm)	crude oil in HCl (3 M)	99.76	970	(271)
Ti_3_C_2_T_*x*_ on PVDF (18.15 μm)	crude oil in NaCl (3.5 wt %)	99.71	905	(271)
Ti_3_C_2_T_*x*_ on PVDF (18.15 μm)	crude oil in NaOH (3 M)	99.72	1045	(271)
Ti_3_C_2_T_*x*_/rGO with PDA on nylon	lubricating oil/SDS	96	56.34	(279)
Ti_3_C_2_T_*x*_/rGO with PDA on nylon	dodecane/SDS	98	50.03	(279)
Ti_3_C_2_T_*x*_/rGO with PDA on nylon	petroleum ether/SDS	98	72.31	(279)
Ti_3_C_2_T_*x*_/UiO-66-(COOH)_2_ on nylon-66	*n*-hexane	99.37	431	(214)
Ti_3_C_2_T_*x*_/UiO-66-(COOH)_2_ on nylon-66	iso-octane	99.63	497	(214)
Ti_3_C_2_T_*x*_/UiO-66-(COOH)_2_ on nylon-66	1,3,5-trimethylbenzene	99.46	344	(214)
Ti_3_C_2_T_*x*_/UiO-66-(COOH)_2_ on nylon-66	toluene	99.55	457	(214)
Ti_3_C_2_T_*x*_/UiO-66-(COOH)_2_ on nylon-66	hexadecane	99.37	422	(214)
Ti_3_C_2_T_*x*_/UiO-66-(COOH)_2_ on nylon-66	crude oil	99.26	307	(214)
Ti_3_C_2_T_*x*_/UiO-66-(COOH)_2_ on nylon-66	toluene in HCl (3 M)	99.33	398	(214)
Ti_3_C_2_T_*x*_/UiO-66-(COOH)_2_ on nylon-66	toluene in NaOH (1 M)	99.46	457	(214)
Ti_3_C_2_T_*x*_/UiO-66-(COOH)_2_ on nylon-66	toluene in NaCl (saturated)	99.52	439	(214)
Ti_3_C_2_T_*x*_/UiO-66-(COOH)_2_ on nylon-66	*n*-hexane/MB	99.65	443	(214)
Ti_3_C_2_T_*x*_/UiO-66-(COOH)_2_ on nylon-66	iso-octane/MB	99.35	488	(214)
Ti_3_C_2_T_*x*_/UiO-66-(COOH)_2_ on nylon-66	1,3,5-trimethyltoluene/MB	99.35	328	(214)
Ti_3_C_2_T_*x*_/UiO-66-(COOH)_2_ on nylon-66	toluene/MB	99.40	446	(214)
Ti_3_C_2_T_*x*_/copolyamide (single layer)	vegetable oil (10 mg/L)	97.44	11,000	(289)
Ti_3_C_2_T_*x*_/copolyamide (single layer)	vegetable oil (100 mg/L)	95.31	8000	(289)
Ti_3_C_2_T_*x*_/copolyamide (single layer)	vegetable oil (1000 mg/L)	97.89	4000	(289)
Ti_3_C_2_T_*x*_/copolyamide (multilayer)	vegetable oil (10 mg/L)	99.0	10,000	(289)
Ti_3_C_2_T_*x*_/copolyamide (multilayer)	vegetable oil (100 mg/L)	98.0	7000	(289)
Ti_3_C_2_T_*x*_/copolyamide (multilayer)	vegetable oil (1000 mg/L)	97.0	3500	(289)
Ti_3_C_2_T_*x*_/TA/APTES on CA (50 μm)	petroleum ether	98.5	4170	(278)
Ti_3_C_2_T_*x*_/TA/APTES on CA (50 μm)	lubricating oil	99.1	4108	(278)
Ti_3_C_2_T_*x*_/TA/APTES on CA (50 μm)	vegetable oil	99.8	3477	(278)
Ti_3_C_2_T_*x*_/ACNT/APTES on CA	lubricating oil	99.6	1887	(280)
Ti_3_C_2_T_*x*_/ACNT/APTES on CA	vegetable oil	99.8	1644	(280)
Ti_3_C_2_T_*x*_/HAL/PDA on CA	petroleum ether	99.9	4241	(272)
Ti_3_C_2_T_*x*_/HAL/PDA on CA	lubricating oil	99.8	4116	(272)
Ti_3_C_2_T_*x*_ (cracked-earthlike and −OH functionalized)	toluene	―	6386	(274)
Ti_3_C_2_T_*x*_ (cracked-earthlike and −OH functionalized)	*n*-hexane	―	4720	(274)
Ti_3_C_2_T_*x*_ (cracked-earthlike and −OH functionalized)	petroleum ether	―	2365	(274)
Ti_3_C_2_T_*x*_ (cracked-earthlike and −OH functionalized)	kerosene	―	464	(274)
Ti_3_C_2_T_*x*_ (expanded with PTFE)	peanut oil	―	5327	(290)
Ti_3_C_2_T_*x*_ (expanded with PTFE)	dichloromethane	―	7482	(290)
Ti_3_C_2_T_*x*_ (expanded with PTFE)	paraffin oil	―	6577	(290)
Ti_3_C_2_T_*x*_ (expanded with PTFE)	toluene	―	8114	(290)
Ti_3_C_2_T_*x*_ (expanded with PTFE)	*n*-hexane	―	8351	(290)
Ti_3_C_2_T_*x*_/ZnO/TA on PEN	petroleum ether/SDS	99.61	2513	(286)
Ti_3_C_2_T_*x*_/ZnO/TA on PEN	isooctane/SDS	99.50	2427	(286)
Ti_3_C_2_T_*x*_/ZnO/TA on PEN	*n*-hexane/SDS	99.43	2412	(286)
Ti_3_C_2_T_*x*_/ZnO/TA on PEN	mesitylene/SDS	99.49	2317	(286)
Ti_3_C_2_T_*x*_/ZnO/TA on PEN	*n*-heptane/SDS	99.47	2196	(286)
Ti_3_C_2_T_*x*_ (coated with PDMS-PDA-PEI) on PVDF	soybean oil	―	9.5–8*	(291)
Ti_3_C_2_T_*x*_ (coated with PDMS-PDA-PEI) on PVDF	soybean oil/SDS	―	12–6*	(291)
Ti_3_C_2_T_*x*_ (nanoribbon) on MCE (38 μm)	gasoline	>99	∼15,000	(275)
Ti_3_C_2_T_*x*_ (nanoribbon) on MCE (38 μm)	*n*-hexane	>99	∼14,500	(275)
Ti_3_C_2_T_*x*_ (nanoribbon) on MCE (38 μm)	diesel	>99	∼15,500	(275)
Ti_3_C_2_T_*x*_ (nanoribbon) on MCE (38 μm)	edible oil	>99	15,860	(275)
Ti_3_C_2_T_*x*_ /BN/PDA/PEI (5.68 μm)	1,2-dichloroethane	96.54	95.8	(287)
Ti_3_C_2_T_*x*_ /BN/PDA/PEI (5.68 μm)	dichloromethane	98.83	326	(287)
Ti_3_C_2_T_*x*_ /BN/PDA/PEI (5.68 μm)	hexane	94.90	875	(287)
Ti_3_C_2_T_*x*_ /BN/PDA/PEI (5.68 μm)	chloroform	95.50	144	(287)
Ti_3_C_2_T_*x*_ /BN/PDA/PEI (5.68 μm)	toluene	96.46	77.5	(287)

a Membrane thickness is given in parentheses. ACNTs: amine functionalized carbon nanotubes, APTES: 3-aminopropyltriethoxysilane, BN: boron nitrate, CA: cellulose acetate, HAL: halloysite nanotube, MB: methylene blue, MCE: mixed cellulose, PDA: polydopamine, PDMS: polydimethylsiloxane, PEI: polyethylenimine, PEN: poly(arylene ether nitrile), PES: polyether sulfone, PTFE: polytetrafluoroethylene, PVDF: polyvinylidene difluoride, SA: sodium alginate, TA: tannic acid.

Challenges in oil-in-water emulsion separation such as to observe high flux and rejection rate, less fouling, and great chemical stability were targeted to be overcome by designing MXene-based nanocomposite membranes. However, the nanofiltration process is unfeasible in the case of big environmental pollution where large amounts of oil were spilled in lakes, rivers, or ocean. To separate huge volumes of oil spillage from water sources quickly and effectively, absorbents such as sponges and aerogels are considered a novel approach. There are few studies in the literature that used MXene-based adsorbents to evaluate their oil–water separation performance. Wang et al.^281^ used Ti_3_C_2_T_*x*_ to create the MXene/polyimide hybrid aerogel with very low density and high porosity [see Figure 7(b)]. Among the absorbents, aerogels such as polyimide aerogels are the most promising materials due to their excellent compressible features and thermal stability. Different organic liquids such as pump oil, chloroform, tetrahydrofuran, soybean oil, acetone, toluene, *n*-hexane, and pump oil were applied to determine the absorption capacity of the MXene/polyimide hybrid aerogel. The highest percentages of weight gain (absorption capacities) were reported as 5778 and 4508% for pump oil and chloroform, respectively, as given in Figure 7(c). Its performance was compared with the graphene/polyimide aerogel^281^ which absorbed motor oil 37 times its own weight, whereas the MXene/polyimide hybrid aerogel could absorb various organic liquids from ∼18 to ∼58 times its own weight.^281^ Thanks to the novel strategies to fabricate aerogel for oil-in-water emulsion separation, MXene was adapted successfully. For instance, melamine sponge (MS) covered with tetradecylamine (TDA)-functionalized MXene revealed absorption capacity ranges from 60 to 112 times of its own mass.^282^ Wood-inspired MXene ternary aerogels synthesized with a novel approach and composed of nanocrystal cellulose functionalized with the silane agent displayed absorption performance varying between 45 and 63 times its own weight for several oil-in-water emulsions.^283^ Due to the hybrid hydrophobic–hydrophilic surface characteristics of these novel aerogels, superior characteristic features in addition to separation performance were achieved. Moreover, since MXene nanomaterials can possess an extensive temperature limit, they have great potential for the applications in photothermal-assisted oil recovery.^283−285^ Collectively, all these studies about MXene-based hybrid aerogels paved the way for the future absorbent studies.

Durability and long-term operation stability of the membrane are also crucial for the separation of the oily wastewater even under harsh conditions. Therefore, similar to other membrane-based separation applications, durability and stability of MXene membranes in oil-in-water emulsion separation were tested by all studies included. For instance, Saththasivam et al.^270^ reported a 13% decrease in total flux when sunflower oil was used in feed solution after 8 cycles of filtration with the stable oil rejection of >99% and did not observe any sign of degradation after operation/washing cycles. Similarly, the water flux of the MXene/ACNT/APTES nanocomposite membrane decreased 26% after the 8^th^ cycle but still had high performance as 2284 L/m^2^ × h × bar.^280^ Emulsion separation flux of the MXene/ZnO nanocomposite membrane decreased 27% after the 10^th^ cycle, revealing the high performance as 1838 L/m^2^ × h × bar and >99% petroleum ether rejection.^286^ However, fortunately, Li et al.^273^ did not achieve any decrease either in total flux or rejection rate even after 50 consecutive cycles for the Ti_2_CT_*x*_ membrane. Similarly, no considerable changes were reported in the study of Zhang et al.^271^ where only kerosene/water emulsion was investigated for 10 cycles. Feng et al.^279^ proved the long-term stability and durability of the MXene membrane by washing with ethanol for 3 days and immersing in strong acidic or alkaline solutions for more than three months. Similarly, excellent durability after being immersed in water for 600 h was achieved for MXene/BN/PDA/PEI nanocomposite membrane fabricated by Zhang et al.^287^ Ten washing/operation cycles were also performed by He et al.^214^ for toluene-in-water emulsion, and a 2% increase in total flux and rejection rate drop from 99.6 to 99.4% were observed for the MXene/MOF membrane. Moreover, even after dipping into acidic, alkaline, and salty solutions for 8 h, the MXene/MOF nanocomposite membrane preserved its separation performance, confirming its excellent chemical stability under harsh conditions. A different stability and reusability issue was observed for the MXene/polyimide hybrid aerogel adsorbent proposed by Wang et al.^281^ Its absorption capacity for soybean oil after 10 cycles was kept nearly stable even under harsh conditions as treating with soybean oil at 400 °C in air for 1 h. However, when it was treated with soybean oil in liquid nitrogen for 10 min, its performance was deteriorated after the 5^th^ cycle due to the occurrence of fractures during the freezing process leading to the increase in the permeation of oil molecules. Interestingly, while reusability of MXene/polyimide hybrid aerogel was possible at high temperatures, it was restricted at low temperatures due to the brittle structure of aerogel. However, absorption capacity of *n*-hexane and silicone oil decreased only as ∼8 and 10% for MXene/TDA/MS aerogel after 20 cycles.^282^ For super heavy crude oil, adsorption capacity of the ternary MXene aerogel dropped only 23.8% after 5 cycles.^283^ In accordance with these studies, long-term operation and chemical stability of MXene membranes and absorbents were only revealed for specific oil-in-water emulsions and for the limited number of cycles where the highest one was 50. To mesmerize the membrane market, many more cycles for MXene membranes should be tested for several oil-in-water emulsion systems.

Since the fouling phenomena is a vital factor in the oil-in-water separation membrane due to limiting its durability and long-term operation stability, MXene nanomaterials were offered as a crucial solution to alleviate the fouling problem, utilizing their hydrophilic nature. Great performance was reported for free-standing MXene membranes cross-linked with different agents,^271,278^ free-standing MXene-based nanocomposite membranes composed of carbon-based nanomaterials and stabilized with cross-linking,^229,272,280^ and MXene-based adsorbents in the form of sponge and aerogel.^281−283,288^ However, there are a very limited number of studies investigating the oil-in-water separation performance of MXene. Instead, the ones published evidently proved its superior capability of oil-in-water separation even under the more challenging conditions like oil/salt-water emulsion systems as well as oil/dye-water emulsion systems at very harsh environments.

## 6 Heavy Metal Ion Removal

The other contaminant that leads to the environmental pollution and threatens all living organisms is the heavy metals. Especially, heavy metal wastes arisen from the developed industries in the 21^st^ century such as mining, metallurgy, leather tanning, electronics, and chemicals pose a serious threat. Heavy metals are toxic for organisms as they cause the formation of free radicals. More importantly, heavy metals easily accumulate in a human body and are not biodegradable. To date, various removal methods for heavy metal ions are applied such as chemical precipitation, coagulation, photocatalytic degradation, solvent extraction, adsorption, and membrane filtration.

### 6.1 Adsorption-Based Separation

Within the heavy metal ion removal processes, adsorption is a highly used process. The MXene family has become one of the strongest competitors to the existing adsorbents due to their high surface area and tailorable surface chemistry. MXene nanomaterials are tested dominantly as adsorbents for the removal of several heavy metal ions such as Cd(II),^254,292−294^ Cr(VI),^143,262,294−302^ Cu(II),^293,294,303−307^ Hg(II),^292,308−310^ Ni(II),^311^ and Pb(II)^306,307,312−316^ in the literature. Different mechanisms have been proposed to explain the advanced adsorption performance of MXene. The principal adsorption mechanism of the MXene nanomaterial for heavy metal ions was proposed as electrostatic attraction between adsorbent and ions.^313^ Oxidation of ions to their low oxidation states on the surface of MXene was proposed as a supporting mechanism for the enhancement of adsorption performance of MXene.^143,300^ Additionally, inner-sphere complexation which consists of pH-dependent electrostatic interaction is the other mechanism used to explain the superior performance of MXene.^312,317^ The final mechanism proposed for the identification of performance of MXene is the ion-exchange.^312,313^ However, mostly, the removal performance of MXene is explained based on the mutual effect of several mechanisms. The heavy metal ion adsorption mechanism was also aimed at being identified by first-principles calculations.^318^ Preliminary studies motivated the use of MXene nanomaterials as adsorbents for the removal of heavy metal ions by displaying their outstanding adsorption performance. For instance, Ti_3_C_2_T_*x*_ having SSA of 57 m^2^/g revealed the removal capacity of Cr(VI) as 250 mg/g.^143^ Its removal performance of bromate (BrO_3_^–^) was suggested as 321.8 mg/g with instant 100% BrO_3_^–^ removal from drinking water.^317^ Additionally, adsorption performance of MXene for several heavy metal ions was compared with the common adsorbents.^295,312^ Jun et al.^312^ compared the adsorption efficiency of Ti_3_C_2_T_*x*_ with the powder activated carbon (PAC) for Pb(II) ions. Removal rate of MXene was reported as ∼92% while it was ∼68% for PAC, although MXene had less surface area as ∼10 m^2^/g than PAC (∼470 m^2^/g).^312^ This was explained by the electrostatic interaction between the negatively charged MXene surface and Pb(II) ions along with the mechanisms of ion-exchange and inner-sphere complex formation.^312^ Karthikeyan et al.^295^ suggested that adsorption capacity of Ti_3_C_2_T_*x*_ for Cr(VI) (104 mg/g) was greater than the common adsorbents as ceramic materials. Additionally, composite adsorbents composed of different materials modified with MXene have led to an increase in removal performance. For instance, adsorption performance of alginate modified with PEI and −NH_2_ functionalized Ti_3_C_2_T_*x*_ (550.3 mg/g)^262^ for Cr(VI) was comparably higher than both pure alginate beads modified with PEI (375.3 mg/g)^319^ and alginate modified with PEI and Fe_3_O_4_ (175.8 mg/g).^320^ With these exciting studies, the MXene family started to be tested for adsorption of other heavy metal ions as listed at Table S5, and several strategies are proposed to improve their adsorption performance.

**Figure 8:**
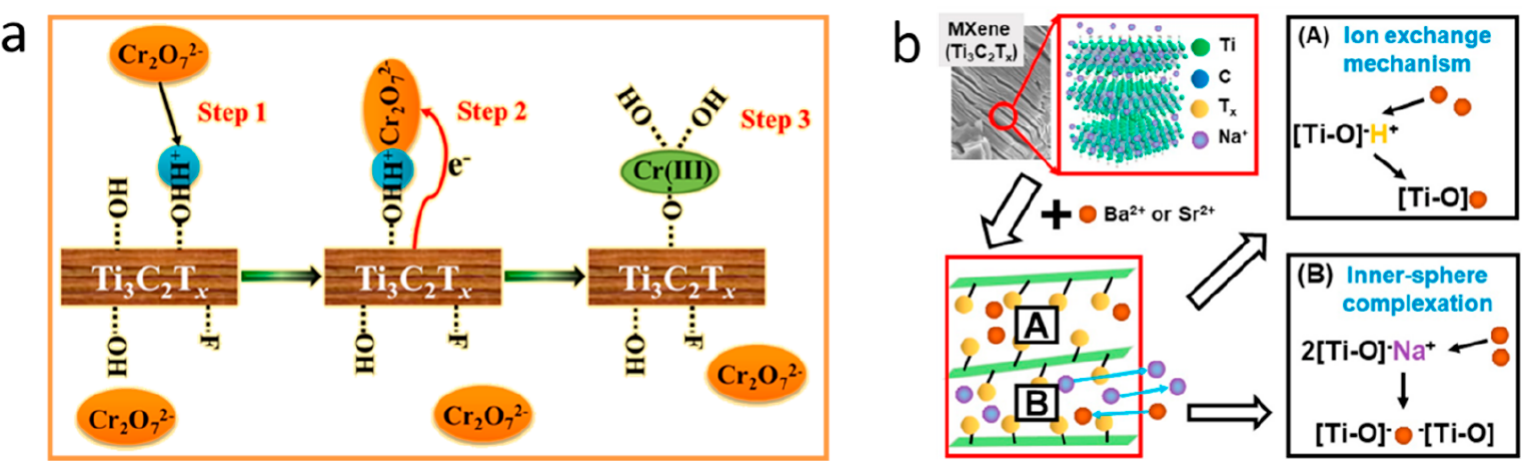
Schematic illustration of the removal mechanisms of Ti_3_C_2_T_*x*_ for (a) Cr(VI) and (b) Ba(II) or Sr(II). Panel (a): Adapted with permission from ref (143). Copyright 2015 American Chemical Society. Panel (b): Adapted with permission from ref (321). Copyright 2020 Elsevier.

One of the strategies preferred to enhance the adsorption capability of MXene is the functionalization of the MXene surface, benefiting from its tailorable surface chemistry. Amino-functionalization of Ti_3_C_2_T_*x*_ led to a 44.2% increase in its adsorption capacities for total Cr.^297^ Functionalization with the silane coupling agent enhanced the 205% Pb(II) adsorption capacity of Ti_3_C_2_T_*x*_.^315^ Likewise, insertion of the enzymatic hydrolysis lignin as a biosurfactant into the Ti_2_CT_*x*_ yielded an increase of 50.2% in Pb(II) adsorption capacity.^313^ In another study, alkaline-treated Ti_3_C_2_T_*x*_ having intercalated with Na(I) ions displayed a superior Pb(II) adsorption capacity (∼140 mg/g) and surprisingly instant equilibration within only 2 min.^314^ Zhang et al.^307^ offered to combine the above purposed methods as alkaline treatment and amino-functionalization for the removal of Pb(II). As expected, the highest Pb(II) adsorption capacity (see Table S5) along with the lowest equilibrium time of 20 min was correlated with this synergetic modification compared to the performance of the above-mentioned MXene adsorbents modified with different methods. Comparably, modified Ti_3_C_2_T_*x*_ displayed a 69.2% improvement in the Pb(II) adsorption capacity accompanying the enhancements in interlayer spacing from 8.8 to 13.6 Å and surface area from 6.37 to 129.2 m^2^/g.^307^ Collectively, since the interlayer spacing of MXene increased after functionalization, it was claimed that this provided more active adsorption sites leading to the strong van der Waals forces and electrostatic interaction along with effective exchange of ions, as illustrated in Figure 8(a).^307,313−315^

Fabrication of MXene-based composite adsorbents with different polymers was considered to be another effective strategy for removing heavy metal ions. Either amino acid-based or petroleum-based polymers are used to observe composite materials. To improve the efficiency of MXene for Cr(VI), the Ti_3_C_2_T_*x*_/poly(m-phenylenediamine) (PmPD) composite was produced via functionalization of Ti_3_C_2_T_*x*_ through in situ polymerization and then intercalation of PmPD.^296^ Thanks to the synergistic effect between Ti_3_C_2_T_*x*_ and PmPD, the removal capacity of Cr(VI) was observed as 540.47 mg/g which was greater than the performance of pure PmPD of 384.73 mg/g and pristine Ti_3_C_2_T_*x*_ of 137.45 mg/g (40.5 and 293%, respectively).^296^ This was attributed to the increase in interlayer spacing from 14.6 to 17.6 Å and the specific surface area from 10.42 to 55.93 m^2^/g compared to the pristine MXene.^296^ Similarly, the MXene/polymer composite adsorbent was prepared via in situ growth of imidazoles on the surface of MXene, and its adsorption performance of Cr(VI) was examined.^322^ Although its adsorption performance for Cr(VI) was not greater than the result of the previous study,^296^ its adsorption capacity quickly increased to 66.91 mg/g within 1 min and reached to the equilibrium point of 119.5 mg/g at the end of the 80 min.^322^ On the other hand, amino acid polymerization, then coating Ti_3_C_2_T_*x*_, and intercalation of amino acid into the Ti_3_C_2_T_*x*_ improved the Cu(II) adsorption capacity by 83.6%^303^ and 20.6%^304^ compared to the pristine MXene,^303,305^ respectively. Additionally, the nanocomposite fabricated using MXene and alginate consisting of several amino groups revealed the increased Cu(II) and Pb(II) adsorption rates of 92.1 and 114.3%, respectively, with the increase in alginate concentration from 30 to 70% in a composite adsorbent.^306^

Unprecedented Hg(II) removal performance was reported for MXene-based adsorbents compared to other heavy metal ions.^292,308−310^ Hg(II) adsorption performance of pure SA was compared with GO/SA and Ti_3_C_2_T_*x*_/SA nanocomposites by the group of Lee. Adsorption rates of GO/SA and pure SA for Hg(II) were 34.63 and 11.53%, respectively, whereas that of MXene/SA was 100% with the maximum adsorption capacity of 932.84 mg/g.^292^ In their following study, they observed an excellent adsorption capacity of 1128.41 mg/g with the deposition of Fe_2_O_3_ nanoparticles on the surface of MXene, instead of having lower SSA of 56.51 m^2^/g than the pristine MXene (63.39 m^2^/g).^310^ Hg(II) removal rates of pristine Ti_3_C_2_T_*x*_ and Ti_3_C_2_T_*x*_/Fe_2_O_3_ nanocomposite were 58.92 and 99.27%. Similarly, molybdenum disulfide (MoS_2_) was deposited on the surface of MXene to further improve its Hg(II) adsorption capacity by the same group.^309^ With the fabrication of delaminated MXene/MoS_2_ nanocomposites, they observed the greatest adsorption capacity of 1435.2 mg/g with the removal efficiency of 98.5% for Hg(II).^309^ This exceptional Hg(II) removal of the nanocomposite was not attributed to an only distinct adsorption capacity but also to the catalytic reduction. Fu et al.^308^ unveiled the tremendously high Hg(II) maximum adsorption capacity (4806 mg/g) of oxygen-functionalized Ti_3_C_2_T_*x*_ and linked this performance with the ability of {001}-Ti edge and oxygen functional groups that enable catalytic reduction and electrostatic interaction, respectively. Similar Hg(II) removal performance was reported for Ti_3_C_2_T_*x*_ (5070 mg/g) by Shahzad et al.^323^ and compared with the maximum adsorption capacity of Ti_3_CNT_*x*_ (4263 mg/g). The achievement of high Hg(II) adsorption in MXene-based nanocomposites encouraged researchers to combine MXene with different materials for the removal of other heavy metal ions.

Encouraging from the performance of MXene-based nanocomposites for Hg(II) removal, novel MXene-based nanocomposite designs were proposed as potential candidates for the removal of other heavy metal ions.^143,298,299^ Alkaline-treated Ti_3_C_2_ having intercalated nanoscale zerovalent iron (nZVI) within its nanochannels revealed a 537% improvement in Cr(VI) adsorption capacity from 30.6 to 194.87 mg/g.^299^ This high adsorption capacity was ascribed to the complex adsorption mechanism where negatively charged Cr(VI) ions adsorbed onto the MXene surface, Cr(VI) reduced to the Cr(III), and Fe-O-Cr(III) species were formed simultaneously.^299^ Alternatively, TiO_2_ nanoparticles were distributed regularly between MXene nanochannels for the removal of Cr(VI).^143^ As a result of strong electrostatic interaction and reduction of Cr_2_O_7_^2–^ to Cr (III), the removal rate of the Ti_3_C_2_/TiO_2_ nanocomposite for Cr(VI) was achieved as 97.7%.^143^ TiO_2_-C/TiC nanocomposite derived from Ti_3_C_2_(OH)_0.8_F_1.2_ revealed higher adsorption capacity of Cr(VI) as ∼225 mg/g with the removal rate of >95% compared to the precursor MXene (∼62 mg/g).^298^ Similarly, novel nanocomposite adsorbents were also proposed for Ni(II) removal. Feng et al.^311^ synthesized the Ti_3_C_2_T_*x*_/layered double metal hydroxide (LDH) nanocomposite having maximum adsorption capacity of 222.7 mg/g with the removal rate of >97.35%. However, adsorption capacities of pristine LDH (38.95 mg/g) and MXene (52.86 mg/g) for Ni(II) were well-below the performance of the nanocomposite adsorbent, which was attributed to the presence of multimolecular layer adsorption. Since impressively high improvements were reached for heavy metal ion removal using novel MXene-based nanocomposite materials, this evidently highlights the importance of engineered structures like the layered 2D–2D heterogeneous nanoplatelets for the adsorbent materials.

The final identified strategy is the morphology of adsorbents. There are contradicting observations about the effect of morphology in the literature. For instance, Gu et al.^316^ suggested that Ti_3_C_2_T_*x*_ produced as nanofibers displayed the highest maximum Pb(II) adsorption capacity of 285.9 mg/g compared to the commercial MXene nanosheets. This was attributed to the enhanced surface area of 16.4 m^2^/g and plenty of oxygenated functional groups compared to its nanosheet form. However, Shahzad et al.^292^ reported that the Ti_2_CT_*x*_ produced as nanosheets displayed higher maximum adsorption capacity of Cd(II) as 294.23 mg/g than its nanofiber form. A similar reason was claimed in this study to explain the high performance of the nanosheet form with a higher surface area of 66.7 m^2^/g compared to the nanofiber form (55.1 m^2^/g) of MXene. The main difference in the fabrication of MXene sheets/fibers from the corresponding MAX phase is that while the former played with the temperature by keeping molarity of NaOH constant in solution, the later changed the NaOH molar value at constant temperature to form either nanosheets or nanofibers. However, the Ti_2_AlC MAX phase was produced via a bottom-up approach by Shahzad et al.^292^ while Gu et al.^316^ used a top-down method for the fabrication of Ti_3_C_2_T_*x*_ nanofibers and nanosheets. In another study, by playing with the alkalization process, Ti_3_C_2_T_*x*_ was also fabricated in the form of nanofibers and nanoribbons using NaOH and KOH solutions, respectively.^324^ NaOH alkalization led to an urchinlike nanofiber morphology with a surface area of 62.4 m^2^/g, whereas KOH alkalization was formed as a kelplike nanoribbon with a surface area of 57.8 m^2^/g. Therefore, maximum adsorption capacity of the former for Pb(II) (328.9 mg/g) was greater than that of the latter (248.3 mg/g).^324^ Additionally, Dong et al.^293^ fabricated the precursor (Ti_3_AlC_2_, MAX phase) of MXene in a nanofiber form and tested its Cd(II) and Cu(II) adsorption capacities. Even if the heavy metal adsorption capacity of the precursor of MXene cannot reach the performance of MXene, the comparison of the nanofiber form with the original morphology of the MAX phase evidently revealed the effect of morphology on adsorption performance. While the commercial MAX phase had 4.28 and 6.57 mg/g adsorption capacity for Cu(II) and Cd(II), its nanofiber form revealed those of 11.13 and 11.50 mg/g, respectively.^293^ To sum up briefly, the morphology which increased the surface area and, hence, the number of active adsorption sites is more capable of improving the capacity of MXene for heavy metal ion removal.

Unfortunately, different heavy metal ions coexist in wastewater, which complicates the removal process. Heavy metal ion adsorption capacities of MXene nanomaterials were examined with the coexisting ions in the solution in order to reveal their combined effect on the removal of heavy metal ions. For instance, Cu(II) removal efficiency (∼98%) of delaminated Ti_3_C_2_T_*x*_ was not affected from the presence of common heavy metal ions of Pb(II), Cr(III), and Cd(II) in the wastewater.^305^ Likewise, 98% removal efficiency of Ti_3_C_2_T_*x*_ for Ba(II) ions was reported when other metal ions such as As(III), Pb(II), Cr(IV), Ca(II), and Sr(II) were present in the simulated solution.^325^ Similarly, Ti_3_C_2_T_*x*_ kept the removal rate of Hg(II) as nearly 100% with coexisting heavy metal ions such as Pb(II), Cd(II), Ni(II), and Cu(II), while its removal rates for other metal ions were only 47.8, 18.4, 9.8, and 7.6%, respectively, proving high selectivity of Hg(II).^326^ Slight drops for alkaline-treated and nZVI-intercalated Ti_3_C_2_T_*x*_ were reported where 54% removal efficiency of Cr(IV) was decreased only 2.23% for wastewater, including Ca(II), Mn(II), Zn(II), and Ni(II) coexisting ions.^299^ However, when single and mixture adsorption performances of Ti_3_C_2_T_*x*_ were compared in another study, 5.5, 38.0, 71.4, and 82.9% reductions in the removal efficiencies of Pb(II), Cu(II), Zn(II), and Cd(II) were reported, respectively, which follows the heavy metal hydroxide constants (log *K* values).^299^ On the contrary, Ti_3_C_2_T_*x*_ having large interlayer spacing and abundant adsorption sites due to the alkaline treatment revealed high reduction in Pb(II) removal efficiency as 37.1 and 33.9% in the presence of Ca(II) and Mg(II), respectively.^314^ Encouraging data obtained from the studies identifying the effect of heavy metal ion mixture may lead researchers to investigate the use of MXene in the industrial field.

The studies reviewed so far have revealed that MXene has high adsorption performance for heavy metal ions. However, demonstrating its industrial applicability is as important as adsorption capacity. In order to determine the real performance of MXene as an adsorbent in the real wastewater environment, a simulated water environment, which includes various competing ions, was applied to the MXene and MXene-based nanocomposite adsorbents in several studies. Gu et al.^316^ treated the layered Ti_3_C_2_T_*x*_ nanofiber (single Pb(II) adsorption capacity of 285.9 mg/g) with the simulated drinking water consisting of Ca(II) (40 mg/L), Mg(II) (80 mg/L), Na(I) (100 mg/L), and Pb(II) (0.1 mg/L). Average treatment capacity was 2500 kg water/kg nanofiber with a Pb(II) concentration of ∼0.008 mg/L, which is under the drinking water standard (0.01 mg/L) at pH 6.8–7.2. Similarly, Zou et al.^298^ treated the urchinlike TiO_2_-C/TiC nanocomposite (single Cr(VI) adsorption capacity of 225 mg/g) with synthetic wastewater including 2 mg/L of Cr(VI) and 200 mg/L of Cl(I), NO_3_(I), and SO_4_(II) at pH 5.5–6.8. They unveiled the treatment capacity of 1120 kg water/kg adsorbent for this nanocomposite with 0.1 mg/L of Cr(VI), which is the wastewater discharge standard in China.^298^ However, the treatment capacity was observed as nearly 800 kg water/kg adsorbent with 0.1 mg/L of Cr(VI) when real wastewater was used.^298^ Shahzad et al.^292^ prepared simulated groundwater containing Na(I) (0.1 mg/g), Mg(II) (0.08 mg/g), and Ca(II) (0.04 mg/g) ions to investigate adsorption capacity of the Ti_2_CT_*x*_ nanofiber and nanosheet for Cd(II) removal. They reported excellent removal rates of 99.47 and 99.70% with the final Cd(II) concentration of 1.87 × 10^–6^ and 1.04 × 10^–6^ mg/g for the Ti_2_CT_*x*_ nanofiber and nanosheet, respectively, which is in accordance with the US Environmental Protection Agency limit (EPA) (5 × 10^–6^ mg/g).^292^ Likewise, the Ti_3_C_2_T_*x*_/MoS_2_ nanocomposite preserved its high Hg(II) removal efficiency of 99.75% in the presence of other metal [Pb(II), Cd(II), Cr(III), Cu(II), and Zn(II)] and salt [Mg(II), Na(I), Ca(II), and K(I)] ions in the simulated wastewater.^309^ The same Hg(II) removal performance was also reported for multilayered oxygen-functionalized Ti_3_C_2_T_*x*_ where Hg(II) removal efficiencies were varied between 99.6 and 97.5% in the presence of separate salt ions as Mg(II), Na(I), Ca(II), and K(I).^308^ However, a desperate situation is valid for the Ti_3_C_2_T_*x*_/PEI/SA aerogel which has the highest Cr(VI) adsorption capacity within the identified MXene-based adsorbents in Table S5.^262^ Cr(VI) adsorption capacity decreased to 22.1 and 91.8% at low (0.001M) and high (0.1M) anion concentrations [Br(I), Cl(I), SO_4_(II), CO_3_(II)], respectively, whereas 14.8 and 41.0% drops were reported for two different cation concentrations [Mg(II), Na(I), Ca(II), and K(I)].^262^ Nevertheless, according to the above-discussed studies, considering the treatment capacity of water above 1000 kg with the removal efficiency of >99% for different heavy metal ions, MXene-based materials emerge as brilliant adsorbents for removing heavy metal ions from complex wastewater compositions.

The removal and recovery of precious metal ions from wastewater are necessary not only environmentally but also economically. Adsorption performance of MXene-based adsorbents and films for several precious metal ions such as Pd(II),^300,327^ Au(III),^300,328,329^ and Ag(I)^300,329^ were identified in the literature. For instance, the Ti_3_C_2_T_*x*_/rGO nanocomposite was fabricated as a film, and its adsorption capacity of Au(III) reached to 1241 mg/g.^300^ Considerable alteration was not reported for Au(III) adsorption (1063.8 mg/g) in another study^329^ where Ti_3_C_2_T_*x*_/rGO was fabricated in an aerogels form. However, the Au(III) adsorption capacity of the Ti_3_C_2_T_*x*_/CNT nanocomposite film was reported as 2093 mg/g.^328^ The introduction of either rGO or CNT leads to the restacking of Ti_3_C_2_T_*x*_ and, hence, gaining high SSA. To explain the Au(III) adsorption process of the nanocomposite, the redox rejection mechanism was suggested where oxidation of MXene and reduction of Au(III) occurred at the same time.^328^ Additionally, while the Ti_3_C_2_T_*x*_ adsorbent treated at 45 °C revealed Pd(II) adsorption capacity of 185 mg/g,^327^ rGO intercalated Ti_3_C_2_T_*x*_ film displayed enhancement in the Pd(II) adsorption capacity (890 mg/g).^300^ Although this high Pd(II) adsorption performance was attributed to the rGO intercalation in order to hinder the restacking,^300^ it is worth noting that there is not any stage as exfoliation, which is performed in the film fabrication and increases the surface area of MXene, in adsorbent preparation aside from the etching stage.

In addition to the high removal performances of MXene for heavy metal ions, long-term applicability and reusability of MXene nanomaterials were mostly tested within several continuous cycles. Shahzad et al.^309^ unveiled the high stability of the Ti_3_C_2_T_*x*_/MoS_2_ heterogeneous nanocomposite for Hg(II) adsorption. Nanocomposite treated with 5 M HCl for 3 h revealed the adsorption and desorption rates of Hg(II) as 100 and 37.3% after five consecutive cycles, respectively. However, as contact time increased to 30 h, the adsorption rate remained constant whereas the desorption rate reached to 55.0%. Likewise, alkaline-treated MXene/LDH nanocomposite fabricated by Feng et al.^311^ exhibited a superior Ni(II) adsorption rate of >85.32% even after eight continuous adsorption–desorption cycles at 25 °C. The maximum adsorption/desorption cycle number was carried out by Dong et al.^306^ where they tested Pb(II) and Cu(II) adsorption rates of cross-linked and uncross-linked Ti_3_C_2_T_*x*_/alginate adsorbents within 10 continuous cycles. Following the cleaning process of Ti_3_C_2_T_*x*_/alginate by 0.1 M nitric acid solution, only 8.9 and 5.4% drops were observed in the adsorption rates of the cross-linked adsorbent for Pb(II) and Cu(II), respectively, while those of the uncross-linked one were 13.1 and 12.7%. Since the structural stability is preserved with the cross-linking, this also leads to excellent regenerability, in other words, the stability in heavy metal ion removal performance. Considering pristine MXene, rather than the composite, Hg(II) removal performance of Ti_3_C_2_T_*x*_ exhibited only a 9% decline after 5 consecutive adsorption–desorption cycles,^323^ which is still promising. However, the removal of Hg(II) for the other type of MXene, Ti_3_CNT_*x*_, decreased almost 25% under the same conditions.^323^ Unfortunately, a desperate situation is present in the regenerability test of delaminated MXene treated with an acidic mixture including nitric acid and calcium nitrate for 5 h after centrifugation.^305^ The removal rate of Cu(II) was 80% in the first cycle; however, in the second and third cycles, the removal rate dropped to 47 and 30%, respectively, due to the formation of TiO_2_ and incomplete Cu(II) desorption after the regeneration process. They suggested that the ion-exchange reaction between Cu(II) and the surface functional groups of MXene occurred during the adsorption process. As a result of the ion-exchange mechanism which was generally named as the inner-sphere complex formation, the formation of CuO_2_ via the reduction of Cu(II) was observed, proving the existing challenge in desorption process. Similarly, the Cr(VI) removal rate of the Ti_3_C_2_T_*x*_/δ-MnO_2_ nanocomposite adsorbent sharply changed from 86 to 45% after five consecutive cycles, since the adsorption is partly governed by the chemisorption process, which hinders the regeneration of adsorbent at mild conditions.^330^

### 6.2 Membrane-Based Separation

Although MXene-based nanomaterials were dominantly used as adsorbents for heavy metal ion removal, its usage is limited in the membrane technology. MXene/Fe_3_O_4_ nanocomposite membranes, which were fabricated using various amounts of Fe_3_O_4_ nanoparticles, displayed a high water flux of 125.1 L/m^2^ × h due to the enhanced interlayer spacing. Likewise, moderate rejection values were determined as 63.2, 64.1, and 70.2% for Cu(II), Cd(II), and Cr(VI), respectively.^294^ This was attributed to the accessible −(OH)_2_ groups on the surface of the nanocomposite membrane with the insertion of nanoparticles. Similarly, MXene/CNT nanocomposite membranes were identified for the separation of Au(III).^328^ Encouraging results were reported where water permeability under a pressure of 1.0 bar was 437.6 L/m^2^ × h × bar, and Au(III) rejection was 99.8%.^328^ The high rejection performance was attributed to the spontaneous electron transfer ability of the nanocomposite material, leading to the reduction of Au(III) to zerovalent Au nanoparticles and, hence, easy rejection of Au nanoparticles through the nanochannels of MXene due to the size-sieving. Collectively, the proper use of MXene as a membrane material alternative to the adsorbent material is not a negligible strategy in heavy metal ion removal and requires further investigation.

Due to the high surface area and designable surface chemistry, the removal efficiency of the MXene family for heavy metal ions of Cd(II), Cr(VI), Cu(II), Hg(II), Ni(II), and Pb(II) was dominantly investigated and adsorption mechanisms under this performance were aimed to be revealed in-depth. Instead of a unique separation mechanism, it was suggested that the removal performance of MXene can be explained by the mutual contribution of electrostatic interaction, oxidation of ions, inner-sphere complexation, and ion-exchange. Similarly, the interlayer distance was accepted as a key point for MXene nanomaterials and aimed to tune via functionalization of the MXene surface and fabrication of the MXene-based composite adsorbents with different polymers. The most encouraging conclusion was derived as the instant equilibration (within only 2 min) of the MXene adsorbent during adsorption of specific heavy metal ions, suggesting the opportunity to carry out a great number of adsorption–desorption cycles within a limited time in the adsorption process. The other conclusion is that most of the MXene-based adsorbents have an unprecedented Hg(II) removal performance compared to other heavy metal ions, which makes them unique material for Hg(II) separation. However, on the other hand, there are contradicting observations especially about the effect of morphology and inadequate research on its efficiency in the industrial field and reusability. Instead of proven track records on the application of the MXene adsorbent for several continuous cycles, 11 cycles that were examined as a maximum in the literature are not enough to prove its regenerability. Therefore, we believe that heavy metal ion removal using MXene adsorbent requires a special consideration instead of the presence of published overarching studies.

## 7 Removal of Radionuclides

Considering the use of nuclear energy to a large extent, significant amounts of radionuclides are produced and released into nature as a nuclear disposal. Since radionuclides cause several disorders in human health and damage the environment, nuclear waste treatment with effective methods has become an important issue. Since the MXene family provides wide resistance to severe radiation, they are proposed as an effective adsorbent for removing radionuclides such as Ba(II),^321,325,331^ Cs(I),^332−334^ Eu(III),^273,335^ Re(VII),^336^ Sr(II),^321^ Th(IV),^336^ and U(VI)^126,254,335,337,338^ from nuclear waste as summarized in Table S6.

Uranium has both chemical and radiological toxicity, and its release to the environment is a serious concern. Therefore, several adsorbent materials including MXene have been widely explored for the removal of U(VI). The first study examining the removal of U(VI) from nuclear wastewater was carried out using the V_2_CT_*x*_ type of MXene.^126^ Maximum adsorption capacity of multilayered V_2_CT_*x*_ was measured as 174 mg/g. However, DFT calculation displayed that experimental measurements cannot reveal the actual performance of the adsorbent which was computed as 536 mg/g.^126^ Likewise, for the other type of MXene, Ti_3_C_2_(OH)_2_, DFT simulations also revealed high [UO_2_(H_2_O)_5_]^2+^ adsorption capacity of 595.3 mg/g.^126^ Therefore, further studies are aimed to improve the U(VI) adsorption performance of the MXene family either by intercalation, functionalization, or fabrication of composite adsorbents. For instance, it reached 214 mg/g by the intercalation of DMSO and NaOH^254^ and 344.8 mg/g by the functionalization with carboxyl groups for Ti_3_C_2_T_*x*_.^335^ Promisingly, U(VI) adsorption capacity hit the peak for Ti_2_CT_*x*_ as 470 mg/g, revealing the complete removal within 48 h.^337^ However, the functionalization with amidoxime resulted in a mild increase for a U(VI) uptake of 294 mg/g with a removal rate of 95% after 5 min.^338^ Perfect conductivity of MXene nanomaterials are provided as a benefit for the adsorption, and its electrochemical adsorption performance for U(VI) was tested under periodic alternating potential. Surprisingly, U(VI) adsorption capacity reached to 626 mg/g at pH 5 which was higher than the data observed from the conventional adsorption process and predicted from DFT simulations.^338^ This was the first study to obtain improved U(VI) adsorption using the electro-sorption process on functionalized MXene but hopefully will not be the last one. To further increase U(VI) adsorption capacity of MXene adsorbents, they were modified with several other materials. For instance, alkali-treated Ti_3_C_2_T_*x*_ was modified with nZVI, and resultant novel nanocomposite adsorbent unveiled the highest U(VI) adsorption capacity of 1246 mg/g.^339^ The great performance of Ti_3_C_2_T_*x*_/nZVI was attributed to the simultaneous formation of reduction precipitates and their adsorption from aqueous solution. Therefore, mainly to explain the adsorption mechanism of U(VI) on MXene, either a stable inner-sphere complex formation^335^ or a strategy of simultaneous adsorption and reduction^337,339^ was suggested.

Nuclear waste includes a high amount of Cs(I), a fission product of uranium.^340^ Therefore, its efficient removal using MXene-based adsorbents was also identified in the literature. However, a higher adsorption capacity of Cs(I) could not be reached as it was observed for U(VI) in MXene adsorbents. The removal rate of Ti_3_C_2_T_*x*_ fabricated via an in situ HF method was observed as 62.7% with a maximum adsorption capacity of 25.4 mg/g for Cs(I) within 1 min.^332^ Alternatively, Jun et al.^341^ reported the adsorption capacity of 148 mg/g using the same type of MXene for Cs(I) derived from nonradioactive CsNO_3_. To further increase Cs(I) adsorption performance of Ti_3_C_2_T_*x*_, it was fabricated as an aerogel sphere using sodium alginate.^334^ Encouraging results were observed as a maximum adsorption capacity of 315.91 mg/g for Cs(I).^334^ This was attributed to the mean contribution of trapping of Cs(I) in the aerogel sphere and ion-exchange reaction with either alginate or MXene.^334^

The final radionuclides for which the removal performance of MXene adsorbents was excessively investigated is Ba(II). While pristine Ti_3_C_2_T_*x*_ fabricated in different studies revealed almost the same Ba(II) adsorption capacity such as 9.3^325^ and 11.98^331^ mg/g, alkali treatment with 5% NaOH leads to three times the enhancement in the Ba(II) adsorption capacity of Ti_3_C_2_T_*x*_.^331^ With the increase in adsorbent dose, maximum Ba(II) uptake reached to 175.1 mg/g.^321^ It was proposed that inner-sphere complexation and ion-exchange mechanisms were responsible for the high performance of MXene, as given in Figure 8(b).

The reusability and regeneration property of MXene adsorbents are also investigated in the removal of radionuclides. Amidoxime-functionalized Ti_3_C_2_T_*x*_ kept its removal performance of U(VI) as nearly 100%, even after five adsorption–desorption cycles in 0.1 M Na_2_CO_3_ aqueous solution.^338^ Similar high regeneration performance was also proposed for DMSO-hydrated Ti_3_C_2_T_*x*_ used in U(VI) removal.^254^ Only 13% reduction in U(VI) uptake capacity was observed even after one month of the testing period. Furthermore, MXene treated with 0.2 M HNO_3_ after U(VI) saturation revealed the desorption efficiency of 98.5%, proving its excellent regenerability.^254^ In another study,^321^ slight decrements in removal rates of Ba(II) and Sr(II) as ∼5% and ∼8% were observed for pristine Ti_3_C_2_T_*x*_ after four cycles, respectively. However, there are few exceptions like the MXene/Ag_2_O_*x*_/PDA nanocomposite. Its recycle efficiency for I(I) decreased to nearly 50% of its initial I(I) removal capacity after the 5^th^ adsorption cycle.^342^

The critical issue in adsorbents is that they should not easily release the adsorbed ions, especially the radioactive ones to the environment. Therefore, leaching tests are performed by immersing the adsorbent into different aqueous media. It was also tested for some radionuclides trapped in MXene. For instance, Mu et al.^331^ immersed alkaline-treated Ti_3_C_2_T_*x*_ into the aqueous solutions for 3, 7, and 10 days to test the Ba(II) leaching. Ba(II) ion concentration in aqueous solutions was reported under the detection limit indicating the presence of irreversible interaction between MXene and Ba(II) at the adsorption conditions. Another study was carried out by Wang et al.^254^ where the U(VI) leaching test was performed for DMSO-hydrated Ti_3_C_2_T_*x*_ under various temperatures (200, 400, 450, and 500 °C) and atmospheres (air and N_2_). The optimum radionuclide imprisonment temperature was suggested as 400 °C in air for U(VI). Additionally, a long-term test of U(VI) leaching revealed that the leaching ratio was below 3% at the end of the 10 days. This study evidently proves the applicability of MXene nanomaterials for the encapsulation process.

To further evaluate the efficiency of MXene adsorbents, treatment of simulated or artificial wastewater-containing varying radioactive amounts was studied. The target was to display the potential of MXenes for industrial applications. For instance, different U(VI)-containing simulated water was tested for DMSO-hydrated Ti_3_C_2_T_*x*_, and almost 5000 kg of U(VI)-containing wastewater was treated using only 1 kg of adsorbent.^254^ After the treatment, it was proposed that the remaining U(VI) content in wastewater was under the standard of the World Health Organization (WHO) (0.015 mg/L). On the other hand, it was demonstrated that Ti_3_C_2_T_*x*_ had a superior potential to remove U(VI) with a removal rate of 95.7% in the presence of a high amount of several coexisting ions and under an anaerobic environment throughout many days.^337^ Removal efficiencies of the alkali-treated Ti_3_C_2_T_*x*_/nZVI nanocomposite adsorbent were observed as 88.9, 95.1, and 69.5% under various environmental media such as a 1.0 mM NaHCO_3_ aqueous solution, a solution mimicking groundwater, and a 10 mg/L humic acid aqueous solution, respectively.^339^ U(VI) removal performance of amidoxime-functionalized Ti_3_C_2_T_*x*_ was tested under simulated groundwater including U(VI), Ca(NO_3_)_2_, CaBr_2_, MgSO_4_, Na_2_SO_4_, NaHCO_3_, KHCO_3_, and Na_2_CO_3_ and compared to that of organic polyamidoxime (PAO).^338^ The adsorption capacity of amidoxime-functionalized Ti_3_C_2_T_*x*_ for U(VI) was around 230 mg/g while this value was 164 mg/g for PAO for a one day continuous measurement test.^338^ Similarly, Mu et al.^331^ tested the Ba(II) adsorption capacity of alkaline-treated Ti_3_C_2_T_*x*_ using simulated nuclear wastewater containing coexisting ions. The removal rate of MXene for Ba(II) was proposed as greater than 99%. Collectively, these studies proved that MXene are the favorable adsorbents for capturing radionuclides from actual nuclear wastewater.

MXene nanomaterials were only used as adsorbents rather than membranes for the removal of radionuclides utilizing the inner-sphere complex and ion-exchange mechanisms. Radionuclides of U(VI), Cs(I), and Ba(II) were highly investigated by MXene adsorbents, while the greatest performance was found for U(VI) removal. The most important feature of MXene nanomaterials demonstrated in the literature is that they effectively sequester radionuclides by hindering leaching. However, more research on the capacity of MXene adsorbents for removing radionuclides should be carried out, especially considering their well-proven ability of capturing radionuclides from actual nuclear wastewater.

## 8 Desalination

In parallel with the increase in industrialization and urbanizing population, a rise in water use is expected soon. Still, approximately 40% of the population on a global scale is affected by water scarcity, and unfortunately, this rate is expected to increase to 60% in 2025.^343^ Although there is a plenty of water in the world, only 2.5% of this water is available for utilization. Collectively, the increase in water usage and decrease in freshwater sources make the water treatment process as a key process in overcoming these problems, especially in water-stressed countries. Thermal-based processes such as evaporation, solar distillation, gas hydrate formation, etc. are the ones that have been used widely to remove the dissolved mineral salts in seawater.

### 8.1 Membrane-Based Separation

Membrane-based processes such as reverse osmosis (RO), nanofiltration, and electrodialysis are becoming more promising technologies because of their lower energy consumption, environmental footprint, and higher capacity. Desalination capacity via membrane-based separation techniques was around 97.5 × 10^6^ m^3^/day in 2015^344^ and, hopefully, was estimated to reach to 192 × 10^6^ m^3^/day by 2050.^345^ Industry that uses membrane-based desalination processes is dominated by polymeric membranes. However, novel advanced membrane materials for the desalination are emerging rapidly. Considering properties such as hydrophilicity, low contact angle, and easily functionalization, MXene nanomaterials are the most suitable candidates as the membrane material for the desalination processes. Table 4 summarizes the water permeance and salt rejection performances of existing MXene membranes. Ren et al.^125^ was the first group that introduced the MXene 2D nanomaterial for the desalination membranes. They synthesized a 1.5 μm-thick Ti_3_C_2_T_*x*_ membrane by vacuum-assisted filtration and obtained a water flux of 37.4 L/m^2^ × h × bar, which exceeded the performance of the industrial polymeric membranes.^125^ This unexpected performance was explained with the high water content of MXene layers in the wet state creating a free path for water flux. From permeation rates of ions such as Na^+^, Li^+^, K^+^, Ca^2+^, Mg^2+^, and Al^3+^, those of Na^+^, Li^+^, and K^+^ increased with the time due to the repulsive interaction between ions and MXene nanosheets arising from the formation of an electric double layer on the surface of the nanosheet due to the accumulation of salt ions. However, permeation rates of Ca^2+^, Mg^2+^, and Al^3+^ decreased due to attractive interactions between ions and highly negatively charged MXene nanosheets leading to their shrinkage. Simply, Ren et al.^125^ revealed the effect of radii and charge of ions on the water permeation for the first time where the existence of the ion-sieving mechanism for MXene membranes was displayed. Berdiyorov et al.^346^ carried out DFT calculations to provide an understanding about the size-charge selective ion sieving mechanism of MXene (Ti_3_C_2_) proposed by Ren et al.^125^ They suggested that this mechanism was interrelated with the electrostatic interactions between ions and the MXene surface, so the selectivity of ions originated from the surface charge network of MXene, which leads to a dynamic response to the penetrating ions by expanding or shrinking the interlayer spacing between the MXene nanosheets. Moving on the observations of two promising studies, a big door has been opened for researchers to create high separation performance membranes using MXene 2D nanomaterials. Generally, for the fast, reliable, and cost-effective observation, atomic-scale simulation approaches have been carried out to identify the effect of slit size and functional group of MXene, and the electric field was applied to define the desalination performance of MXene and, hence, to guide the experimentalists.^347−350^

**Table 4:** Survey of Desalination Performance of MXene-Based Membranes^a^

membranes	operation conditions (*P*: bar, *T*: °C)	ion type (concentration in g/L)	water (* pure) permeate (L/m^2^ × h × bar)	salt rejection (%)	ref
Ti_3_C_2_T_*x*_ on PVDF (1.5 μm)	–	AlCl_3_ (−)	18.0	–	(125)
Ti_3_C_2_T_*x*_ on PVDF (1.5 μm)	–	MgCl_2_ (−)	24.2	–	(125)
Ti_3_C_2_T_*x*_ on PVDF (1.5 μm)	–	NaCl (−)	45.7	–	(125)
Ti_3_C_2_T_*x*_ (Na^+^-intercalated) on PVDF (1.5 μm)	–	AlCl_3_ (−)	25.6	–	(125)
Ti_3_C_2_T_*x*_/GO (30 wt %) on PC (0.09 μm)	5, –	NaCl (5.85)	2.3	0	(193)
Ti_3_C_2_T_*x*_/GO (30 wt %) on PC (0.09 μm)	5, –	MgSO_4_ (24.65)	2.5	10	(193)
Ti_3_C_2_T_*x*_ on PES (0.065 μm)	2, –	Na_2_SO_4_ (1)	30*	97.5	(351)
Ti_3_C_2_T_*x*_ on PES (0.065 μm)	2, –	MgSO_4_ (1)	30*	95.4	(351)
Ti_3_C_2_T_*x*_ on PES (0.065 μm)	2, –	CaCl_2_ (1)	30*	52.0	(351)
Ti_3_C_2_T_*x*_ on PES (0.065 μm)	2, –	NaCl (1)	30*	20.0	(351)
Ti_3_C_2_T_*x*_ on PES	1, 20	NaCl (1)	435	13.8	(210)
Ti_3_C_2_T_*x*_ on PES	1, 20	Na_2_SO_4_ (1)	632	13.2	(210)
Ti_3_C_2_T_*x*_ on PES	1, 20	MgCl_2_ (1)	460	23	(210)
Ti_3_C_2_T_*x*_/CA (123 μm)	1, –	NaCl (2)	256	28	(220)
Ti_3_C_2_T_*x*_/CA (123 μm)	1, –	Na_2_SO_4_ (2)	256	59	(220)
Ti_3_C_2_T_*x*_/CA (123 μm)	1, –	MgSO_4_ (2)	256	56	(220)
Ti_3_C_2_T_*x*_/CA (123 μm)	1, –	MgCl_2_ (2)	256	40	(220)
Ti_3_C_2_T_*x*_/GO (30 wt %) (H_2_O_2_-treated) on MCE (0.262 μm)	1, 25	NaCl (0.29)	90	∼39	(228)
Ti_3_C_2_T_*x*_/GO (30 wt %) (H_2_O_2_-treated) on MCE (0.262 μm)	1, 25	Na_2_SO_4_ (0.71)	90	60.6	(228)
Ti_3_C_2_T_*x*_/GO (30 wt %) (H_2_O_2_-treated) on MCE (0.262 μm)	1, 25	MgSO_4_ (1.23)	90	∼31	(228)
Ti_3_C_2_T_*x*_/GO (30 wt %) (H_2_O_2_-treated) on MCE (0.262 μm)	1, 25	MgCl_2_ (0.48)	90	22.5	(228)
Ti_3_C_2_T_*x*_ on α-Al_2_O_3_ (0.1 μm)	3, 25	NaCl (0.59)	6.2	55.3	(354)
Ti_3_C_2_T_*x*_ on α-Al_2_O_3_ (0.1 μm)	3, 25	Na_2_SO_4_ (1.42)	3.5	75.9	(354)
Ti_3_C_2_T_*x*_ on α-Al_2_O_3_ (0.1 μm)	3, 25	MgSO_4_ (2.46)	4.7	67.3	(354)
Ti_3_C_2_T_*x*_ on α-Al_2_O_3_ (0.1 μm)	3, 25	MgCl_2_ (0.95)	8.5	46.1	(354)
Ti_3_C_2_T_*x*_ (Al^3+^-intercalated) on PES (0.34 μm)	OP^b^	NaCl (5.85)	8.5	89.5	(367)
Ti_3_C_2_T_*x*_ (Al^3+^-intercalated) on PES (0.58 μm)	OP^b^	NaCl (5.85)	4.8	92.3	(367)
Ti_3_C_2_T_*x*_/PA on PSF (0.205–0.375 μm)	16, 25	NaCl (2)	2.53	98.5	(352)
Ti_3_C_2_T_*x*_/SA (Mn^2+^-intercalated) on PVDF (0.05 μm)	1, –	Na_2_SO_4_ (0.05)	16.5	84	(357)
Ti_3_C_2_T_*x*_/SA (Mn^2+^-intercalated) on PVDF (0.07 μm)	1, –	Na_2_SO_4_ (0.05)	12.7	100	(357)
Ti_3_C_2_T_*x*_/Al_13_ on PVDF (∼0.05 μm)	OP^b^	NaCl (5.85)	0.30	99	(357)
Ti_3_C_2_T_*x*_ on PAN (∼0.06 μm)	0.004, 65	NaCl (35)	85.4	99.5	(382)
Ti_3_C_2_T_*x*_ on PAN (∼0.06 μm)	0.004, 30	NaCl (35)	48.2	99.5	(382)
Ti_3_C_2_T_*x*_ (MA-cross-linked) on nylon (∼0.03 μm)	0.0013, 30	NaCl (35)	22.8	99.9	(356)
Ti_3_C_2_T_*x*_ (MA-cross-linked) on nylon (∼0.03 μm)	0.0013, 30	KCl (35)	22.99	99.90	(356)
Ti_3_C_2_T_*x*_ (MA-cross-linked) on nylon (∼0.03 μm)	0.0013, 30	Na_2_SO_4_ (35)	21.97	99.98	(356)
Ti_3_C_2_T_*x*_ (MA-cross-linked) on nylon (∼0.03 μm)	0.0013, 30	MgSO_4_ (35)	20.89	99.99	(356)
Ti_3_C_2_T_*x*_ (MA-cross-linked) on nylon (∼0.03 μm)	0.0013, 30	MgCl_2_ (35)	21.35	99.97	(356)
Ti_3_C_2_T_*x*_/PVA/SSA composite on PTFE (0.23 μm)	0.0019, 70	KCl (44.73)	∼65^c^	99.81	(356)
Ti_3_C_2_T_*x*_/PVA/SSA composite on PTFE (0.23 μm)	0.0019, 70	Na_2_SO_4_ (85.22)	∼55.3^c^	99.91	(356)
Ti_3_C_2_T_*x*_/PVA/SSA composite on PTFE (0.23 μm)	0.0019, 70	MgSO_4_ (147.88)	∼55.4^c^	99.93	(356)
Ti_3_C_2_T_*x*_/PVA/SSA composite on PTFE (0.23 μm)	0.0019, 70	MgCl_2_ (57.13)	∼52.3^c^	99.93	(356)
Ti_3_C_2_T_*x*_/PVA/SSA composite on PTFE (0.23 μm)	0.0019, 70	CaCl_2_ (66.59)	∼54.2^c^	99.92	(356)
Ti_3_C_2_T_*x*_/P84 mixed matrix (150–20 μm)	1, 20	Na_2_SO_4_ (1)	423	0	(222)
Ti_3_C_2_T_*x*_/P84 mixed matrix (150–20 μm)	1, 20	MgCl_2_ (1)	418	0	(222)
Ti_3_C_2_T_*x*_ (casted with EPD) (0.2 μm)	OP^b^	NaCl (11.7)	–	∼99.6	(359)
Ti_3_C_2_T_*x*_ (casted with EPD) (0.2 μm)	OP^b^	MgCl_2_ (19.04)	–	∼99.6	(359)
Ti_3_C_2_T_*x*_ (casted with EPD) (0.2 μm)	OP^b^	AlCl_3_ (26.67)	–	∼99.6	(359)
Ti_3_C_2_T_*x*_ on PES (0.2 μm)	OP^b^	NaCl (11.7)	–	∼94.8	(359)
Ti_3_C_2_T_*x*_ on PES (0.2 μm)	OP^b^	MgCl_2_ (19.04)	–	∼96.5	(359)
Ti_3_C_2_T_*x*_ on PES (0.2 μm)	OP^b^	AlCl_3_ (26.67)	–	∼98.2	(359)
Ti_3_C_2_T_*x*_/PA on PSF	3, 25	NaCl (2)	3.1	13.84	(358)
Ti_3_C_2_T_*x*_/PA on PSF	3, 25	Na_2_SO_4_ (2)	4.7	97	(358)
Ti_3_C_2_T_*x*_/PA on PSF	3, 25	MgSO_4_ (2)	4.9	92.35	(358)
Ti_3_C_2_T_*x*_/PA on PSF	3, 25	MgCl_2_ (2)	3.2	48.36	(358)
Ti_3_C_2_T_*x*_/PAN hollow fiber (∼0.09 μm)	1, 25	Na_2_SO_4_ (0.05)	∼5.9	∼70	(383)
Ti_3_C_2_T_*x*_/PAN hollow fiber (∼0.09 μm)	1, 25	MgSO_4_ (0.05)	∼6.0	∼45	(383)
Ti_3_C_2_T_*x*_/PAN hollow fiber (∼0.09 μm)	1, 25	MgCl_2_ (0.05)	∼6.9	∼36	(383)
Ti_3_C_2_T_*x*_/PAN hollow fiber (∼0.09 μm)	1, 25	NaCl (0.05)	∼6.2	∼28	(383)
Ti_3_C_2_T_*x*_ (5 mg)/PA on PES	OP^b^	NaCl (0.23)	13.5	–	(362)
Ti_3_C_2_T_*x*_ (15 mg)/PA on PES	OP^b^	NaCl (0.23)	15.2	–	(362)
Ti_3_C_2_T_*x*_ (25 mg)/PA on PES	OP^b^	NaCl (0.23)	14.1	–	(362)
Ti_3_C_2_T_*x*_ (35 mg)/PA on PES	OP^b^	NaCl (0.23)	14.0	–	(362)
Ti_3_C_2_T_*x*_/PA (immersed in organic phase) on PSF	4, 25	Na_2_SO_4_ (2)	2.33	98.6	(360)
Ti_3_C_2_T_*x*_/PA (immersed in aqueous phase) on PSF	4, 25	Na_2_SO_4_ (2)	4.70	97.6	(360)
Ti_3_C_2_T_*x*_ (modified with PFDTMS) on PVDF (120 μm)	–, 20	seawater	1.88^c^	100	(371)
Ti_3_C_2_T_*x*_ (modified with HTEOS) on PVDF (120 μm)	–, 20	seawater	0.85^c^	100	(371)
Ti_3_C_2_T_*x*_/PA on PDA	OP^b^	NaCl (58.5)	27.5	–	(384)
Ti_3_C_2_T_*x*_/CNT/PA on PDA	OP^b^	NaCl (58.5)	31.5	–	(384)

a Membrane thickness is given in parentheses. CA: cellulose acetate, CNT: carbon nanotube, EPD: electrophoretic deposition, GO: graphene oxide, HTEOS: hexadecyltrimethoxy silane, MA: maleic acid, MCE: mixed cellulose ester, PA: polyamide, PAN: polyacrylonitrile, PDA: polydopamine, PES: polyethersulfone, PFDTMS: 1H,1H,2H,2H-hepta decafluoro decyltrimethoxy silane, PSF: polysulfone, PTFE: polytetrafluoroethylene, PVA: poly (vinyl alcohol), PVDF: polyvinylidene difluoride, P84: commercial copolyimide, SA: sodium alginate, SSA: sulfosuccinic acid.

b Osmotic pressure (OP) corresponding to 2 M sucrose.

c Unit of permeate in kg/m^2^ × h.

**Figure 9:**
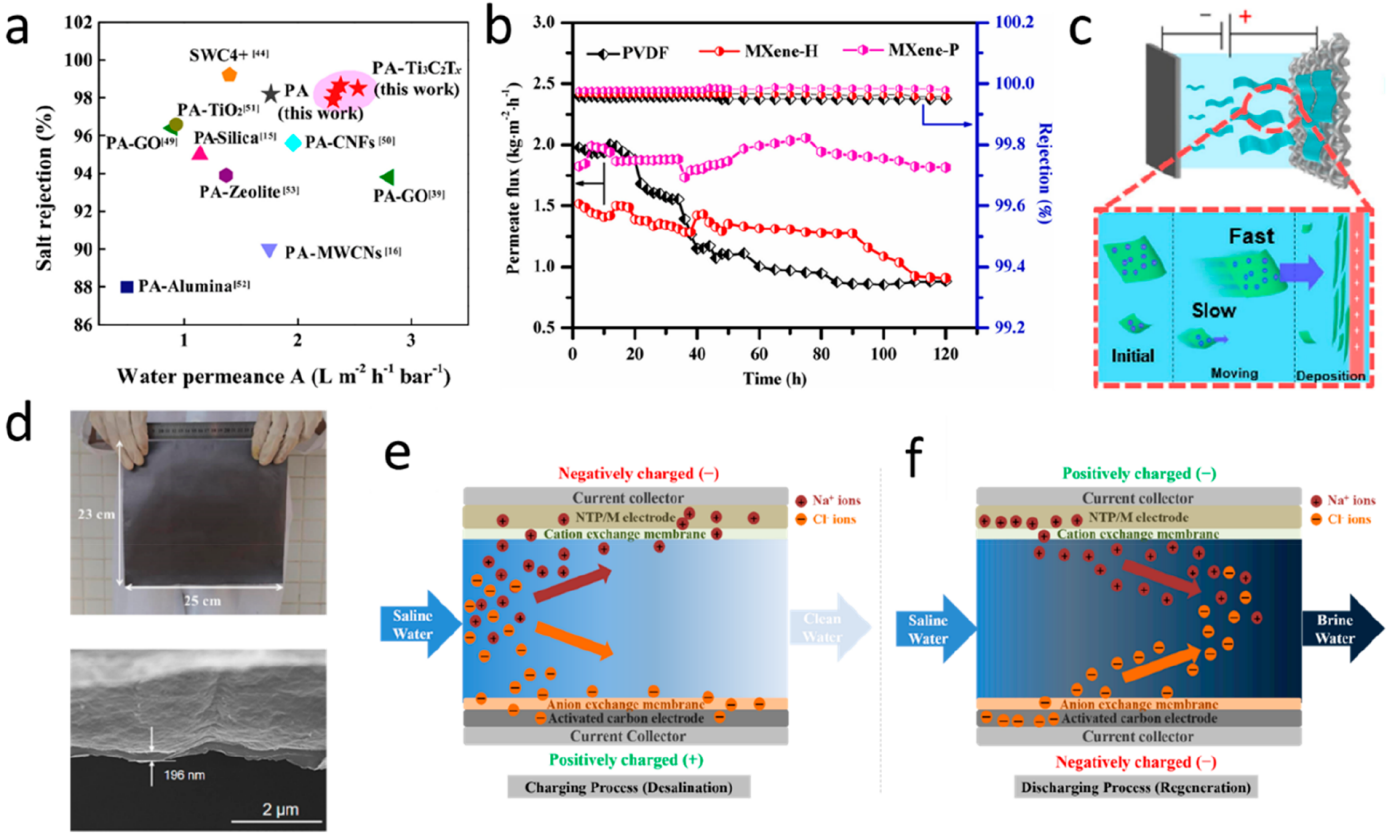
Desalination performance of MXene membranes. (a) Comparison of water permeability and salt rejection for the polyamide-based nanocomposite RO membranes (NaCl: 2 g/L, water pressure: 16 bar, temperature: 25 °C). Adapted with permission from ref (352). Copyright 2020 Elsevier. (b) Variation of permeate flux and salt rejection as a function of time (MXene-H and -P represent the hexadecyl trimethoxysilane (HTEOS) and 1H,1H,2H,2H-heptadecafluorodecyl trimethoxysilane (PFDTMS) grafted MXene, respectively). Adapted with permission from ref (371). Copyright 2022 Elsevier. (c) Illustration of the electrophoresis deposition process for the preparation of MXene membranes. Adapted with permission from ref (359). Copyright 2021 Elsevier. (d) Digital photo of the EPD-MXene membrane having a large area (top) and SEM images of the cross-section of the EPD-MXene membrane (bottom). Adapted with permission from ref (359). Copyright 2021 Elsevier. Schematic illustration for hybrid capacitive deionization process of (e) the charging (desalination) and (f) discharging (regeneration) steps. Adapted with permission from ref (381). Copyright 2021 Elsevier.

Membrane technologies focused on designing highly selective nanofiltration, RO, and promising forward osmosis (FO) MXene-based membranes, which can selectively separate salt ions by either size- or charge-exclusion. Initially, TFN membranes were engineered by the deposition of MXene (Ti_3_C_2_T_*x*_) nanosheets on the PES support via vacuum filtration and then an interfacial polymerization reaction was carried out to test the nanofiltration performance for divalent salt ions. For example, Xu et al.^351^ reported 97 and 95.3% rejection rates of this TFN membrane for Na_2_SO_4_ and MgSO_4_ with 45.7 L/m^2^ × h × bar of water permeance where it exhibited a selective separation factor (α(NaCl/MgSO_4_) of 14.5. To further enhance the performance of the MXene TFN membrane, MXene nanosheets were dispersed in aqueous solution during in situ interfacial polymerization and higher selectivity of the monovalent salt ions were achieved for RO technology. Wang et al.^352^ achieved the highest NaCl rejection rate of 98.5% and water flux of 2.5 L/m^2^ × h × bar compared to the other TFN membranes [see Figure 9(a)]. In addition to the experimental measurements, molecular simulation approaches support the optimal performance of MXene membranes for the RO process. Meidani et al.^353^ calculated the water and salt ion transport through the pore of three types of MXene (Ti_2_C, Ti_3_C_2_, and Ti_4_C_3_), graphene, and MoS_2_ membranes. Permeability coefficients were reported in the order of Ti_3_C_2_ > Ti_2_C > Mo_2_S > Ti_4_C_3_ > graphene under low pressure (<100 bar), which is generally applied in the simulation approaches, showing the applicability of MXene membranes for the desalination process.^353^ The Ti_3_C_2_ membrane exhibited the highest permeation rate of 11.4 L/cm^2^ × day × MPa (475 L/m^2^ × h × bar) with an ion rejection rate of 100% more than the rest of the investigated membranes.

GO is widely studied and accepted as the next-generation material in membrane technology due to its chemical resistance, mechanical robustness, and selective separation of ions. Although the fabrication of the nanocomposite membrane by combining GO and MXene has been suggested by researchers as another strategy to design high-performance membranes for desalination, satisfactory results have not been achieved yet. For instance, Ti_3_C_2_T_*x*_/GO nanocomposite membranes were prepared by mixing different GO concentrations (10–30 wt %) with Ti_3_C_2_T_*x*_ by Kang et al.^193^ Rejection rates of NaCl and MgSO_4_ were below 11% with the permeances of 2.25 and 2.35 L/m^2^ × h × bar, respectively, due to the swelling where the optimum interlayer spacing was determined around 5 Å for the nanocomposite membrane. Likewise, Han et al.^228^ fabricated Ti_3_C_2_T_*x*_/GO nanocomposite membranes with various MXene amounts and then applied H_2_O_2_ to create TiO_2_ nanocrystals via in situ oxidation. Pure water permeability of nanocomposite membranes reached from ∼31.34 to 108.7 L/m^2^ × h × bar with an increase in the MXene amount from 10 to 40%. However, salt rejection rates for Na_2_SO_4_, NaCl, MgSO_4_, and MgCl_2_ were still low as 60.6, ∼39, ∼31, and 22.5%, respectively. Nevertheless, in order to obtain higher separation efficiency in the desalination process, more research on MXene/GO nanocomposite membranes should be carried out.

To improve the desalination performance of MXene-based membranes, one of the efficient strategies is adjusting the interlayer spacing of nanosheets. Sun et al.^354^ fabricated MXene-based membranes with 0.1 μm thickness on α-Al_2_O_3_ tubular supports and used a sintering-temperature regulation method to design the interlayer distance that selectively separated ions. The interlayer distance decreased from 3.71 to 2.68 Å with the increase in temperature from 60 to 400 °C due to the change in functional group concentration on the surface of MXene, which results in the cross-linking via thermal treatment. The pure water permeance of membrane treated at 400 °C was reported as 11.5 L/m^2^ × h × bar, and the improvement in rejection rates of the MXene membrane with the rise in treatment temperature from 200 to 400 °C was obtained as 32.3, 31.4, 34, and 43.3% for Na_2_SO_4_, MgSO_4_, NaCl, and MgCl_2_, respectively. Using a similar approach as the thermal treatment at 180 °C, Lu et al.^355^ confined the interlayer spacing of MXene via a self-cross-linking strategy from 13.61 to 12.87 Å in the dry state and from 16.68 to 15.54 Å in the wet state. Thanks to the self-cross-linking mechanism of MXene, the permeation rate of the monovalent ions such as K^+^, Na^+^, and Li^+^ reduced from the order of 10^–1^ to 10^–3^ mol/m^2^ × h and a comprehensive increase was observed in the NaCl rejection rate, reaching to 98.6%. They attributed the interlayer distance shrinkage to the thermal treatment induced by the self-cross-linking behavior. With the increase in temperature, bonded water and −(OH)_2_ groups on the surface of MXene nanosheets were decreased and then Ti–O–Ti bonds (each Ti from neighboring sheets) were formed leading to the self-cross-linked MXene and confined interlayer spacing. As a result, the swelling of 2D membranes and poor ion rejection were overcome.

Swelling is considered as a big challenge for 2D membranes, since it leads to a low rejection rate of small ions during desalination. With the exposure of membrane to water, they are usually swelled resulting in the enhanced *d*-spacing, decreased stability, and poor ion-sieving. Although MXene nanomaterials are being used in the membrane separation technology more day by day because of their outstanding properties, their hydrophilic nature was proposed as the prospective reason for swelling. Several strategies have been applied to prevent enhancement of the interlayer space between nanosheets such as self-cross-linking,^354,355^ chemical cross-linking,^356^ and cation intercalation.^357^ Ding et al.^356^ used maleic acid for the chemical cross-linking of Ti_3_C_2_T_*x*_ nanosheets, which led to the change in *d*-spacing as 12.5% in contact with water. However, the *d*-space of pristine Ti_3_C_2_T_*x*_ increased 149% in the wet state. Chemical cross-linking between hydroxyl groups of MXene and carboxyl groups in maleic acid prevented the membrane from swelling. A covalently bridged Ti_3_C_2_T_*x*_ membrane revealed high salt rejection of >99.9% in different salt solutions (3.5 wt %) as KCl, MgCl_2_, Na_2_SO_4_, and MgSO_4_ and water flux ranged between 20 and 23 kg/m^2^ × h. In another study, to overcome the swelling of the PVA pervaporation desalination membrane, MXene (Ti_3_C_2_T_*x*_) nanosheets were inserted and PVA was cross-linked with sulfosuccinic acid.^356^ The swelling degree of this composite membrane (48%) was lower than the pure PVA membrane (283%). This severe hindrance in swelling was attributed to the mutual effect of the formation of an ester linkage between PVA and SFA, and hydrogen bonds between sulfonic/ester groups and functional groups on the surface of MXene, possibly occurring during annealing. The water flux of the composite membrane was 62.2 kg/m^2^ × h with a NaCl rejection rate of 99.8% and was kept constant during a 50 h desalination process at 30 °C. Wang et al.^357^ suggested an efficient combined strategy using SA and various cations such as Ca^2+^, Ba^2+^, and Mn^2+^ together, where the former has hydrogen bonding ability with the MXene layer and the latter can electrostatically interact with the former. Unfortunately, with the immersion of pristine MXene in various salty solutions including NaCl, LiCl, RbCl, MgCl_2_, CaCl_2_, AlCl_3_, and NH_4_Cl, its interlayer spacing difference was reported as 1.79 Å between the solutions leading to the maximum and minimum interlayer distance. However, it was only 0.52 Å for the MXene membrane anchored with SA on the nanosheet surface and intercalated with Ca(II). Similar swelling resistance performance was observed for MXene membranes intercalated with Ba^2+^ and Mn^2+^ with an interlayer spacing constrained at 16.2 ± 0.2 Å. As a result, an improved separation performance was achieved, although ion-sieving mechanisms changed due to the affinity of SA molecules to the cations. For instance, the MXene membrane anchored with SA on the nanosheet surface and intercalated with Mn^2+^ having thicknesses of 0.05 and 0.08 μm showed high permeances of 16.5 and 12.7 L/m^2^ × h × bar and Na_2_SO_4_ rejection rates of 84 and 100%, respectively. The other study that proves the success of intercalation of the cation within MXene nanosheets was carried out by Zhu et al.^357^ They fabricated a Keggin Al_13_ polycation intercalated Ti_3_C_2_T_*x*_ membrane via vacuum filtration and aimed to utilize from the electrostatic interaction between the Keggin Al_13_ ion (zeta potential: +24 mV) and Ti_3_C_2_T_*x*_ (zeta potential: −31.3 mV). The pristine Ti_3_C_2_T_*x*_ membrane swelled after soaking into the various salt solutions such as LiCl, NaCl, KCl, MgCl_2_, CaCl_2_, and AlCl_3_ for 1 h, and its *d*-spacing varied between 14.7 and 22.1 Å (difference: 7.4 Å). However, the Keggin Al_13_ ion intercalated Ti_3_C_2_T_*x*_ membrane showed little variance for *d*-spacing as 0.3 Å with nearly suppressed value at around 11.5 Å even at wet or dry conditions. Suppressed swelling phenomena led to the NaCl rejection of 99% under osmotic pressure (2 M sucrose). Therefore, the proposed antiswelling strategies are encouraging for the desalination MXene membranes in adjusting the interlayer spacing with angstrom precision and, hence, improving the salt rejection.

Maintaining ion-sieving performance of membranes as long as possible during and after long consecutive operation cycles is the other challenge in desalination. Research to test the long-term structural and performance-based stability of membranes is emerging. Therefore, either long-term measurements^355,356,358^ or long time water immersion tests^357,359,360^ are carried out in order to provide knowledge about the stability. The longest desalination test was carried out about 58 days for Ti_3_C_2_T_*x*_-based TFN membranes without the decrease in salt rejection.^358^ The other study carried out by Lu et al.^355^ displayed the effect of self-cross-linking via thermal treatment on the long permeation measurements as >70 h. Additionally, self-cross-linked MXene displayed structural stability up to 12 h after immersion in solutions at different pH values such as 1.5, 7.0, and 11.3. Although not as long as the permeation tests of Lu et al.,^355^ Yang et al.^356^ also achieved stable water permeation for the MXene-based nanocomposite membrane for 50 h. Wang et al.^357^ soaked the MXene membrane, prepared by anchoring SA on a nanosheet surface and intercalating Ca^2+^ ions, in water for 20 days and observed almost the same NaCl permeation rate of a fresh MXene membrane. On the other hand, at the end of the third permeation/drying cycles, its NaCl permeation rate still did not change, proving its long-term usability. Likewise, different salt solutions such as NaCl, MgCl_2_, KCl, and AlCl_3_ were applied for the consecutive cycles lasting 4 h, and ion permeation rates did not change significantly, indicating its superb regeneration ability. The longest immersion was carried out by Xue et al.,^360^ where the MXene-based TFN membrane was immersed in water for 105 days with no change in Na_2_SO_4_ rejection and slight decrease in flux.

Performance-based stability of membranes in desalination is mainly hindered by fouling phenomena, which is the blockage of membrane pores by foulants such as proteins, lipids, bacteria, etc., and their accumulation on the membrane surface, leading to the reduction in efficiency of process and increase in cost due to the requirement of greater pressure. Three types of fouling are observed: organic fouling, deposition of salts, and biofouling. There are many factors affecting the membrane fouling such as water characteristics, process conditions, and most importantly properties of the membrane itself. Thus, to develop a membrane with low fouling tendency is so important for the effective desalination performance. Generally, BSA solution is used to test the fouling behavior of membranes. For instance, Shen et al.^361^ reported that the flux recovery ratio of the Ti_3_C_2_T_*x*_ membrane on a polysulfone (PSF) support was 76.1%, while it was only 48.3% for the pristine PSF membrane after physical cleaning, indicating improved antifouling properties of MXene membranes. Another study that used BSA to test the membrane fouling was done by Pandey et al.^220^ The Ti_3_C_2_T_*x*_/CA composite membrane cross-linked with formaldehyde exhibited 100% rejection rate for BSA, whereas the pristine CA membrane showed a lower rejection rate of 65.3%.^220^ An improvement of 35% for the BSA rejection was explained as the formation of small pores and reduction in macrovoids via addition of MXene into the CA matrix and chemically cross-linking. Encouragingly, Xu et al.^362^ fabricated a novel membrane where MXene nanoplatelets were deposited on the outer surface of PES via vacuum filtering and selective polyamide (PA) layer was synthesized by the interfacial polymerization on the other side of PES. This novel layered membrane revealed the decrement in water flux of only 8.2% and 4.5%, respectively, without and with an applied electric field of +2 V.^362^ On the other hand, Tan et al.^130^ fabricated MXene (Ti_3_C_2_T_*x*_) as a coating material via vacuum filtration onto the PVDF support membrane to mitigate fouling in a DCMD process. They observed a water flux decrement in the absence and presence of light irradiation as only 8.3 and 6.6% for the MXene-coated membrane, whereas those of 18.8 and 18.2% were reported for the PVDF membrane, respectively, after 21 h of continuous filtration process.^130^ It was ascribed to its repulsive interaction with BSA and its ability to accommodate BSA within its intercalated structure.

Similar as fouling, performance stability of a membrane is also influenced by biofouling, which is a complex, reversible, or irreversible adhesion of microorganisms on a membrane surface and their growth within a couple of weeks or months. Biofouling has severe adverse effects on membrane processes such as flux decline, membrane biodegradation, rise in energy consumption, and decrease in salt rejection. Although biofouling has been known as a contributing factor to more than 45% of all membrane fouling,^363^ limited studies have investigated the biofouling resistance of MXene membranes. Pandey et al.^46,220^ showed the antibacterial capacity of Ti_3_C_2_T_*x*_ and two niobium-based carbide MXenes (Nb_2_CT_*x*_ and Nb_4_C_3_T_*x*_). They reported the growth inhibition of bacteria of almost >98% and 96% for *E. coli* (Gram-negative bacteria) and *B. subtilis* (Gram-positive bacteria) for the membrane consisting of 10% MXene content, respectively.^220^ Similarly, high growth inhibitions of Nb_2_CT_*x*_ and Nb_4_C_3_T_*x*_ were recorded as 94.2 and 96.1% for *E. coli* and 91.6 and 93.7% for *S. aureus* within 3 h of incubation, respectively.^46^ Pandey and colleagues attributed this exceptional antibacterial activity of MXenes to their nanoknife behavior toward the bacteria by the help of their sharp edges. However, instead of fabricating MXene-based composite membranes, Zha et al.^364^ suggested that coating Ti_3_C_2_T_*x*_ MXene nanomaterials on the surface of a cellulose membrane was more efficient. Cell viability was much more hindered, and the highest antibacterial efficiency of 99.99 and 99.98% for *E. coli* and *S. aureus* were observed after 24 h of contact time, respectively.^364^ Collectively, not only the MXene type but also the membrane preparation procedure has significant effects on cell viability. In addition to the studies of the group of Pandey,^46,220^ Jastrzȩbska et al.^365^ identified the biological activity of different types of MXene nanomaterials as Ti_3_C_2_T_*x*_ and Ti_2_CT_*x*_ for *E. coli*. They concluded that atomic structure with specific stoichiometry is crucial for the bioactivity of MXenes rather than the chemical composition. Antibacterial capacity of specific types of MXene was elaborated in the review of Seidi et al. in depth.^366^ However, there are more than hundreds of stoichiometric MXene compositions (at least) and a countless number of solid solutions. Therefore, more research needs to be done to determine complete antibacterial capacity of the MXene family.

The chlorination process is mostly used in water remediation to reduce membrane biofouling. However, chlorine can damage the membrane and reduce its separation performance. Therefore, producing a chlorine-resistant membrane is the key point for the desalination process. Ding et al.^367^ investigated the chlorine resistance of pristine and Al^3+^-intercalated Ti_3_C_2_T_*x*_ membranes by treating them with 200 mg/L NaClO for 24 h. While Na^+^ permeation rates of pristine MXene were increased almost 5-fold, those of the Al^3+^-intercalated MXene membrane were only doubled after treatment.^367^ Moreover, Al^3+^-intercalated MXene membranes showed high and nearly stable Na^+^ rejection rates of 98% after chlorine treatment, whereas it was below 40% for the pristine MXene membrane. Harsher conditions were also tested for defining the chlorine resistance of MXene membranes. For instance, Wang et al.^352^ applied 5 chlorine cleaning cycles using 2000 mg/L NaClO solution to test the Ti_3_C_2_T_*x*_-based TFN membrane. Almost stable water flux and salt rejection of >97% were reported for the TFN membrane, whereas the water flux of the PA membrane increased 3.5-fold along with the decrease in salt rejection rate to 94% during 5 chlorine cleaning cycles. Most importantly, the TFN membrane with a constant salt rejection rate of 97.1% was unveiled after the chlorination test of 10,000 mg/L × hour NaClO, proving its excellent chlorine resistance property.^352^ The reason for the excellent chlorine resistance of the TFN membrane was attributed to the interaction between the surface functional groups of the MXene nanosheets and chlorine, which was utilized to prevent the chlorine attack on the PA matrix.

Seawater contains salts, organic compounds, minerals, and microbial organisms, which influence the selective separation of membranes. The most feasible way to reveal the accurate performance of membranes in a lab or pilot scale is to investigate their performance for artificial/simulated water, which mimics real seawater. Zhao et al.^368^ tested the solar desalination performance of the Ti_3_C_2_T_*x*_ membrane from the simulated water containing RhB and MO dyes and Cu^2+^ and Cr^6+^ ions and achieved high salt rejection rates varied in the range of 99.6–100%. Likewise, the MXene membrane displayed a decrease in salt ion concentrations below ∼10 mg/L, which is suitable for the drinking water standards of WHO, from Bohai seawater including Na^+^, K^+^, Mg^2+^, and Ca^2+^ ions.^368^ Similarly, for the separation of various real seawater samples with the different salt concentrations (Yellow sea: 31‰, Bohai sea: 30‰, Alkali lake 1–2:12–76‰), Zhang et al.^369^ tested the vertically aligned Janus MXene (Ti_3_C_2_T_*x*_) aerogel. The salinity ratio decreased under the drinking water standards of WHO (1‰) and EPA (0.5‰) after performing solar desalination tests using the MXene aerogel. Moreover, Wang et al.^370^ reached the ion concentration ranging between 0.16 and 1.12 mg/L for Na^+^, K^+^, Mg^2+^, and Ca^2+^ ions in the purified water using five-layered cotton fabrics coated with MXene and CNT. Zhang et al.^371^ suggested that the silane-modified MXene membrane was stable during the 120 h treatment of actual seawater, as illustrated in Figure 9(b). The encouraging desalination performance of MXene-based membranes from both real and simulated seawater proves that the MXene nanomaterial can be used safely in membrane technology.

The main desire is for a novel membrane material to find a place in the membrane market. Therefore, scientists and/or engineers should concentrate on its scalability, which is the most important challenge in the membrane production step. Wu et al.^372^ proposed a facile and novel method to create a Ti_3_C_2_T_*x*_ interlayered polyamide membrane having a large effective membrane area. This method consists of brush coating of the MXene solution and then interfacial polymerization. The MXene TFN membrane with an area of 24 × 8 cm^2^ exhibited low salt fluxes of 0.07 and 0.27 g/L and high-water fluxes of 15.6 and 23.9 L/m^2^ × h in the active layer faces to the feed and draw solutions, respectively.^372^ In a different way, Deng et al.^359^ prepared the Ti_3_C_2_T_*x*_ membrane with a large area of 575 cm^2^ via EPD within only 10 min. Rejection rates of Na^+^, Mg^2+^, and Al^3+^ reached to 99.6% for the membranes fabricated via EPD compared to the MXene membranes synthesized via traditional vacuum filtration with a salt rejection of 94.7%. It was revealed that EPD led to the deposition of big MXene nanosheets onto the support, which was called “smart selection” [see Figure 9(c)], resulting in a more ordered MXene membrane, as given in Figure 9(d). This method seems as scalable as brush coating and interfacial polymerization, but unfortunately it is cost-intensive. Unfortunately, more research still needs to be carried out in the membrane engineering on the design of both scalable and easy production methods to fabricate MXene membranes with a high desalination performance.

MXene-based membranes were used in nanofiltration, RO, and FO processes for the separation of salt ions by either size- or charge-exclusion. With the help of electrostatic interactions between ions and the MXene surface, the expansion or shrinkage of interlayer distance was directed by the ion-sieving mechanism successfully in the desalination process. Either to enhance their desalination performance or inhibit the swelling phenomena, the main strategy followed was adjusting the interlayer spacing of the nanosheets by self-cross-linking via thermal treatment,^354,355^ chemical cross-linking,^356,373^ and cation intercalation.^357,374^ Considerable studies were carried out for identification of swelling, long-term stability, and fouling behavior of MXene nanomaterials with great success. Especially, we believe that the stability of the MXene membrane during the desalination process is well-documented, reaching up to 105 days with no alteration in Na_2_SO_4_ rejection. Since antifouling, antibiofouling, and chlorination resistance of a specific type of MXene were revealed, other members of this fastest growing 2D inorganic nanomaterial family should be identified as well. The most promising and thrilling observation for MXene-based membranes in desalination is their scalability, which will enable its usage in the near future in industrial application.

### 8.2 Solar Desalination

Alternative energy sources have gained vital importance these days, where the limit of global warming is expected reach to above 1.9 °C by 2100.^375^ Therefore, the use of plentiful sunlight in the desalination process has garnered increasing attention due to its environmentally friendly and low-cost technology. Considering the capacity of MXene to convert the light efficiently to heat, it would not be a surprise to encounter the usage of the MXene family for the solar desalination process.^364,368−370,376−379^ Zhao et al.^368^ used a Ti_3_C_2_T_*x*_ membrane, which was modified with trimethoxy (1H,1H,2H,2H-per-fluorodecyl) silane (PFDTMS), in solar desalination for the first time. The solar evaporation rate of the MXene membrane was reported as 1.31 kg/m^2^ × h with a solar steam conversion efficiency of 71% under 1 sun. Additionally, its solar steam generation performance was almost constant for 10 cycles under 1 sun, proving its excellent durability. Salts were accumulated under the membrane, and no deterioration was observed onto the upper surface of the membrane. For the MXene/cellulose composite membrane, both higher steam conversion efficiency (85.8%) and water evaporation rate (1.44 kg/m^2^ × h) were observed by Zha et al.^364^ than the data provided by Zhao et al.^368^ Similar results (conversion efficiency of 87%, and water evaporation rate of 1.46 kg/m^2^ × h under 1 sun for 15 days) were displayed by Zhang et al.^369^ for the vertically aligned Janus Ti_3_C_2_T_*x*_ aerogel modified with the fluorinated alkyl silane. Solar-assisted water evaporation technology is also used for the textile water purification, in addition to the seawater desalination. Wang et al.^370^ designed cotton fabrics by coating first with Ti_3_C_2_T_*x*_ and then with CNT in a manner of layer-by-layer assembly. Evaporation rates of distilled and textile wastewater were proposed as 1.35 and >1.16 kg/m^2^ × h, respectively, with high efficiency of 88.2% for five-layered cotton fabrics under 1 sun. The other nanocomposite membrane was fabricated by Ming et al.^376^ for the same purpose by combining GO and Ti_3_C_2_T_*x*_ using SA as a binder. The evaporation rate of this aerogel increased to ∼1.27 kg/m^2^ × h within only 10 min with an evaporation efficiency up to 90.7% under 1 sun. Moreover, its evaporation rate and efficiency enhanced to 6.70 kg/m^2^ × h and ∼93.9% under 5 suns. Collectively, these research studies suggested that MXene-based membranes have a great potential for water remediation via solar driven membrane-based desalination processes.

### 8.3 Capacitive Deionization

The capacitive deionization (CDI) is an alternative system for the desalination where dissolved ions were separated from water by electrodes via applying a small electrical voltage, as illustrated in Figure 9(e,f). The efficiency of this electrochemical separation process is hindered by the selection of electrode material. MXene nanomaterials are also promising electrode materials for CDI due to their high electrical conductivity, hydrophilicity, booklike structure, and tunable surface chemistry. Ma et al.^380^ investigated the desalination capacity of Ti_3_C_2_T_*x*_ films having less fluorine terminal groups. Average desalination capacity of MXene was observed as 68 mg/g with the initial NaCl concentration of 585 mg/L at a current density of 20 mA/g and voltage window of 1.2 V for 3 cycles. Similar performance (desalination capacity of 72 mg/g) but higher durability was observed by Shen et al.^380^ for the hybrid MXene electrode produced by the intercalation of MXene nanosheets with small size into the larger ones by vacuum filtration. The desalination capacity was kept constant even after 50 cycles at 1.4 V with a 5 mM NaCl initial concentration. The best desalination performance was obtained for the Ti_3_C_2_T_*x*_/Na_2_Ti_2_(PO_4_)_3_ nanohybrid electrode designed by Chen et al.^381^ Desalination performance of the nanohybrid electrode increased from 32.3 to 128.6 mg/g at the operation voltage of 1.8 V with a NaCl concentration from 250 to 1000 mg/L. In addition, the desalination capacity was maintained above 100 mg/g without significant impairment for 20 cycles. This success was related to the cooperation of MXene, which increases electrical conductivity and provides significant interlayer spacing for ion transport. As a result of these outstanding data, MXene will increasingly take place in CDI as an electrode. The list of other promising studies that incorporate MXene in the CDI process are listed in Table S7.

## 9 Other Prominent Applications

Since the discovery of the MXene family in 2011 by Gogotsi and co-workers, it has become one of the fastest growing family of 2D inorganic materials. To date, approximately 150 MAX phases^20^ have been explored experimentally and dozens of MXene forms have been revealed by the computational approaches. Due to their attractive properties such as metallic conductivity, elastic mechanical strength, hydrophilicity, surface chemistry, and interlayer engineering properties, the MXene family has been dominantly tested for the different emerging areas such as the removal of toxic substances^131−133^ and pharmaceuticals^134−136,385−387^ from water as well as biomedical^138−141^ and controlled release^142^ applications.

Phosphate and nitrate ions are soluble, toxic, and carcinogenic substances for living organisms. When their amount increased in water, methemoglobinemia and eutrophication threaten human health and water safety directly. MXene nanomaterials were also evaluated for the separation of these substances. The first study reported by Zhang et al.^132^ investigated the phosphate adsorption performance of a sandwich-like layered Ti_3_C_2_OH_0.8_F_1.2_/Fe_3_O_4_ nanocomposite. Thanks to the Fe_3_O_4_ molecules coordinated on the MXene surface, adsorption of the phosphate ion significantly increased within the first 20 min. The MXene/Fe_3_O_4_ nanocomposite composed of a weight ratio of 2/1 displayed a phosphate adsorption of 9.42 mg/g from an initial ion concentration of 10 mg/L with the removal efficiency of ∼87% at pH ∼6.^132^ Importantly, the treatment capacity was 2100 kg per water with 2 mg/L phosphate and 200 mg/L nitrate in the effluent (sewage discharge standard) when synthetic wastewater including various ions was used, whereas it improved to 2400 kg per water with the same phosphate concentration in the effluent using real wastewater.^132^ Although it was stated that the reason for this performance was the synergy between MXene and Fe_3_O_4_, Karthikeyan et al.^131^ proved that MXene alone has the same separation performance. Adsorption performance of pristine Ti_3_C_2_T_*x*_ for phosphate and nitrate ions were reported as 84 and 66 mg/g from the initial ion concentration of 100 mg/L, respectively, at pH 6. The removal mechanism of such toxic anions was attributed to the electrostatic interaction and surface complexation. Although promising studies exist, more research is required on the removal of toxic anions via MXene in the near future.

Another factor that threatens water safety is pharmaceutical compounds that have been found at trace levels in most aquatic environments due to the discharge of domestic sewage and industry. Considering the adverse effects of these compounds on human health as well as the ecosystem because of their toxicity and resistance manner, the application of efficient separation methods is the main issue for the water remediation. Adsorption has been preferred as a separation method related to its simplicity and low energy requirement even though it was restricted by the trade-off between adsorption capacity and recyclability involving as many cycles as possible. For the separation of pharmaceutical compounds, the potential of Ti_3_C_2_T_*x*_ as an adsorbent and membrane was identified by a few studies^134−136,386,387^ and further investigation is essential to completely propose its capability. Kim and colleagues^136^ investigated its removal performance and mechanism toward five pharmaceuticals with a changing molecular weight from 206 to 296 g/mol. The effect of sonication time and frequency on the removal rate of MXene was identified, where the removal rate of amitriptyline (AMT) reached to ∼66% within 120 min for the sonicated Ti_3_C_2_T_*x*_ with 28 kHz while it was only ∼47% for the pristine MXene gained within 720 min.^136^ As accepted, the more negatively charged MXene surface with the functional groups of −(OH)_2_ and −(COOH)_2_ due to the sonication yielded in the greater removal rate. Due to the positively charged AMT compared to the other investigated pharmaceuticals, MXene affinity to this cationic pharmaceutical was greater (>80 mg/g).^136^ A similar observation was proposed by the study of Ghani et al.,^134^ which defined a high adsorption capacity of Na^+^ intercalated MXene nanosheets for another positively charged pharmaceutical, ciprofloxacin (CPX). Its maximum adsorption capacity for CPX was reported as 208.2 mg/g with the removal efficiency of ∼84% at pH 5.5. To further improve CPX removal of Ti_3_C_2_T_*x*_, Ren et al.^385^ applied a rotating magnetic field for a composite adsorbent as MXene/CoFe_2_O_4_ cross-linked with SA. The created magnetic field induced the spin polarization of electrons, which facilitates the activity of functional groups of adsorbents and, hence, reduces the adsorption energy. In addition to the adsorption-based separation application of MXene, they can be used for a membrane-based separation process. Recently, Li et al.^135^ proposed a comprehensive study revealing the organic solvent nanofiltration performance of Ti_3_C_2_T_*x*_ membranes from seven different pharmaceuticals. As the molecular weight of the antibiotics increased, water or ethanol permeances enhanced with the improved rejection rates regardless of whether the antibiotic is soluble in water and ethanol, or not. The highest water permeance and rejection rate were recorded as 340.5 L/m^2^ × h × bar and 99.5% for bacitracin with a molecular weight of 1422 g/mol, whereas for penicillin with a molecular weight of 334 g/mol, the lowest permeance and rejection rate were determined as 223.1 L/m^2^ × h × bar and 89.5%.^135^ This proved that the separation mechanism of antibiotics depended on the size-selective molecular sieving with the help of the optimum interlayer distance. On the other hand, the rejection rate of tetracycline was about 97% at both pH 2 and 7, whereas it reduced to 91% at pH 4, ascribed to the change in the charges of membrane surface and antibiotic.^135^ Similarly, Gao and Chen^387^ reported a lower rejection rate of trimethoprim (80.2%) than that of sulfamethoxazole (85.5%) for the TFN membrane fabricated via interfacial polymerization of the MXene/cellulose nanocrystal nanocomposite, although the former has larger molecular weight as 290.3 g/mol than the latter (253.3 g/mol). Therefore, it was evidently revealed that electrostatic interactions dominated by the surface charge of the membrane also participates in the rejection performance of MXene. Thanks to these preliminary tests, they serve as a good starting point for this virgin field.

The separation of urine and excess water from the blood of humans is the kidney’s duty. The drop in its functioning below to 10% leads to the last phase of chronic kidney disease, end-stage renal disease (ESRD). For the treatment, renal replacement therapy with either dialysis or a kidney transplant was given to 2.6 million patients all over the world and this is estimated to double in number by 2030.^388^ In recent years, the wearable artificial kidney (WAK) emerged as a crucial alternative to transplantation and dialysis. Although there are some major challenges in the application of WAK, research on its development continues unabated. Few members of the MXene family were also examined by some groups to identify their adsorption performance of urea.^138−140^ Meng et al.^138^ tested the adsorption capacity of three different MXene nanomaterials, namely Ti_3_C_2_T_*x*_, Ti_2_CT_*x*_, and Mo_2_TiC_2_T_*x*_ for urea separation from the dialysate. The highest urea adsorption capacity was determined as 9.7 mg/g with a removal rate of 84% for Ti_3_C_2_T_*x*_ (0.155 g) at room temperature, whereas the adsorption capacity of Mo_2_TiC_2_T_*x*_ and Ti_2_CT_*x*_ were only ∼2.82 mg/g and ∼1.21 mg/g for the initial urea concentration of ∼30 mg/dL, respectively. Increasing temperature to the 37 °C, which is the body temperature, caused a further improvement in the maximum adsorption capacity of Ti_3_C_2_T_*x*_ as 10.4 mg/g.^138^ Fortunately, it was also proved that Ti_3_C_2_T_*x*_ displayed a good biocompatibility and did not have any negative effect on the blood clotting cascade, according to the pro-thrombin and partial thromboplastin coagulation analyses. Considering the actual conditions where there are some other competitive species such as creatinine and uric acid that exist in the dialysate, its removal efficiency of urea dropped to below 20%.^138^ This challenge was handled by the study of Zhao et al.^139^ via etching Ti_3_C_2_T_*x*_ using different concentrations of HF. Ti_3_C_2_T_*x*_ etched with 10 and 30 wt % HF displayed the removal efficiency of 99.7 and 55.3% for the creatinine with the initial adsorbent amount of 100 mg.^139^ This high removal rate at the low HF concentration was attributed to the presence of more hydroxyl and oxy groups but less fluoride surface terminations. To clarify the removal capacity of MXene under actual dialysis conditions, simulated dialysate including Na^+^, K^+^, Mg^2+^, Ca^2+^, chloride, acetate, bicarbonate, and dextrose were prepared. For simulated dialysate, adsorption capacities of creatinine and uric acid were 38.4 and 20.0 mg/g whereas those from aqueous solution were 45.7 and 17.0 mg/g, respectively.^139^ Therefore, it was suggested that by only adjusting the HF concentration during the etching process of MXene, the removal rate of Ti_3_C_2_T_*x*_ under real conditions could be improved. After these promising trials for the specific well-known MXene types,^138,139^ one important question has emerged that “What is the urea adsorption performance of the different MXene types?” To answer this question, Zandi et al.^140^ carried out molecular dynamics simulations to predict urea adsorption capacity of six different members of the MXene family such as Mn_2_C, Cd_2_C, Cu_2_C, Ti_2_C, W_2_C, and Ta_2_C. Considering the longer time requirement for the laboratory or clinical research, molecular simulation approaches are the best method to find a suitable nanomaterial within a large material pool in a time- and cost-effective way. Zandi et al.^140^ predicted the greatest urea adsorptions for Cd_2_C and Mn_2_C as ∼13.35 and ∼10.73 mg/g, respectively, in accordance with the order of vdW interaction energies (Cd_2_C > Mn_2_C > Cu_2_C) and total numbers of hydrogen bonding. These studies revealed that the rapidly worldwide-recognized MXene family could find a strong place in biomedical application.

The applicability of these MXene adsorbents was determined by the stability tests via continuous adsorption–desorption cycles. Removal efficiency of MXene^131^ for phosphate was around 80% with around a 17% drop after six consecutive cycles, while those for the MXene/Fe_3_O_4_ nanocomposite^132^ was ∼77% with around a 9.1% drop at the end of the 5^th^ cycle. On the other hand for the pharmaceutical separation, MXene regenerated with the electrochemical regeneration method reached the removal efficiency of 99.7% after four cycles for CPX.^134^ Moreover, adsorption performance of the sonicated MXene at 28 kHz for CPX decreased only 1% at the end of the 5^th^ cycle at pH 7.0.^136^ However, that of MXene/CoFe_2_O_4_ for CPX decreased ∼17% after five cycles.^385^ Although promising regenerability performance was suggested by these studies for MXene, unfortunately, the tested number of cycles are not enough to evaluate their applicability.

In summary, MXene membranes and adsorbents have also become rising stars for the above-mentioned distinct applications such as the separation of toxic ions, pharmaceutical, and biological compounds. Although there is not sufficient data for its practical and long-term use, its separation capacity was revealed with success. To sum up, the journey of the MXene 2D family has just started; therefore, scientists need long, detailed, and arduous studies to unveil the real performance of the MXene family.

## 10 Summary and Perspectives

The MXene family is a burgeoning class of inorganic nanomaterial families with more than 50 members, having a proven track record of intriguing characteristics. Since MXene 2D nanosheets can be stacked in parallel into ultrathin films, forming the subnanometer channels between nanosheets with a molecular size-sieving ability, they are accepted as the potential membrane nanomaterials in many separation applications. Moreover, the MXene family is also very popular for the adsorption-based separation applications due to having a high surface area, tailorable surface chemistry, and adjustable interlayer distance. As such, here in this contribution, we have reviewed the membrane and adsorbent fabrication strategies and their related properties for several separation applications in detail. In particular, we have proposed a roadmap for the development of the application of the MXene family in both membrane- and adsorption-based separation technologies. This map reveals the efficient adaptation of MXene nanomaterials into high-performance membranes and adsorbents by highlighting the relevant figures of merit. Therefore, researchers who aim to benefit from MXene nanomaterials in these two emerging separation technologies can find a foothold and determine where to begin in this booming investigation field. To unveil the potential of each MXene member for each separation application that was covered in this review, we believe future efforts should be dedicated to the following: (i) Almost thousands of distinct compositions have been accumulated, including surface terminations, multielement, multilayered MXenes, etc.^157^ However, many more layered ternary metal carbides and nitrides or more complex ones are waiting to be transformed to MXene using either MAX or non-MAX phase etching methods. Their synthesis is an important research direction to further improve the performance of the MXene family in the target areas. (ii) Additionally, it is also crucial to investigate MXene types, which have already existed in the published literature but have not been considered for the specific separation applications where they would excel. (iii) The surface engineering was used to tune the surface-related MXene properties, which further influence the separation properties of membranes and adsorbents. To determine the impact of different modification methods of MXene surfaces on separation performance, coupled with either varying T_*x*_ or covalently bonding specific molecules to the T_*x*_, should be investigated in more depth. There are some contradictory and incompatible results. (iv) New strategies to regulate the nanochannels to a stable interlayer distance are also in high demand in order to prevent swelling, not to encounter flux decline during membrane-based separation and to hinder the surface area required in adsorption-based separation. (v) Nanocomposites of MXene are proposed as another strategy to further enhance the separation performance with the mean contribution of MXene and other inorganic nanomaterials. Similarly, MXene nanomaterials have been widely envisaged as fillers in diverse polymeric matrices to fabricate MMMs. However, several hurdles still remain to be solved for MXene-based MMMs and nanocomposite membranes. These are the swelling, fouling, and instability problems, which hinder the application of MXene-based MMMs and nanocomposite membranes. (vi) In the adsorption-based separation processes, the improvement in the regeneration cycle of MXene adsorbents is a critical issue in a bid to boost their cost-effectiveness. Although promising regenerability performance compared to MOF and COF nanomaterial families was reported theoretically for the MXene family,^157^ unfortunately, the tested number of cycles are not enough to evaluate their applicability in terms of experimental studies. (vii) Similarly, in the membrane-based processes, mitigation of fouling is an important issue to retain the membrane flux and hence its applicability. Fouling appears to be a complex phenomenon that may involve multiple counteracting factors. Ti_3_C_2_T_*x*_ was suggested as the promising nanomaterial to decrease the fouling effect and the energy requirement for some of the separation processes. However, it is required to identify the fouling behavior of not only Ti_3_C_2_T_*x*_ but also other MXene types. (viii) Thick membranes endow the prolonged mass transfer path with the increased mass transfer resistance, which is against high flux. To hinder mass transfer resistance, thin membranes are required, which can be enabled by benefiting from the MXene 2D nanomaterials. However, the fabrication of thin MXene membranes in a defect free form should be investigated in depth. (ix) The most critical issue for MXene nanomaterials is the oxidation stability of the delaminated MXene dispersions. To extend the shelf life of MXene aqueous dispersions is required without the need for any additional chemicals. In addition to the promising experimental advancements in the oxidation stability of the Ti_3_C_2_T_*x*_ MXene type, the mechanism of the oxidative degradation reaction of the dispersion of other MXene types is required to be fully understood, and hence, their stabilities are also ensured. (x) Fabrication of a large amount of MXene nanomaterials^99^ as well as scalable film synthesis^389^ are required in order to use MXene nanomaterials in membrane or adsorption technologies, which bring the investigation of etching and delaminating processes for the large quantities. (xi) In addition to the lab-scale measurements, the performance of MXene membranes and adsorbents should be conducted in a real industrial process^390^ with a real feed stream composition^371^ and under practical conditions. For each separation application, that kind of investigation is very rare. (xii) For the nanomedicine and biomedical applications, mostly the cytotoxicity and biocompatibility tests of MXene to the cancer and healthy cells were performed.^391^ Unfortunately, these attempts are still in the beginning stage. Therefore, systematic investigation of toxic behavior of MXene nanomaterials and their impact on the environment and human beings should be carried out.

## Supporting Information Available

Survey of gas adsorption capacity of MXene adsorbents, solvent transport performance of MXene-based OSN and pervaporation membranes, dye removal performances of MXene-based membranes, performance of MXene-based adsorbents for heavy metal ion and radionuclides removal, and CDI desalination performance of MXene electrodes (PDF)
