# Supplementary material for: Can MXene be the Effective Nanomaterial Family for the Membrane and Adsorption Technologies to Reach a Sustainable Green World?
Source: ACS Omega. 2023 Jul 24;8(33):29859–909. doi: 10.1021/acsomega.3c01182 (PMC10448662; doi:10.1021/acsomega.3c01182)
Supplement: Supplementary file 1 — ao3c01182_si_001.pdf [file ao3c01182_si_001.pdf]

Supporting Information for

Can MXene be the effective nanomaterial family for the membrane and adsorption technologies to reach sustainable green world?

Şirin Massoumılari, Sadiye Velioğlu*

Institute of Nanotechnology, Gebze Technical University, Gebze, 41400, Kocaeli, Turkey.
Nanotechnology Research and Application Center, Gebze Technical University, 41400, Kocaeli, Turkey.

TABLE OF CONTENTS

Table S1. Survey of gas adsorption capacity of MXene adsorbents…………………………... S-2

Table S2. Survey of solvent transport performance of MXene-based OSN membranes……..S-3

Table S3. Survey of solvent transport performance of MXene-based pervaporation membranes…… S-7

Table S4. Survey of dye removal performances of MXene-based membranes from aqueous solutions at 1 bar and 25°C. S-8

Table S5. Survey of performance of MXene-based adsorbents for heavy metal ion removal ……………………………………………………………………………………………S-12

Table S6. Survey of performance of MXene-based adsorbents for the removal of radionuclides………………………………………………………………………………..S-15

Table S7. Survey of CDI desalination performance of MXene electrodes ……………………………………………………………………………….………………...S-17

References……………………………………………………………………………….S-18

Table S1. Survey of gas adsorption capacity of MXene adsorbents.

Adsorbents	Operating Conditions	CO_2	CH_4	H_2	Unit	Ref.
Ti_2C [computed]	NR	8.25			mol/kg	[1]
V_2C [computed]	NR	7.80			mol/kg	[1]
Zr_2C [computed]	NR	4.57			mol/kg	[1]
Nb_2C [computed]	NR	4.49			mol/kg	[1]
Mo_2C [computed]	NR	4.36			mol/kg	[1]
Hf_2C [computed]	NR	2.41			mol/kg	[1]
Ta_2C [computed]	NR	2.37			mol/kg	[1]
W_2C [computed]	NR	2.34			mol/kg	[1]
$Ti_3C_2T_x$	40 bar, 25°C	1.33			mol/kg	[2]
$Ti_3C_2T_x$ intercalated with DMSO	40 bar, 25°C	5.79			mol/kg	[2]
V_2CT_x	40 bar, 25°C	0.52			mol/kg	[2]
V_2CT_x intercalated with DMSO	40 bar, 25°C	0.77			mol/kg	[2]
$Ti_3C_2T_x$ etched with LiF*	60 bar, 25°C		8.5		cm^3 (STP)/g	[3]
$Ti_3C_2T_x$ etched with NaF*	60 bar, 25°C		3.80		cm^3 (STP)/g	[3]
$Ti_3C_2T_x$ etched with KF*	60 bar, 25°C		4.87		cm^3 (STP)/g	[3]
$Ti_3C_2T_x$ etched with NH_4F*	60 bar, 25°C		6.70		cm^3 (STP)/g	[3]
Ti_2CT_x etched with LiF*	60 bar, 25°C		8.28		cm^3 (STP)/g	[3]
Ti_2CT_x etched with NaF*	60 bar, 25°C		10.04		cm^3 (STP)/g	[3]
Ti_2CT_x etched with KF*	60 bar, 25°C		11.6		cm^3 (STP)/g	[3]
Ti_2CT_x etched with NH_4F*	60 bar, 25°C		7.60		cm^3 (STP)/g	[3]
Ti_2CT_x	50 bar, 25°C		11.58		cm^3 (STP)/g	[4]
Ti_2CT_x	50 bar, 40°C		18.18		cm^3 (STP)/g	[4]
Ti_2CT_x	50 bar, 50°C		52.76		cm^3 (STP)/g	[4]
Ti_2CT_x intercalated with $NH_3.H_2O$	50 bar, 25°C		16.81		cm^3 (STP)/g	[4]
Ti_2CT_x incompletely etched with HF	60 bar, 25°C			8.8	wt.%	[5]
$Ti_3C_2T_x$-NIM	10 bar, 25°C	1.80			mol/kg	[6]
$Ti_3C_2T_x$	0.01 bar, 125°C	~12			mol/kg	[7]

DMSO: dimethyl sulfoxide, NIM: nanoscale ionic materials, NR: not reported. *Intercalated with DMSO, $NH_3.H_2O$, and urea.

Table S2. Survey of solvent transport performance of MXene-based ***OSN*** membranes. Membrane thickness is given in parenthesis.

**Membranes**	**Operating Conditions**	**Solvent**	**Solvent flux ($L/m^2 \times h \times bar$) (*$L/m^2 \times h$)**	**Ref.**
$Ti_3C_2T_x$ on PDA (0.23 µm)	1 bar, 25°C	2-propanol	982.7	8
$Ti_3C_2T_x$ on PDA (0.23 µm)	1 bar, 25°C	Acetone	5022.4	8
$Ti_3C_2T_x$ on PDA (0.23 µm)	1 bar, 25°C	Acetonitrile	5346.49	8
$Ti_3C_2T_x$ on PDA (0.23 µm)	1 bar, 25°C	Methanol	3559.74	8
$Ti_3C_2T_x$ on PDA (0.23 µm)	1 bar, 25°C	Ethanol	1908.72	8
$Ti_3C_2T_x$ on PDA (0.23 µm)	1 bar, 25°C	DMF	1595.82	8
$Ti_3C_2T_x$ on PC (0.09 µm)	5 bar, 25°C	IPA	0.79	9
$Ti_3C_2T_x$ on PC (0.09 µm)	5 bar, 25°C	Hexane	6.62	9
$Ti_3C_2T_x$ on PC (0.09 µm)	5 bar, 25°C	Toluene	3.17	9
$Ti_3C_2T_x$ on PC (0.09 µm)	5 bar, 25°C	c-hexane	2.14	9
$Ti_3C_2T_x$ on nylon (0.14 µm)	0.5 bar, 25°C	Acetone	4451*	10
$Ti_3C_2T_x$ on nylon (0.14 µm)	0.5 bar, 25°C	IPA	530*	10
$Ti_3C_2T_x$ on nylon (0.14 µm)	0.5 bar, 25°C	Ethanol	1088*	10
$Ti_3C_2T_x$ on nylon (0.14 µm)	0.5 bar, 25°C	Methanol	1890*	10
$Ti_3C_2T_x$ (70wt.%)/GO on nylon (0.14 µm)	0.5 bar, 25°C	Acetone	48.32*	10
$Ti_3C_2T_x$ (70wt.%)/GO on nylon (0.14 µm)	0.5 bar, 25°C	IPA	6.18*	10
$Ti_3C_2T_x$ (70wt.%)/GO on nylon (0.14 µm)	0.5 bar, 25°C	Ethanol	10.76*	10
$Ti_3C_2T_x$ (1 wt.%)/PEI on PAN (0.545 µm)	10 bar, 25°	isopropanol	28.72*	11
$Ti_3C_2T_x$ (2 wt.%)/PEI on PAN (0.545 µm)	10 bar, 25°	isopropanol	33.54*	11
$Ti_3C_2T_x$ (3 wt.%)/PEI on PAN (0.545 µm)	10 bar, 25°	isopropanol	26.74*	11
$Ti_3C_2T_x$ (4 wt.%)/PEI on PAN (0.545 µm)	10 bar, 25°	isopropanol	23.35*	11
$Ti_3C_2T_x$ (1 wt.%)/PEI on PAN (0.545 µm)	10 bar, 25°	n-heptane	4.072*	11
$Ti_3C_2T_x$ (2 wt.%)/PEI on PAN (0.545 µm)	10 bar, 25°	n-heptane	4.594*	11
$Ti_3C_2T_x$ (3 wt.%)/PEI on PAN (0.545 µm)	10 bar, 25°	n-heptane	2.363*	11
$Ti_3C_2T_x$ (4 wt.%)/PEI on PAN (0.545 µm)	10 bar, 25°	n-heptane	2.069*	11
$Ti_3C_2T_x$ (1 wt.%)/PEI on PAN (0.545 µm)	10 bar, 25°	Ethyl Acetate	15*	11
$Ti_3C_2T_x$ (2 wt.%)/PEI on PAN (0.545 µm)	10 bar, 25°	Ethyl Acetate	12.3*3	11
$Ti_3C_2T_x$ (3 wt.%)/PEI on PAN (0.545 µm)	10 bar, 25°	Ethyl Acetate	11.26*	11
$Ti_3C_2T_x$ (4 wt.%)/PEI on PAN (0.545 µm)	10 bar, 25°	Ethyl Acetate	9.43*	11
$Ti_3C_2T_x$ (1 wt.%)/PDMS on PAN (0.545 µm)	10 bar, 25°	isopropanol	6.01*	11
$Ti_3C_2T_x$ (3 wt.%)/PDMS on PAN (0.545 µm)	10 bar, 25°	isopropanol	6.92*	11
$Ti_3C_2T_x$ (5 wt.%)/PDMS on PAN (0.545 µm)	10 bar, 25°	isopropanol	7.06*	11
$Ti_3C_2T_x$ (10 wt.%)/PDMS on PAN (0.545 µm)	10 bar, 25°	isopropanol	8.41*	11

$Ti_3C_2T_x$ (1 wt.%)/PDMS on PAN (0.545 µm)	10 bar, 25°	toluene	17.52*	[11]
$Ti_3C_2T_x$ (3 wt.%)/PDMS on PAN (0.545 µm)	10 bar, 25°	toluene	11.97*	[11]
$Ti_3C_2T_x$ (5 wt.%)/PDMS on PAN (0.545 µm)	10 bar, 25°	toluene	9.785*	[11]
$Ti_3C_2T_x$ (10 wt.%)/PDMS on PAN (0.545 µm)	10 bar, 25°	toluene	13.84*	[11]
$Ti_3C_2T_x$ (1 wt.%)/PDMS on PAN (0.545 µm)	10 bar, 25°	Ethyl Acetate	11.98*	[11]
$Ti_3C_2T_x$ (3 wt.%)/PDMS on PAN (0.545 µm)	10 bar, 25°	Ethyl Acetate	8.79*	[11]
$Ti_3C_2T_x$ (5 wt.%)/PDMS on PAN (0.545 µm)	10 bar, 25°	Ethyl Acetate	6.42*	[11]
$Ti_3C_2T_x$ (10 wt.%)/PDMS on PAN (0.545 µm)	10 bar, 25°	Ethyl Acetate	11.16*	[11]
$Ti_3C_2T_x$-NH_2 (3wt.%)/PEI on PAN (0.2 µm)	10 bar, 25°C	IPA	31.08*	[12]
$Ti_3C_2T_x$-NH_2 (3wt.%)/PEI on PAN (0.2 µm)	10 bar, 25°C	Ethyl acetate	15.07*	[12]
$Ti_3C_2T_x$-NH_2 (3wt.%)/PEI on PAN (0.2 µm)	10 bar, 25°C	Toluene	3.08*	[12]
$Ti_3C_2T_x$-NH_2 (3wt.%)/PEI on PAN (0.2 µm)	10 bar, 25°C	n-Heptane	2.43*	[12]
$Ti_3C_2T_x$-COOR (3wt.%)/PEI on PAN (0.2 µm)	10 bar, 25°C	IPA	29.39*	[12]
$Ti_3C_2T_x$-COOR (3wt.%)/PEI on PAN (0.2 µm)	10 bar, 25°C	Ethyl acetate	17.75*	[12]
$Ti_3C_2T_x$-COOR (3wt.%)/PEI on PAN (0.2 µm)	10 bar, 25°C	Toluene	3.26*	[12]
$Ti_3C_2T_x$-COOR (3wt.%)/PEI on PAN (0.2 µm)	10 bar, 25°C	n-Heptane	2.79*	[12]
$Ti_3C_2T_x$-C_6H_6 (3wt.%)/PDMS on PAN (0.2 µm)	10 bar, 25°C	IPA	2.71*	[12]
$Ti_3C_2T_x$-C_6H_6 (3wt.%)/PDMS on PAN (0.2 µm)	10 bar, 25°C	Ethyl acetate	4.55*	[12]
$Ti_3C_2T_x$-C_6H_6 (3wt.%)/PDMS on PAN (0.2 µm)	10 bar, 25°C	Toluene	11.96*	[12]
$Ti_3C_2T_x$-C_6H_6 (3wt.%)/PDMS on PAN (0.2 µm)	10 bar, 25°C	n-Heptane	16.37*	[12]
$Ti_3C_2T_x$-$C_{12}H_{26}$ (3wt.%)/PDMS on PAN (0.2 µm)	10 bar, 25°C	IPA	2.66*	[12]
$Ti_3C_2T_x$-$C_{12}H_{26}$ (3wt.%)/PDMS on PAN (0.2 µm)	10 bar, 25°C	Ethyl acetate	4.45*	[12]
$Ti_3C_2T_x$-$C_{12}H_{26}$ (3wt.%)/PDMS on PAN (0.2 µm)	10 bar, 25°C	Toluene	11.43*	[12]
$Ti_3C_2T_x$-$C_{12}H_{26}$ (3wt.%)/PDMS on PAN (0.2 µm)	10 bar, 25°C	n-Heptane	17.8*	[12]
$Ti_3C_2T_x$ on nylon (0.3-0.5 µm)	2.7 bar, 25°C	Toluene	752	[13]
$Ti_3C_2T_x$ on nylon (0.3-0.5 µm)	2.7 bar, 25°C	Acetonitrile	3050	[13]
$Ti_3C_2T_x$ on nylon (0.3-0.5 µm)	2.7 bar, 25°C	Acetone	2455.17	[13]
$Ti_3C_2T_x$ on nylon (0.3-0.5 µm)	2.7 bar, 25°C	Methanol	2561	[13]
$Ti_3C_2T_x$ on nylon (0.3-0.5 µm)	2.7 bar, 25°C	Ethyl acetate	1877	[13]
$Ti_3C_2T_x$/PEI on nylon (0.34 µm)	2.7 bar, 25°C	Toluene	219.48	[13]
$Ti_3C_2T_x$/PEI on nylon (0.34 µm)	2.7 bar, 25°C	Acetonitrile	1654.53	[13]
$Ti_3C_2T_x$/PEI on nylon (0.34 µm)	2.7 bar, 25°C	Acetone	1275.37	[13]
$Ti_3C_2T_x$/PEI on nylon (0.34 µm)	2.7 bar, 25°C	Methanol	1669	[13]
$Ti_3C_2T_x$/PEI on nylon (0.34 µm)	2.7 bar, 25°C	Ethyl acetate	966.31	[13]
$Ti_3C_2T_x$-C_6H_5 on nylon (0.3-0.5 µm)	2.7 bar, 25°C	Toluene	908.26	[13]
$Ti_3C_2T_x$-C_6H_5 on nylon (0.3-0.5 µm)	2.7 bar, 25°C	Acetonitrile	1409.34	[13]

$Ti_3C_2T_x$-C_6H_5 on nylon (0.3-0.5 µm)	2.7 bar, 25°C	Acetone	1515.21	13
$Ti_3C_2T_x$-C_6H_5 on nylon (0.3-0.5 µm)	2.7 bar, 25°C	Methanol	902.20	13
$Ti_3C_2T_x$-C_6H_5 on nylon (0.3-0.5 µm)	2.7 bar, 25°C	Ethyl acetate	1116	13
$Ti_3C_2T_x$/PDMS on nylon (0.32 µm)	2.7 bar, 25°C	Toluene	860.20	13
$Ti_3C_2T_x$/PDMS on nylon (0.32 µm)	2.7 bar, 25°C	Acetonitrile	661	13
$Ti_3C_2T_x$/PDMS on nylon (0.32 µm)	2.7 bar, 25°C	Acetone	587.06	13
$Ti_3C_2T_x$/PDMS on nylon (0.32 µm)	2.7 bar, 25°C	Methanol	675	13
$Ti_3C_2T_x$/PDMS on nylon (0.32 µm)	2.7 bar, 25°C	Ethyl acetate	781	13
$Ti_3C_2T_x$ on nylon (0.32 µm)	1-5 bar, 25°C	1-butanol	968	14
$Ti_3C_2T_x$ on nylon (0.32 µm)	1-5 bar, 25°C	isopropanol	1415	14
$Ti_3C_2T_x$ on nylon (0.32 µm)	1-5 bar, 25°C	Ethanol	2073	14
$Ti_3C_2T_x$ on nylon (0.32 µm)	1-5 bar, 25°C	Methanol	3393	14
$Ti_3C_2T_x$ on nylon (0.32 µm)	1-5 bar, 25°C	n-hexane	1500	14
$Ti_3C_2T_x$ on nylon (0.32 µm)	1-5 bar, 25°C	Toluene	900	14
$Ti_3C_2T_x$-$C_{12}H_{25}$ on nylon (0.318 µm)	1-5 bar, 25°C	1-butanol	103	14
$Ti_3C_2T_x$-$C_{12}H_{25}$ on nylon (0.318 µm)	1-5 bar, 25°C	isopropanol	205	14
$Ti_3C_2T_x$-$C_{12}H_{25}$ on nylon (0.318 µm)	1-5 bar, 25°C	Ethanol	400	14
$Ti_3C_2T_x$-$C_{12}H_{25}$ on nylon (0.318 µm)	1-5 bar, 25°C	Methanol	893	14
$Ti_3C_2T_x$-$C_{12}H_{25}$ on nylon (0.318 µm)	1-5 bar, 25°C	n-hexane	1513	14
$Ti_3C_2T_x$-$C_{12}H_{25}$ on nylon (0.318 µm)	1-5 bar, 25°C	Toluene	927	14
$Ti_3C_2T_x$-NH_2 on nylon (0.337 µm)	1-5 bar, 25°C	Acetonitrile	2780	14
$Ti_3C_2T_x$-NH_2 on nylon (0.337 µm)	1-5 bar, 25°C	Acetone	2200	14
$Ti_3C_2T_x$-NH_2 on nylon (0.337 µm)	1-5 bar, 25°C	Ethyl Acetate	1650	14
$Ti_3C_2T_x$-NH_2 on nylon (0.337 µm)	1-5 bar, 25°C	THF	1550	14
$Ti_3C_2T_x$-NH_2 on nylon (0.337 µm)	1-5 bar, 25°C	Methanol	2500	14
$Ti_3C_2T_x$-NH_2 on nylon (0.337 µm)	1-5 bar, 25°C	DMF	1420	14
$Ti_3C_2T_x$-NH_2 on nylon (0.337 µm)	1-5 bar, 25°C	Ethanol	1280	14
$Ti_3C_2T_x$-NH_2 on nylon (0.337 µm)	1-5 bar, 25°C	IPA	1050	14
$Ti_3C_2T_x$-NH_2 on nylon (0.337 µm)	1-5 bar, 25°C	Butanol	980	14
$Ti_3C_2T_x$-NH_2 on nylon (0.337 µm)	1-5 bar, 25°C	n-hexane	1380	14
$Ti_3C_2T_x$-NH_2 on nylon (0.337 µm)	1-5 bar, 25°C	Toluene	760	14
$Ti_3C_2T_x$ (0.8 g/l)/PA on nylon (0.0012-0.0014 µm) (AL: feed solution)	1 bar, 25°C	Ethanol	8.1	15
$Ti_3C_2T_x$ (0.8 g/l)/PA on nylon (0.0012-0.0014 µm) (AL: draw solution)	1 bar, 25°C	Ethanol	9.5	15
$Ti_3C_2T_x$ (0.1mg/cm^2) on nylon (0.36 µm)	0.5 bar, 25°C	Acetone	140	16
$Ti_3C_2T_x$ (0.1mg/cm^2) on nylon (0.36 µm)	0.5 bar, 25°C	Methanol	121	16
$Ti_3C_2T_x$ (0.1mg/cm^2) on nylon (0.36 µm)	0.5 bar, 25°C	Ethanol	67	16

$Ti_3C_2T_x$ (0.1mg/cm^2) on nylon (0.36 µm)	0.5 bar, 25°C	Isopropanol	48	16
$Ti_3C_2T_x$ (0.1mg/cm^2) on nylon (0.36 µm)	0.5 bar, 25°C	N-butanol	29	16
$Ti_3C_2T_x$ (0.1mg/cm^2)-crumbled on nylon (0.58 µm)	0.5 bar, 25°C	Acetone	3745	16
$Ti_3C_2T_x$ (0.1mg/cm^2)-crumbled on nylon (0.58 µm)	0.5 bar, 25°C	Methanol	2484	16
$Ti_3C_2T_x$ (0.1mg/cm^2)-crumbled on nylon (0.58 µm)	0.5 bar, 25°C	Ethanol	1363	16
$Ti_3C_2T_x$ (0.1mg/cm^2)-crumbled on nylon (0.58 µm)	0.5 bar, 25°C	Isopropanol	276	16
$Ti_3C_2T_x$ (0.1mg/cm^2)-crumbled on nylon (0.58 µm)	0.5 bar, 25°C	N-butanol	599	16
$Ti_3C_2T_x$ (0.03mg/cm^2)-crumbled on nylon (0.32 µm)	0.5 bar, 25°C	Acetone	3886	16
$Ti_3C_2T_x$ (0.03mg/cm^2)-crumbled on nylon (0.32 µm)	0.5 bar, 25°C	Methanol	3459	16
$Ti_3C_2T_x$ (0.03mg/cm^2)-crumbled on nylon (0.32 µm)	0.5 bar, 25°C	Ethanol	2051	16
$Ti_3C_2T_x$ (0.03mg/cm^2)-crumbled on nylon (0.32 µm)	0.5 bar, 25°C	Isopropanol	1440	16
$Ti_3C_2T_x$ (0.03mg/cm^2)-crumbled on nylon (0.32 µm)	0.5 bar, 25°C	N-butanol	1274	16
$Ti_3C_2T_x$ on PEI (0.496 µm)	3 bar, 25°C	Methanol	29	17
$Ti_3C_2T_x$ on PEI (0.496 µm)	3 bar, 25°C	Ethanol	14	17
$Ti_3C_2T_x$ on PEI (0.496 µm)	3 bar, 25°C	Isopropanol	7.5	17
$Ti_3C_2T_x$ on PEI (0.496 µm)	3 bar, 25°C	DMF	8	17
$Ti_3C_2T_x$/CNT-CTAB (2/2) on PEI (1.1 µm)	3 bar, 25°C	Methanol	115	17
$Ti_3C_2T_x$/CNT-CTAB (2/2) on PEI (1.1 µm)	3 bar, 25°C	Ethanol	52	17
$Ti_3C_2T_x$/CNT-CTAB (2/2) on PEI (1.1 µm)	3 bar, 25°C	Isopropanol	30	17
$Ti_3C_2T_x$/CNT-CTAB (2/2) on PEI (1.1 µm)	3 bar, 25°C	DMF	35	17
$Ti_3C_2T_x$/PFA/P84 (0.077 µm)	7 bar, 25°C	Methanol	6.7	18
$Ti_3C_2T_x$/PFA/P84 (0.077 µm)	7 bar, 25°C	Ethanol	3.1	18
$Ti_3C_2T_x$/PFA/P84 (0.077 µm)	7 bar, 25°C	Isopropanol	0.4	18
$Ti_3C_2T_x$//PFA/P84 (0.077 µm)	7 bar, 25°C	Acetone	7.3	18
$Ti_3C_2T_x$/PFA/P84 (0.077 µm)	7 bar, 25°C	Acetonitrile	5.8	18
$Ti_3C_2T_x$/PFA/P84 (0.077 µm)	7 bar, 25°C	Hexane	0.1	18

AL: Active Layer, CNT: carbon nanotube, CTAB: hexadecyl trimethyl ammonium bromide, DMF: dimethylformamide, GO: graphene oxide, PA: polyamide, PAN: polyacrylonitrile, PC: polycarbonate, PDMS: polydimethylsiloxane, PEI: polyethyleneimine, PFA: potassium fulvic acid, P84: commercial copolyimide.

Table S3. Survey of solvent transport performance of MXene-based ***pervaporation*** membranes. Membrane thickness is given in parenthesis.

Membranes	Operating Conditions	Solvent	Total flux of mixture (g/m^2×h)	Separation factor	Ref.
Ti_2CT_x-HPEI/TMC on PAN (0.01 µm)	1 bar, 50°C	Methanol (90 wt.%)	~2237		19
Ti_2CT_x-HPEI/TMC on PAN (0.01 µm)	1 bar, 50°C	Ethanol (90 wt.%)	~1434		19
Ti_2CT_x-HPEI /TMC on PAN (0.01 µm)	1 bar, 50°C	IPA (90 wt.%)	~1020.1	~1260	19
Ti_2CT_x on PAN (0.25 µm)	1 bar, 50°C	IPA (90 wt.%)	~5120		20
Ti_2CT_x/PEI on PAN (0.25 µm)	1 bar, 50°C	IPA (90 wt.%)	~1875		20
Ti_2CT_x/PAH on PAN (0.25 µm)	1 bar, 50°C	IPA (90 wt.%)	~1672		20
Ti_2CT_x/PDDA on PAN (0.25 µm)	1 bar, 50°C	IPA (90 wt.%)	1237	1931	20
$Ti_3C_2T_x$ on PAN (0.5 µm)	0.35 bar, 50°C	Ethanol (10 wt.%)	~1150	407	21
$Ti_3C_2T_x$ (3wt.%)/CS on PAN (0.5 µm)	0.35 bar, 50°C	Ethanol (10 wt.%)	1424	1421	21
$Ti_3C_2T_x$ on PAN (0.5 µm)	0.35 bar, 50°C	Ethyl acetate (2 wt.%)	~1000	~750	21
$Ti_3C_2T_x$ (3wt.%)/CS on PAN (0.5 µm)	0.35 bar, 50°C	Ethyl acetate (2 wt.%)	1471	4898	21
$Ti_3C_2T_x$ on PAN (0.5 µm)	0.35 bar, 50°C	Dimethyl carbonate (2 wt.%)	~1080	~390	21
$Ti_3C_2T_x$ (3wt.%)/CS on PAN (0.5 µm)	0.35 bar, 50°C	Dimethyl carbonate (2 wt.%)	1428	906	21
$Ti_3C_2T_x$ (12wt.%)/SA on PAN (7.8 µm)	1 bar, 70°C	Ethanol (90 wt.%)	505	9946	22
$Ti_3C_2T_x$ (24wt.%)/SA on PAN (7.8 µm)	1 bar, 70°C	Ethanol (90 wt.%)	704	~1000	22
$Ti_3C_2T_x$ (0.5wt.%)/PVA on PAN (6 µm)	0.2 bar, 37°C	Ethanol (93 wt.%)	95.44	722.78	23
$Ti_3C_2T_x$ (1wt.%)/PVA on PAN (6 µm)	0.2 bar, 37°C	Ethanol (93 wt.%)	89.65	2017.75	23
$Ti_3C_2T_x$ (2wt.%)/PVA on PAN (6 µm)	0.2 bar, 37°C	Ethanol (93 wt.%)	79.78	2169.56	23
$Ti_3C_2T_x$ (3wt.%)/PVA on PAN (6 µm)	0.2 bar, 37°C	Ethanol (93 wt.%)	73.39	2556.98	23
$Ti_3C_2T_x$ on PAN (6 µm)	0.2 bar, 37°C	Ethanol (93 wt.%)	95.82	134.76	23
$Ti_3C_2T_x$ (4wt.%)/PVA on PAN (6 µm)	0.2 bar, 37°C	Ethanol (93 wt.%)	60.48	1838.67	23
$Ti_3C_2T_x$ (2 wt.%)/PVA-SSA on PTFE (0.23 µm)	1 bar, 30°C	Methanol (96 wt.%)	~1500	968	24
$Ti_3C_2T_x$ (2 wt.%)/PVA-SSA on PTFE (0.23 µm)	1 bar, 30°C	Ethanol (96 wt.%)	1489	4738	24
$Ti_3C_2T_x$ (2 wt.%)/PVA-SSA on PTFE (0.23 µm)	1 bar, 30°C	IPA (96 wt.%)	1120	7913	24
$Ti_3C_2T_x$ (2 wt.%)/PVA-SSA on PTFE (0.23 µm)	1 bar, 30°C	Tert-butanol (96 wt.%)	1030	23786	24
$Ti_3C_2T_x$ on AAO (2 µm)	1 bar, 25°C	Ethanol (75 wt.%)	234.3	14.1	25
$Ti_3C_2T_x$ on AAO (2 µm)	1 bar, 25°C	Ethanol (90 wt.%)	258.8	92.8	25
$Ti_3C_2T_x$ on AAO (2 µm)	1 bar, 25°C	Ethanol (95 wt.%)	263.4	135.2	25
$Ti_3C_2T_x$ on AAO (1 µm)	1 bar, 25°C	Ethanol (90 wt.%)	~370	~24	25
$Ti_3C_2T_x$ on AAO (3 µm)	1 bar, 25°C	Ethanol (90 wt.%)	~50	~100	25

AAO: anodic aluminum oxide, CS: chitosan, HPEI: hyperbranched polyethyleneimine, PAH: polyallylamine hydrochloride, PAN: polyacrylonitrile, PDDA: poly (diallyl dimethylammonium chloride), PEI: polyethyleneimine, PTFE: polytetrafluoroethylene, PVA: poly (vinyl alcohol), SA: sodium alginate, SSA: sulfosuccinic acid, TMC: trimesoyl chloride.

Table S4. Survey of dye removal performances of MXene-based membranes from aqueous solutions at 1 bar and 25°C. Membrane thickness is given in parenthesis. *Measurements were performed at 5 bar.

Membrane	Dye	Total water permeance (L/m^2×h×bar)	Rejection Rate (%)	Ref.
$Ti_3C_2T_x$ on AAO (0.4 µm)	EB	254	97.7	26
$Ti_3C_2T_x$ (embedded with $Fe(OH)_3$) on AAO (0.4 µm)	EB	103	99.6	26
$Ti_3C_2T_x$ (embedded, removed $Fe(OH)_3$) on AAO (0.4 µm)	EB	1084	90	26
$Ti_3C_2T_x$ (embedded, removed $Fe(OH)_3$) on AAO (0.4 µm)	RhB	805	85	26
$Ti_3C_2T_x$ (embedded, removed $Fe(OH)_3$) on AAO (0.4 µm)	TMPyP	921	93	26
$Ti_3C_2T_x$ (0.1 gr) on PES	CR	210	50	27
$Ti_3C_2T_x$ (0.2 gr) on PES	CR	115	92.3	27
$Ti_3C_2T_x$ (0.2 gr) on PES	GV	117.6	80.3	27
$Ti_3C_2T_x$ (0.4 g/cm^2) on MCE (0.25-0.4 µm)	MB	64.4	~98	28
$Ti_3C_2T_x$ (0.6 g/cm^2) on MCE (0.25-0.4 µm)	MB	44.97	100	28
$Ti_3C_2T_x$ (0.23 µm) on PDA modified Nylon	AY14	~910	57	8
$Ti_3C_2T_x$ (0.23 µm) on PDA modified Nylon	CD	~970	91	8
$Ti_3C_2T_x$ (0.23 µm) on PDA modified Nylon	RB	~935	96	8
$Ti_3C_2T_x$ (0.23 µm) on PDA modified Nylon	AY79	~985	100	8
$Ti_3C_2T_x$ (produced slot-die coating) on PES	MR	200	93.2	29
$Ti_3C_2T_x$ (produced slot-die coating) on PES	MB	200	99.9	29
$Ti_3C_2T_x$ (produced slot-die coating) on PES	BBG	200	98.2	29
$Ti_3C_2T_x$ (produced slot-die coating) on PES	EB	200	87.8	29
$Ti_3C_2T_x$ (produced slot-die coating) on PES	RB	200	96.9	29
$Ti_3C_2T_x$ (1.5 µm) (0 voltage) on PVDF	MB	10.6	98	30
$Ti_3C_2T_x$ (1.5 µm) (0 voltage) on PVDF	MB	10498	97.9	30
$Ti_3C_2T_x$ (10 wt.%)/PVA (1.5 µm) (0 voltage)	MB	7.4	97.9	30
$Ti_3C_2T_x$ (10 wt.%)/PVA (1.5 µm) (-0.5 voltage)	MB	5.6	99.6	30
$Ti_3C_2T_x$ (10 wt.%)/PVA (1.5 µm) (0.1 voltage)	MB	60.1	92.5	30
$Ti_3C_2T_x$ (0.32 µm)	MO		33	14
$Ti_3C_2T_x$ (0.32 µm)	MB		81.85	14
$Ti_3C_2T_x$ (0.32 µm)	RR24		95.29	14
$Ti_3C_2T_x$ (0.32 µm)	AY14		99.5	14
$Ti_3C_2T_x$ (0.32 µm)	RBl		99.5	14
$Ti_3C_2T_x$ (0.32 µm)	AY79		100	14
$Ti_3C_2T_x$ (functionalized with $–NH_2$) (0.337 µm)	MO		74	14
$Ti_3C_2T_x$ (functionalized with $–NH_2$) (0.337 µm)	MB		92.49	14
$Ti_3C_2T_x$ (functionalized with $–NH_2$) (0.337 µm)	RR24		96.28	14
$Ti_3C_2T_x$ (functionalized with $–NH_2$) (0.337 µm)	AY14		99.02	14
$Ti_3C_2T_x$ (functionalized with $–NH_2$) (0.337 µm)	RBl		99.8	14
$Ti_3C_2T_x$ (functionalized with $–NH_2$) (0.337 µm)	AY79		99.9	14
$Ti_3C_2T_x$ (functionalized with $–C_{12}H_{25}$) (0.318 µm)	MO		30	14
$Ti_3C_2T_x$ (functionalized with $–C_{12}H_{25}$) (0.318 µm)	MB		42	14
$Ti_3C_2T_x$ (functionalized with $–C_{12}H_{25}$) (0.318 µm)	RR24		91	14
$Ti_3C_2T_x$ (functionalized with $–C_{12}H_{25}$) (0.318 µm)	AY14		97	14
$Ti_3C_2T_x$ (functionalized with $–C_{12}H_{25}$) (0.318 µm)	RBl		99	14
$Ti_3C_2T_x$ (functionalized with $–C_{12}H_{25}$) (0.318 µm)	AY79		100	14
$Ti_3C_2T_x$ (functionalized with $–C_6H_5$) (0.332 µm)	MO		24	14
$Ti_3C_2T_x$ (functionalized with $–C_6H_5$) (0.332 µm)	MB		34	14
$Ti_3C_2T_x$ (functionalized with $–C_6H_5$) (0.332 µm)	RR24		51	14

$Ti_3C_2T_x$ (functionalized with $-C_6H_5$) (0.332 µm)	AY14		96	14
$Ti_3C_2T_x$ (functionalized with $-C_6H_5$) (0.332 µm)	RBl		97	14
$Ti_3C_2T_x$ (functionalized with $-C_6H_5$) (0.332 µm)	AY79		100	14
$Ti_3C_2T_x$ (0.25 mg/cm^2)	AY14	135	80.8	16
$Ti_3C_2T_x$ (0.25 mg/cm^2)	EB	135	95.7	16
$Ti_3C_2T_x$ (0.25 mg/cm^2)	EY	135	95.4	16
$Ti_3C_2T_x$ (0.01 mg/cm^2)	AY14	2694	55.7	16
$Ti_3C_2T_x$ (0.01 mg/cm^2)	EB	2694	53.5	16
$Ti_3C_2T_x$ (0.01 mg/cm^2)	EY	2694	88.1	16
$Ti_3C_2T_x$ (crumpled) (0.25 mg/cm^2)	AY14	1247	76.4	16
$Ti_3C_2T_x$ (crumpled) (0.25 mg/cm^2)	EB	1247	67.7	16
$Ti_3C_2T_x$ (crumpled) (0.25 mg/cm^2)	EY	1247	84.3	16
$Ti_3C_2T_x$ (crumpled) (0.01 mg/cm^2)	AY14	5460	17.9	16
$Ti_3C_2T_x$ (crumpled) (0.01 mg/cm^2)	EB	5460	16.2	16
$Ti_3C_2T_x$ (crumpled) (0.01 mg/cm^2)	EY	5460	72.2	16
$Ti_3C_2T_x$ (etched over 60 min. for porosity)	MO	42.48	61.9	31
$Ti_3C_2T_x$ (etched over 60 min. for porosity)	RhB	42.48	87.9	31
$Ti_3C_2T_x$ (etched over 60 min. for porosity)	CR	42.48	100	31
$Ti_3C_2T_x$ on Nylon (0.35 µm)	MV	47	90.56	32
$Ti_3C_2T_x$ on Nylon (0.35 µm)	CR	66	95.77	32
$Ti_3C_2T_x$ (small lateral size) on nylon (0.1 µm)	MB	6.2	88.3	33
$Ti_3C_2T_x$ (medium lateral size) on nylon (0.1 µm)	MB	5.1	94.7	33
$Ti_3C_2T_x$ (large lateral size) on nylon (0.1 µm)	MB	4.2	95.6	33
$Ti_3C_2T_x$ on MCE	MB		50	34
$Ti_3C_2T_x$ (grafted with MNOCS) on MCE	MB		52	34
$Ti_3C_2T_x$ (grafted with MODCS) on MCE	MB		55	34
$Ti_3C_2T_x$ (grafted with MTPCS) on MCE	MB		70	34
$Ti_3C_2T_x$ (1 wt.%)/P84 MMM	GV	268	100	35
$Ti_3C_2T_x$ (1 wt.%)/P84 MMM	CR	380	78.5	35
$Ti_3C_2T_x$ (1 wt.%)/P84 MMM	MO	408	4	35
$Ti_3C_2T_x$ (10 wt.%)/CA (crosslinked) (123 µm)	RhB	256	92.3	36
$Ti_3C_2T_x$ (10 wt.%)/CA (crosslinked) (123 µm)	MG	256	98.3	36
$Ti_3C_2T_x$ (Silk encapsulated)	Coomassie BB	85.6	100	37
$Ti_3C_2T_x$ (Silk encapsulated)	Pyranine	125.9	60.3	37
$Ti_3C_2T_x$ (Silk encapsulated)	PMR	73.7	94.6	37
$Ti_3C_2T_x$ (Silk encapsulated)	R6G	94.7	98.8	37
$Ti_3C_2T_x$ (Silk encapsulated)	MB	66.2	98.9	37
$Ti_3C_2T_x$ (20 wt.%) (intercalated into the ANF) (10 µm)	AB	195.3	98.9	38
$Ti_3C_2T_x$ (20 wt.%) (intercalated into the ANF) (10 µm)	RB	195.3	97.8	38
$Ti_3C_2T_x$ (20 wt.%) (intercalated into the ANF) (10 µm)	CR	195.3	94.2	38
$Ti_3C_2T_x$/PA on PDA coated nylon-66	CR	514.5	99.6	39
$Ti_3C_2T_x$/PA on PDA coated nylon-66	EBT	508.6	98.3	39
$Ti_3C_2T_x$/PA on PDA coated nylon-66	MB	520	83	39
$Ti_3C_2T_x$/PA on PDA coated nylon-66	RB	485	92	39
$Ti_3C_2T_x$ (intercalated with Fe ion)	CR	261.6	93.36	40
$Ti_3C_2T_x$ (intercalated with Fe ion)	RhB	261.6	88.56	40
$Ti_3C_2T_x$ (intercalated with Cu ion)	CR	259.7	92.41	40
$Ti_3C_2T_x$ (intercalated with Cu ion)	RhB	259.7	87.40	40
$Ti_3C_2T_x$ (intercalated with Zn ion)	CR	259.7	96	40
$Ti_3C_2T_x$ (intercalated with Zn ion)	RhB	259.7	95	40

$Ti_3C_2T_x$ on CA (10 µm)	Direct Red 28	61.9	84.2	41
$Ti_3C_2T_x$ on CA (10 µm)	Direct black 38	57.5	84.3	41
$Ti_3C_2T_x$ (cross-linked with PDA) on CA (20 µm)	Direct Red 28	236	88.9	41
$Ti_3C_2T_x$ (cross-linked with PDA) on CA (20 µm)	Direct black 38	248	88.6	41
$Ti_3C_2T_x$/PIL_{CN} TFSI on Nylon-66 (0.225 µm)	CR	420	96.1	42
$Ti_3C_2T_x$/PIL_{CN} TFSI on Nylon-66 (0.225 µm)	BT	350	99.5	42
$Ti_3C_2T_x$/PIL_{CN} TPB on Nylon-66 (0.225 µm)	CR	479	92.7	42
$Ti_3C_2T_x$/ZIF-8/PES (1% microemulsion content)	RhB	140	98	43
$Ti_3C_2T_x$/ZIF-8/PES (3% microemulsion content)	RhB	280	96	43
Nb_2CT_x/SA on PVDF (5 µm)	Rh6G	~2164	100	44
Nb_2CT_x/SA on PVDF (5 µm)	BB	~2002	100	44
Nb_2CT_x/SA on PVDF (5 µm)	Toluidine	~2209	100	44
$Ti_3C_2T_x$/GO (30 wt.%) on AAO (0.09 µm)*	MR	2.1	68	9
$Ti_3C_2T_x$/GO (30 wt.%) on AAO (0.09 µm)*	MB	0.3	99.5	9
$Ti_3C_2T_x$/GO (30 wt.%) on AAO (0.09 µm)*	RB	0.67	93.5	9
$Ti_3C_2T_x$/GO (30 wt.%) on AAO (0.09 µm)*	BB	0.23	100	9
$Ti_3C_2T_x$ (70 wt.%)/GO on nylon (0.14 µm)	MO	~18.2	~97.6	10
$Ti_3C_2T_x$ (70 wt.%)/GO on nylon (0.14 µm)	MB	~17.6	~99.6	10
$Ti_3C_2T_x$ (70 wt.%)/GO on nylon (0.14 µm)	AY14	~21.4	~97.6	10
$Ti_3C_2T_x$ (70 wt.%)/GO on nylon (0.14 µm)	IC	~23.2	~99.8	10
$Ti_3C_2T_x$ (70 wt.%)/GO on nylon (0.14 µm)	Eosin	~24.0	~99.3	10
$Ti_3C_2T_x$/GO (4/1) on MCE (0.55 µm)	CG	71.9	~97.0	45
$Ti_3C_2T_x$/GO (4/1) on MCE (0.55 µm)	NR	71.9	~99.5	45
$Ti_3C_2T_x$/GO (4/1) on MCE (0.55 µm)	MB	71.9	~99.5	45
$Ti_3C_2T_x$/GO (4/1) on MCE (0.55 µm)	CV	71.9	~99.5	45
$Ti_3C_2T_x$/GO (4/1) on MCE (0.55 µm)	BB	71.9	~99.5	45
$Ti_3C_2T_x$ on PVDF (0.47 µm)	RhB	90.3	81.0	46
$Ti_3C_2T_x$ on PVDF (0.47 µm)	MG	84.5	94.1	46
$Ti_3C_2T_x$/Ag (21 wt.%) on PVDF (0.47 µm)	RhB	387.1	79.9	46
$Ti_3C_2T_x$/Ag (21 wt.%) on PVDF (0.47 µm)	MG	354.3	92.3	46
$Ti_3C_2T_x$/UiO-66-$(COOH)_2$ (1/1.5 mg) on nylon 66	MB	743	99.84	47
$Ti_3C_2T_x$/UiO-66-$(COOH)_2$ (1/1.5 mg) on nylon 66	n-hexane-in-MB	443	>99	47
$Ti_3C_2T_x$/UiO-66-$(COOH)_2$ (1/1.5 mg) on nylon 66	isooctane-in-MB	488	>99	47
$Ti_3C_2T_x$/UiO-66-$(COOH)_2$ (1/1.5 mg) on nylon 66	TMT-in-MB	328	>99	47
$Ti_3C_2T_x$/UiO-66-$(COOH)_2$ (1/1.5 mg) on nylon 66	Toluene-in-MB	446	>99	47
$Ti_3C_2T_x$/rGO (160/40 mg) on nylon	MB	174	95	48
$Ti_3C_2T_x$/rGO (160/40 mg) on nylon	MO	~90	~99.5	48
$Ti_3C_2T_x$/rGO (160/40 mg) on nylon	MR	~89	~98.4	48
$Ti_3C_2T_x$/rGO (160/40 mg) on nylon	CR	~116	~97.6	48
$Ti_3C_2T_x$/rGO (160/40 mg) on nylon	EB	~109	~95.8	48
$Ti_3C_2T_x$/Al_2O_3 (1/1) (1.75µm)	MB	88.8	99.5	49
$Ti_3C_2T_x$/Al_2O_3 (1/1) (1.75µm)	OG	84.0	97.2	49
$Ti_3C_2T_x$/Al_2O_3 (1/1) (1.75µm)	RhB	86.5	99.8	49
$Ti_3C_2T_x$/Al_2O_3 (1/1) (1.75µm)	OF	75.0	87.2	49
$Ti_3C_2T_x$/CNTs (treated at 80°C) on PDA (0.135 µm)	CR	10.8	>99	50
$Ti_3C_2T_x$/CNTs (treated at 80°C) on PDA (0.135 µm)	RhB	11.1	94.9	50
$Ti_3C_2T_x$/CNTs (treated at 80°C) on PDA (0.135 µm)	MO	13.2	92.4	50
$Ti_3C_2T_x$/CNTs (treated at 120°C) on PDA (0.104 µm)	CR	7.6	>99	50
$Ti_3C_2T_x$/CNTs (treated at 120°C) on PDA (0.104 µm)	RhB	8.5	96.4	50
$Ti_3C_2T_x$/CNTs (treated at 120°C) on PDA (0.104 µm)	MO	9.4	93.9	50

$Ti_3C_2T_x$/CNTs (treated at 180°C) on PDA (0.078 µm)	CR	4.2	>99	50
$Ti_3C_2T_x$/CNTs (treated at 180°C) on PDA (0.078 µm)	RhB	5.3	97.8	50
$Ti_3C_2T_x$/CNTs (treated at 180°C) on PDA (0.078 µm)	MO	6.2	95.4	50
$Ti_3C_2T_x$/CNTs-CTAB (2/2) on PEI (0.98µm)	MB	99.5	~0	17
$Ti_3C_2T_x$/CNTs-CTAB (2/2) on PEI (0.98µm)	OII	99.5	~13.7	17
$Ti_3C_2T_x$/CNTs-CTAB (2/2) on PEI (0.98µm)	AF	99.5	~76.97	17
$Ti_3C_2T_x$/CNTs-CTAB (2/2) on PEI (0.98µm)	CR	99.5	~95.8	17
$Ti_3C_2T_x$/CNTs-CTAB (2/2) on PEI (0.98µm)	FG	99.5	~99.7	17
$Ti_3C_2T_x$/GO (1/1) (dopamine functionalized) on PVDF (2µm)	CR	75	74.0	51
$Ti_3C_2T_x$/GO (1/2) (dopamine functionalized) on PVDF (2µm)	CR	61.6	98.1	51
$Ti_3C_2T_x$/CNT (45%) on Nylon (0.82µm)	CV	1215	99.8	52
$Ti_3C_2T_x$/CNT (45%) on Nylon (0.82µm)	MO	1290	95.3	52
$Ti_3C_2T_x$/COF (2/1) on MCE	MB	800	65.0	53
$Ti_3C_2T_x$/COF (4/1) on MCE	MB	218	99.7	53
$Ti_3C_2T_x$/COF (4/1) on MCE	FB	185	100	53
$Ti_3C_2T_x$/COF (4/1) on MCE	CV	204	100	53
$Ti_3C_2T_x$/COF (4/1) on MCE	EV	170	99.3	53
$Ti_3C_2T_x$/COF (4/1) on MCE	MG	190	99.8	53
$Ti_3C_2T_x/TiO_2/Bi_2S_3$ (30%) on CA (5.38µm)	RhB	220	100	54
$Ti_3C_2T_x/TiO_2/Bi_2S_3$ (30%) on CA (5.38µm)	MO	245	75.0	54
$Ti_3C_2T_x/TiO_2/Bi_2S_3$ (30%) on CA (5.38µm)	GR	215	100	54
$Ti_3C_2T_x/TiO_2/Bi_2S_3$ (30%) on CA (5.38µm)	MB	230	100	54
$Ti_3C_2T_x$ on nylon	MB	18.5	100	55
$Ti_3C_2T_x$/CM-β-CD (30 mg) on nylon (30.3µm)	MB	431	99.7	55
$Ti_3C_2T_x$/CM-β-CD (30 mg) on nylon (30.3µm)	BR	~467	99.2	55
$Ti_3C_2T_x$/CM-β-CD (30 mg) on nylon (30.3µm)	CV	~455	99.7	55
$Ti_3C_2T_x$/CM-β-CD (30 mg) on nylon (30.3µm)	RhB	~438	97.0	55
$Ti_3C_2T_x$/CM-β-CD (30 mg) on nylon (30.3µm)	Methyl B	366	98.8	55
$Ti_3C_2T_x$/CM-β-CD (30 mg) on nylon (30.3µm)	MO	~470	86.1	55
$Ti_3C_2T_x$/CB5 on PES (0.50 µm)	MB	69	93.6	56
$Ti_3C_2T_x$/CB5 on PES (0.60 µm)	MB	53.5	97.3	56
$Ti_3C_2T_x$/CB5 on PES (0.70 µm)	MB	42.3	99.1	56

AAO: anodic aluminum oxide, ANF: aramid nanofiber, Bi_2S_3: bismuth sulphide, CA: cellulose acetate, CB5: Cucurbit[5]uril, CM-β-CD: carboxymethyl-β-cyclodextrin, CNTs: carbon nanotubes, COF: covalent organic frameworks, CTAB: hexadecyl trimethyl ammonium bromide, D: dopamine, GO: graphene oxide, MCE: mixed cellulose ester, MMM: mixed matrix membranes, MNOCS: n-octyltrichlorosilane, MODCS: n-octadecyltrichlorosilane, MTPCS: triphenylchlorosilane, PDA: polydopamine, PEI: polyetherimide, PES: polyethersulfone , PILCN: Poly(1-vinyl-3-cyanomethylimidazoliumbromide) (CN refers to the distal group in the substituent of the polyimidazolium backbone), PVA: poly(vinyl alcohol), PVDF: polyvinylidene fluoride, P84: commercial copolyimide, rGO: reduced graphene oxide, SA: sodium alginate, TFSI: bis(trifluoromethyl sulfonyl)imide, TiO_2: titanium dioxide, TPB: tetraphenylborate.

AB: Alcian Blue, AF: Acid Fuchsin, AY14: Acid Yellow 14, AY79: Acid Yellow 79, BB: Brilliant Blue, BBG: Brilliant Blue G, BR: Basic Red, BT: Eriochrome Black T, CD: Carbon Dot, CG: Chrysoidine G, CR: Congo Red, CV: Crystal Violet, EB: Evans Blue, EBT: Eriochrome Black T, EV: Ethyl Violet, EY: Eosin Y, FB: Fuchsin Basic FG: Fast Green, GR: Gram Stain, GV: Gentian Violet, IC: Indigo Carmine, MB: Methylene Blue, MG: Methyl Green, MO: Methyl Orange, MR: Methyl Red, NR: Neutral Red, OF: Neutral Ofloxacin, OG: Orange G, OII: Orange II, PMR: Paramethyl Red, RB: Rose Bengal, RBl: Reactive Black, RhB: Rhodamine B, Rh6G: Rhodamine 6G, RR 24: Reactive Red 24, R6G: Rhodamine 6G, TMPyP: 5,10,15,20-tetrakis-(N-methyl-4-pyridyl)-21,23-H-porphyrin tetratosylate, TMT: 1,3,5-trimethyltoluene.

Table S5. Survey of performance of MXene-based adsorbents for heavy metal ion removal. Please note that some adsorption values are read from the relevant figures where they are represented. Table is organized based on the type of heavy metal ions.

Adsorbent	d-spacing*/ interlayer spacing (Å)	Surface area (m^2/g)	Test conditions (T: °C, pH)	Adsorbent dosage (mg/L)	Heavy Metal	Heavy Metal Concentration (mg/L)	Adsorption Capacity (Q_{max}*/Q_e) (mg/g)	Ref.
$Ti_3C_2T_x$	4.35*	15	25, 7	15	BrO_3^-	4.83	322	57
Ti_2CT_x (Alkaline treated nanosheet)	—	66.7	25, 6	330	Cd(II)	561.35	294	58
Ti_2CT_x (Alkaline treated nanofiber)	—	50.1	25, 6	330	Cd(II)	561.35	125	58
Ti_3AlC_2-derived (Cellulose nanofiber)	—	38.9	25, 6	1000	Cd(II)	100	63.2	59
Ti_3AlC_2-derived (Glucose nanofiber)	—	37.0	25, 6	1000	Cd(II)	100	58.5	59
Ti_3AlC_2-derived (Sawdust nanofiber)	—	16.2	25, 6	1000	Cd(II)	100	39.9	59
$Ti_3C_2T_x$ (DMSO intercalated dry state)	6.28	—	25, 5	12	Cd(II)	100	20.5	60
$Ti_3C_2T_x$ (DMSO intercalated hydrated phase)	20.18	—	25, 5	12	Cd(II)	100	34.1	60
$Ti_3C_2T_x$	—	10	20, 6	50	Cd(II)	2	31.7	61
$Ti_3C_2T_x$/PEI/SA (Amino-functionalized)	—	16.31	25, 3	200	Cr(VI)	50	131	62
$Ti_3C_2T_x$	14.6	10.4	30, 2	100	Cr(VI)	100	137	63
$Ti_3C_2T_x$/PmPD	17.6	55.9	30, 2	100	Cr(VI)	100	540	63
$Ti_3C_2T_x$ (10 wt.% HF treated)	15.1	57	25, 5	200	Cr(VI)	207.76	250	64
$Ti_3C_2T_x$ (25 wt.% HF treated)	—	20	25, 5	200	Cr(VI)	207.76	170	64
$Ti_3C_2T_x$ (50 wt.% HF treated)	—	9	25, 5	200	Cr(VI)	207.76	120	64
$Ti_3C_2T_x$	—	17.7	25, 2.5	667	Cr(VI)	100	56.2	65
$Ti_3C_2T_x$ (Amino-functionalized)	—	13	25, 2.5	667	Cr(VI)	100	93.5	65
$Ti_3C_2(OH)_{0.8}F_{1.2}$	4.92*	—	25, 5.8-6.4	500	Cr(VI)	30-60	62	66
Urchin-like TiO_2-C/TiC (Derived from MXene)	4.92*	—	25, 5.8-6.4	500	Cr(VI)	30-60	225	66
$Ti_3C_2T_x$	—	7	25, 2	100	Cr(VI)	100	30.6	67
$Ti_3C_2T_x$/nZVI	—	31.2	25, 2	100	Cr(VI)	100	195	67
$Ti_3C_2T_x$	—	11.2	30, 3	100	Cr(VI)	100	81	68
$Ti_3C_2T_x$	13.8	19.6	25, 5.14	500	Cr(VI)	20	68.2	69
$Ti_3C_2T_x$/rGO film	13.9	125.5	25, 5.14	500	Cr(VI)	20	69.6	69
$Ti_3C_2T_x$/rGO film (HCl treated)	13.6	—	25, 5.14	500	Cr(VI)	20	84	69
$Ti_3C_2T_x$	—	6.24	25, —	1000	Cr(VI)	100	80	70
$Ti_3C_2T_x$	—	42.9	25, 3	1000	Cr(VI)	5	1.5	71
$Ti_3C_2T_x$/δ-MnO_2	—	129.3	25, 3	1000	Cr(VI)	5	2.8	71
$Ti_3C_2T_x$/PEI/SA (SA: 5 g)	—	16.31	25, 2	200	Cr(VI)	100	311.86	62
$Ti_3C_2T_x$	—	—	25, 2	200	Cr(VI)	30	31.4	72
$Ti_3C_2T_x$ (Imidizolated)	—	9.769	25, 2	200	Cr(VI)	30	119.5	72

$Ti_3C_2T_x$ (Chitosan functionalized)	—	—	25, 4.3	600	Cr(VI)	100	50.6	73
$Ti_3C_2T_x$	—	—	30, 2	200	Cr(VI)	30	125.32	74
$Ti_3C_2T_x$ (Amino-functionalized, alkaline treated)	13.6	129.2	25, 6.3	100	Cu(II)	25	118	75
$Ti_3C_2T_x/TiO_2$ (Amino acid intercalated (His))	13.99	—	25, —	50	Cu(II)	500	94.6	76
$Ti_3C_2T_x$/Alginate	—	—	25, 5	50	Cu(II)	96	87.6	77
$Ti_3C_2T_x$ (Delaminated)	25	67.7	25, 5	500	Cu(II)	25	78.5	78
$Ti_3C_2T_x$ (DMSO intercalated dry state)	6.28	—	25, 5	12	Cu(II)	100	23.0	60
$Ti_3C_2T_x$ (DMSO intercalated hydrated phase)	20.18	—	25, 5	12	Cu(II)	100	43.7	60
Ti_3AlC_2-derived (Cellulose nanofiber)	—	38.9	25, 5	1000	Cu(II)	100	41.6	59
Ti_3AlC_2-derived (Sawdust nanofiber)	—	16.2	25, 5	1000	Cu(II)	100	40.0	59
Ti_3AlC_2-derived (Glucose nanofiber)	—	37.0	25, 5	1000	Cu(II)	100	38.3	59
$Ti_3C_2T_x$	—	—	25, 7	200	Cu(II)	10	10	79
$Ti_3C_2T_x$/PDOPA	—	—	25, 7	200	Cu(II)	10	18.36	79
$Ti_3C_2T_x$	—	10	20, 6	50	Cu(II)	2	35.4	61
$Ti_3C_2T_x/CoFe_2O_4$/SA	—	10.51	25, 5.5	225	Cu(II)	100	96.85	80
$Ti_3C_2T_x$	—	—	30, 5	1000	Cu(II)	50	21.88	81
$Ti_3C_2T_x$ (Functionalized with EHL (50 wt.%))	—	—	30, 5	1000	Cu(II)	50	44.35	81
$Ti_3C_2T_x$ (Amino-functionalized, alkaline treated)	13.6	129.2	25, 6.3	100	Ni(II)	25	18.43	75
$Ti_3C_2T_x$	—	—	25, 7	500	Ni(II)	100	52.9*	82
$Ti_3C_2T_x$/LDH (Alkaline treated)	—	—	25, 7	500	Ni(II)	100	223*	82
$Ti_3C_2T_x$ (Delaminated)	8.8	6.37	25, 6.3	100	Pb(II)	25	130	75
$Ti_3C_2T_x$ (Alkaline treated)	11.4	72.04	25, 6.3	100	Pb(II)	25	187	75
$Ti_3C_2T_x$ (Amino-functionalized, alkaline treated)	13.6	129.2	25, 6.3	100	Pb(II)	25	221.5	75
$Ti_3C_2T_x$/Alginate	—	—	25, 5	50	Pb(II)	96	383	77
$Ti_3C_2T_x$ (Nanofiber)	—	16.4	25, 5	100	Pb(II)	20	286	83
$Ti_3C_2T_x$ (Nanosheet)	—	1.6	25, 5	100	Pb(II)	20	218	83
$Ti_3C_2T_x$ (Powder)	9.83	8.53	30, 5	500	Pb(II)	500	48.28	84
$Ti_3C_2T_x$ (Modified with 10 wt.% KH570)	12.6	75.4	30, 5	3200	Pb(II)	500	147	84
$Ti_3C_2(OH/ONa)_xF_{2-x}$ (Alkaline treated)	15.1	—	25, 5.8−6.2	500	Pb(II)	50	140.1	85
Ti_2CT_x	11.8	13.3	30, 5	1600	Pb(II)	200	81.5	86
Ti_2CT_x (Functionalized with enzymatic hydrolysis lignin)	14.7	22.5	30, 5	1600	Pb(II)	200	122.4	86
Ti_2CT_x (Functionalized with lignosulfonate)	15.5	—	30, 5	1600	Pb(II)	200	104.4	86
Ti_2CT_x (Functionalized with chitosan)	14.3	—	30, 5	1600	Pb(II)	200	93.5	86
$Ti_3C_2T_x$	—	10	20, 6	50	Pb(II)	2	36.6	61
$Ti_3C_2T_x$	—	19.6	25, 5.14	500	Ag(I)	20	691.4	69

$Ti_3C_2T_x$/rGO film	—	125.5	25, 5.14	500	Ag(I)	20	1137	[69]
$Ti_3C_2T_x$/rGO film (HCl treated)	—	—	25, 5.14	500	Ag(I)	20	1172	[69]
$Ti_3C_2T_x$ aerogel	—	—	25, 0.7-4.15	250	Ag(I)	99.885	1736.1*	[87]
$Ti_3C_2T_x$/rGO hybrid aerogel	—	9.0753	25, 0.7-4.15	250	Ag(I)	99.885	1183.4*	[87]
$Ti_3C_2T_x$	—	16.0	30, —	—	Au(III)	19.97	3200	[88]
$Ti_3C_2T_x$/CNT	—	181.6	30, —	—	Au(III)	19.97	2093	[88]
$Ti_3C_2T_x$	—	19.6	25, 5.14	500	Au(III)	20	158	[69]
$Ti_3C_2T_x$/rGO film	—	125.5	25, 5.14	500	Au(III)	20	883	[69]
$Ti_3C_2T_x$/rGO film (HCl treated)	—	—	25, 5.14	500	Au(III)	20	1241	[69]
$Ti_3C_2T_x$ aerogel	—	—	25, 0.7-4.15	250	Au(III)	100	1851.8*	[87]
$Ti_3C_2T_x$/rGO hybrid aerogel	—	9.0753	25, 0.7-4.15	250	Au(III)	100	1063.8*	[87]
Ti_3C_2 (Multi-layer oxygen functionalized)	10.6	3.48	25, 5	100	Hg(II)	10.4	4806*	[89]
$Ti_3C_2T_x$/MoS_2 (Delaminated)	—	9.6	25, 6.5	10	Hg(II)	2000	1435	[90]
$Ti_3C_2T_x$/Fe_2O_3	—	56.5	25, 6	25	Hg(II)	1000	1128	[91]
$Ti_3C_2T_x$/$SA_{4:20}$	—	9.66	25, 6	50	Hg(II)	1000	10.53	[58]
$Ti_3C_2T_x$/$SA_{2:20}$	—	—	25, 6	50	Hg(II)	1000	7.69	[58]
$Ti_3C_2T_x$	—	—	30, 4	100	Hg(II) [from $Hg(NO_3)_2$]	100	221	[92]
$Ti_3C_2T_x$	—	—	30, 4	100	Hg(II) [from $HgCl_2$]	100	187	[92]
$Ti_3C_2T_x$	—	—	25, 3.9	333	Hg(II)	25	5070*	[93]
Ti_3CNT_x	—	—	25, 3.9	333	Hg(II)	25	4263*	[93]
$Ti_3C_2T_x$	—	19.6	25, 5.14	500	Pd(II)	20	359	[69]
$Ti_3C_2T_x$/rGO film	—	125.5	25, 5.14	500	Pd(II)	20	793	[69]
$Ti_3C_2T_x$/rGO film (HCl treated)	—	—	25, 5.14	500	Pd(II)	20	890	[69]
$Ti_3C_2T_x$ (HF treated at 45°C)	3.1*	76.4	20, —	500	Pd(II)	100	185	[94]
$Ti_3C_2T_x$ (HF treated at 35°C)	2.8*	65.4	20, —	500	Pd(II)	100	164	[94]
$Ti_3C_2T_x$ (HF treated at 25°C)	2.2*	19.8	20, —	500	Pd(II)	100	119	[94]
$Ti_3C_2T_x$ (Amino-functionalized, alkaline)	13.6	129.2	25, 6.3	100	Zn(II)	25	22.2	[75]
$Ti_3C_2T_x$	—	10	20, 6	50	Zn(II)	2	32	[61]

CFO: $CoFe_2O_4$, CNT: carbon nanotube, DMSO: dimethyl sulfoxide, EHL: enzymatic hydrolysis lignin, KH570: glycidoxypropyltrimethoxysilane, LDH: layered double hydroxide, nZVI: nanoscale zero-valent iron, PDOPA: polymerized levodopa, PEI: polyethyleneimine, PmPD: poly(m-phenylenediamine), rGO: reduced graphene oxide, SA: sodium alginate.

Table S6. Survey of performance of MXene-based adsorbents for the removal of radionuclides. Please note that some adsorption values are read from the relevant figures where they are represented. Table is organized based on the type of radionuclides.

Adsorbent	d-spacing*/ interlayer spacing (Å)	Surface area (m^2/g)	Test conditions (T:°C, pH)	Adsorbent dosage (mg/L)	Radionuclides	Radionuclides Concentration (mg/L)	Adsorption Capacity (Q_{max}*/Q_e) (mg/g)	Ref.
$Ti_3C_2T_x$	—	13	25, 7	100	Ba(II)	55	9.3*	95
$Ti_3C_2T_x$	—	10	20, 7	1000	Ba(II)	2000	180	96
$Ti_3C_2T_x$	—	9.78	20, 7	500	Ba(II)	100	12.0	97
$Ti_3C_2T_x$ (Alkaline treated)	20.9	76.4	20, 7	500	Ba(II)	100	46.5	97
$Ti_3C_2T_x$ (Prussian blue-incorporated)	—	—	25, 5.6	2000	Cs(I)	10	23.3	98
$Ti_3C_2T_x$	—	10	20, 7	5	Cs(I)	2	148	99
$Ti_3C_2T_x$	7.4	42.9	25, 6	10	Cs(I)	5	25.4	100
$Ti_3C_2T_x$	9.5	—	25, 7	—	Cs(I)	100	110*	101
$Ti_3C_2T_x$ (POSS–NH_2 intercalated)	11.8	—	25, 7	—	Cs(I)	100	148*	101
Ti_2CT_x (Na-intercalated)	10.5	184	25, 4	200	Eu(III)	50	158	102
Ti_2CT_x (K-intercalated)	9.6	347	25, 4	200	Eu(III)	50	127	102
$Ti_3C_2T_x$ (Carboxyl functionalized)	—	—	25, 5	200	Eu(III)	200	97.1*	103
$Ti_3C_2T_x$ (Alkaline treated)	—	—	25, 6	300	Eu(III)	500	82.74	104
$Ti_3C_2T_x$	—	8.9	25, 5	200	Eu(III)	10	32.9*	105
$Ti_3C_2T_x$/LDH	—	49.2	25, 5	200	Eu(III)	10	97.1*	105
$Ti_3C_2T_x$ (Amidoxime functionalized)	—	—	25, 5	100	Eu(III)	20	74*	106
$Ti_3C_2T_x$/PDA/Ag_2O_x	—	54.8	25, 5.6	200	I(I)	20	80	107
$Ti_3C_2T_x$	—	—	25, —	100	I(I)	254	27	108
$Ti_3C_2T_x$ (–NH_2 functionalized)	—	—	25, —	100	I(I)	254	40	108
$Ti_3C_2T_x$ (Poly ionic liquid functionalized)	—	—	25, —	100	I(I)	254	170	108
Ti_2CT_x	10.8	31	30, 4	400	Re(VII)	100	10.5	109
Ti_2CT_x/PDDA	14.5	18	30, 4	400	Re(VII)	100	121	109
Ti_2CT_x (Multi layered)	—	—	30, 4	400	Re(VII)	100	9.0	109
Ti_2CT_x/PDDA (Multi layered)	—	5	30, 4	400	Re(VII)	100	33.2	109
$Ti_3C_2T_x$	—	10	20, 7	1000	Sr(II)	2000	225	96
$Ti_3C_2T_x$	9.5	—	25, 7	—	Sr(II)	100	138*	101
$Ti_3C_2T_x$ (POSS–NH_2 intercalated)	11.8	—	25, 7	—	Sr(II)	100	172*	101
Ti_2CT_x (Hydrated phase)	11.4	—	25, 3	400	Th(IV)	100	159	110
Ti_2CT_x (Dry phase)	9.28	—	25, 3	400	Th(IV)	100	27	110
$Ti_3C_2T_x$	—	—	25, 3.4	400	Th(IV)	100	103	111
$Ti_3C_2T_x$ (Dry phase)	—	—	25, 3.4	400	Th(IV)	100	37	111

$Ti_3C_2T_x$ (DMSO intercalated)	—	—	25, 3.4	400	Th(IV)	100	138	[111]
$Ti_3C_2T_x$	—	—	25, 3.5	80	U(VI)	100	70	[112]
$Ti_3C_2T_x$ (Alkaline treated)	—	—	25, 3.5	80	U(VI)	100	155	[112]
$Ti_3C_2T_x$/nZVI (Alkaline treated)	—	—	25, 3.5	80	U(VI)	100	1246	[112]
Ti_2CT_x (Multi layered)	11.3	—	25, 3	400	U(VI)	200	470	[113]
$Ti_3C_2T_x$ (Carboxyl functionalized)	—	—	25, 5	200	U(VI)	200	345*	[103]
$Ti_3C_2T_x$ (Amidoxime functionalized)	—	—	25, 5	200	U(VI)	200	97	[114]
V_2CT_x (Multi layered)	23.7	—	25, 4.5	400	U(VI)	100	174	[115]
$Ti_3C_2T_x$ (Dry phase)	1.52	—	25, 5	400	U(VI)	100	25	[60]
$Ti_3C_2T_x$ (Na-intercalated dry phase)	6.38	—	25, 5	400	U(VI)	100	105	[60]
$Ti_3C_2T_x$ (DMSO-intercalated dry phase)	6.16	—	25, 5	400	U(VI)	100	63	[60]
$Ti_3C_2T_x$ (Hydrated phase)	7.52	—	25, 5	400	U(VI)	100	96	[60]
$Ti_3C_2T_x$ (Na-intercalated hydrated)	12.3	—	25, 5	400	U(VI)	100	116	[60]
$Ti_3C_2T_x$ (DMSO-intercalated hydrated)	20.2	—	25, 5	400	U(VI)	100	158	[60]
$Ti_3C_2T_x$	—	8.9	25, 5	200	U(VI)	10	56.9*	[105]
$Ti_3C_2T_x$/LDH	—	49.2	25, 5	200	U(VI)	10	241*	[105]
$Ti_3C_2T_x$	—	—	25, 5	100	U(VI)	20	111*	[106]
$Ti_3C_2T_x$ (Amidoxime-functionalized)	—	—	25, 5	100	U(VI)	20	280*	[106]
$Ti_3C_2T_x$/POSS–OH (Cross-linked with HPC)	9.9	—	25, 5	—	U(VI)	300	308	[116]

DMSO: dimethyl sulfoxide, HPC: hydroxypropyl cellulose, LDH: layered double hydroxide, nZVI: zero valent iron nanoparticle, PDA: polydopamine, PDDA: poly(diallyldimethylammonium chloride), POSS: polyhedral oligomeric silsesquioxane.

Table S7. Survey of CDI desalination performance of MXene electrodes.

Electrode Materials (Cathode)	Specific Surface Area (m^2/g)	Initial Salt Concentration (mg/L)	Applied Voltage (V)	Desalination Capacity (mg/g)	Tested Cycle Number	Ref.
$Ti_3C_2T_x$/NTP nanohybrid	24.3	1,000	1.8	128.6	20	[117]
$Ti_3C_2T_x$ (large-small flakes mixture)	–	292	1.6	72	50	[118]
$Ti_3C_2T_x$ (less $-F_2$ terminal groups)	2.1	585	1.2	67.7	50	[119]
$Ti_3C_2T_x$ aerogel (porous architecture)	293	10,000	1.2	45	60	[120]
$Ti_3C_2T_x$ sheet (nitrogen-doped)	369	5,000	1.2	43.5	24	[121]
$Ti_3C_2T_x$	–	600	1.2	26.5	10	[122]
$Ti_3C_2T_x$/CLF composite	−	600	1.2	35	10	[122]
$Ti_3C_2T_x$ (Ar plasma modified)	−	500	1.4	26.8	–	[123]
$Ti_3C_2T_x$	−	500	1.2	12.2	20	[124]
$Ti_3C_2T_x$ (Na^+-intercalated)	−	500	1.2	16	20	[124]
$Mo_{1.33}CT_x$	1	293	0.8	5	40	[125]
$Mo_{1.33}CT_x$/CNT nanocomposite	30	35,100	0.8	15	40	[125]
$Ti_3C_2T_x$	–	293	1.2	13	30	[126]
$Ti_3C_2T_x$ (HF-etched)	–	95.5	1.6	2	−	[127]
$Ti_3C_2T_x$ (NH_4HF_2-etched)	17	498	1.6	12.1	−	[127]
$Ti_3C_2T_x$/CNT nanocomposite	12	35,100	1.2	8	100	[128]
$Ti_3C_2T_x$ (preconditioned)	–	585	1.2	9.2	100	[129]
$Ti_3C_2T_x$	–	500	1.2	460	10	[130]

CLF: cellulose fiber, CNT: carbon nanotube, NH_4HF_2: ammonium bifluoride, NTP: $NaTi_2(PO_4)_3$, sodium titanium phosphate.

References

1. Morales-García, Á.; Fernández-Fernández, A.; Viñes, F.; Illas, F., CO_2 abatement using two-dimensional MXene carbides. *Journal of Materials Chemistry A* **2018,** *6* (8), 3381-3385.
2. Wang, B.; Zhou, A.; Liu, F.; Cao, J.; Wang, L.; Hu, Q., Carbon dioxide adsorption of two-dimensional carbide MXenes. *Journal of Advanced Ceramics* **2018,** *7*, 237-245.
3. Liu, F.; Zhou, A.; Chen, J.; Jia, J.; Zhou, W.; Wang, L.; Hu, Q., Preparation of Ti_3C_2 and Ti_2C MXenes by fluoride salts etching and methane adsorptive properties. *Applied Surface Science* **2017,** *416*, 781-789.
4. Liu, F.; Zhou, A.; Chen, J.; Zhang, H.; Cao, J.; Wang, L.; Hu, Q., Preparation and methane adsorption of two-dimensional carbide Ti_2C. *Adsorption* **2016,** *22*, 915-922.
5. Liu, S.; Liu, J.; Liu, X.; Shang, J.; Xu, L.; Yu, R.; Shui, J., Hydrogen storage in incompletely etched multilayer Ti_2CT_x at room temperature. *Nature Nanotechnology* **2021,** *16* (3), 331-336.
6. Wang, D.; Ning, H.; Xin, Y.; Wang, Y.; Li, X.; Yao, D.; Zheng, Y.; Pan, Y.; Zhang, H.; He, Z., Transforming $Ti_3C_2T_x$ MXenes into nanoscale ionic materials via an electronic interaction strategy. **2021**.
7. Persson, I.; Halim, J.; Lind, H.; Hansen, T. W.; Wagner, J. B.; Näslund, L. Å.; Darakchieva, V.; Palisaitis, J.; Rosen, J.; Persson, P. O., 2D transition metal carbides (MXenes) for carbon capture. *Advanced Materials* **2019,** *31* (2), 1805472.
8. Wang, J.; Chen, P.; Shi, B.; Guo, W.; Jaroniec, M.; Qiao, S. Z., A regularly channeled lamellar membrane for unparalleled water and organics permeation. *Angewandte Chemie International Edition* **2018,** *57* (23), 6814-6818.
9. Kang, K. M.; Kim, D. W.; Ren, C. E.; Cho, K. M.; Kim, S. J.; Choi, J. H.; Nam, Y. T.; Gogotsi, Y.; Jung, H.-T., Selective Molecular Separation on $Ti_3C_2T_x$–Graphene Oxide Membranes during Pressure-Driven Filtration: Comparison with Graphene Oxide and MXenes. *ACS applied materials & interfaces* **2017,** *9* (51), 44687-44694.
10. Wei, S.; Xie, Y.; Xing, Y.; Wang, L.; Ye, H.; Xiong, X.; Wang, S.; Han, K., Two-dimensional graphene Oxide/MXene composite lamellar membranes for efficient solvent permeation and molecular separation. *Journal of Membrane Science* **2019,** *582*, 414-422.
11. Wu, X.; Hao, L.; Zhang, J.; Zhang, X.; Wang, J.; Liu, J., Polymer-$Ti_3C_2T_x$ composite membranes to overcome the trade-off in solvent resistant nanofiltration for alcohol-based system. *Journal of membrane science* **2016,** *515*, 175-188.
12. Hao, L.; Zhang, H.; Wu, X.; Zhang, J.; Wang, J.; Li, Y., Novel thin-film nanocomposite membranes filled with multi-functional $Ti_3C_2T_x$ nanosheets for task-specific solvent transport. *Composites Part A: Applied Science and Manufacturing* **2017,** *100*, 139-149.
13. Wu, X.; Chen, Y.; Li, W.; Chen, C.; Zhang, J.; Wang, J., Heterostructured membranes with selective solvent-capture coatings and low-resistance 2D nanochannels for efficient mixed solvent separation. *Separation Purification Technology* **2022,** *283*, 120217.
14. Wu, X.; Cui, X.; Wu, W.; Wang, J.; Li, Y.; Jiang, Z., Elucidating ultrafast molecular permeation through well-defined 2D nanochannels of lamellar membranes. *Angewandte Chemie* **2019,** *131* (51), 18695-18700.
15. Wu, X.; Ding, M.; Xu, H.; Yang, W.; Zhang, K.; Tian, H.; Wang, H.; Xie, Z., Scalable $Ti_3C_2T_x$ MXene Interlayered Forward Osmosis Membranes for Enhanced Water Purification and Organic Solvent Recovery. *ACS nano* **2020,** *14* (7), 9125-9135.
16. Xing, Y.; Akonkwa, G.; Liu, Z.; Ye, H.; Han, K., Crumpled two-dimensional $Ti_3C_2T_x$ MXene lamellar membranes for solvent permeation and separation. *ACS Applied Nano Materials* **2020,** *3* (2), 1526-1534.
17. Shao, D.-D.; Zhang, Q.; Wang, L.; Wang, Z.-Y.; Jing, Y.-X.; Cao, X.-L.; Zhang, F.; Sun, S.-P., Enhancing interfacial adhesion of MXene nanofiltration membranes via pillaring carbon nanotubes for pressure and solvent stable molecular sieving. *Journal of Membrane Science* **2021,** *623*, 119033.
18. Shen, Y.; Yao, A.; Li, J.; Hua, D.; Tan, K. B.; Zhan, G.; Rao, X., Dispersive two-dimensional MXene via potassium fulvic acid for mixed matrix membranes with enhanced organic solvent nanofiltration performance. *Journal of Membrane Science* **2022**, 121168.

19. Liu, G.; Shen, J.; Ji, Y.; Liu, Q.; Liu, G.; Yang, J.; Jin, W., Two-dimensional Ti_2CT_x MXene membranes with integrated and ordered nanochannels for efficient solvent dehydration. *Journal of Materials Chemistry A* **2019,** *7* (19), 12095-12104.
20. Liu, G.; Liu, S.; Ma, K.; Wang, H.; Wang, X.; Liu, G.; Jin, W., Polyelectrolyte Functionalized Ti_2CT_x MXene Membranes for Pervaporation Dehydration of Isopropanol/Water Mixtures. *Industrial & Engineering Chemistry Research* **2020,** *59* (10), 4732-4741.
21. Xu, Z.; Liu, G.; Ye, H.; Jin, W.; Cui, Z., Two-dimensional MXene incorporated chitosan mixed-matrix membranes for efficient solvent dehydration. *Journal of membrane science* **2018,** *563*, 625-632.
22. Li, S.; Dai, J.; Geng, X.; Li, J.; Li, P.; Lei, J.; Wang, L.; He, J., Highly selective sodium alginate mixed-matrix membrane incorporating multi-layered MXene for ethanol dehydration. *Separation and Purification Technology* **2020,** *235*, 116206.
23. Cai, W.; Cheng, X.; Chen, X.; Li, J.; Pei, J., Poly (vinyl alcohol)-Modified Membranes by $Ti_3C_2T_x$ for Ethanol Dehydration via Pervaporation. *ACS omega* **2020,** *5* (12), 6277-6287.
24. Yang, G.; Xie, Z.; Thornton, A. W.; Doherty, C. M.; Ding, M.; Xu, H.; Cran, M.; Ng, D.; Gray, S., Ultrathin poly (vinyl alcohol)/MXene nanofilm composite membrane with facile intrusion-free construction for pervaporative separations. *Journal of Membrane Science* **2020,** *614*, 118490.
25. Wu, Y.; Ding, L.; Lu, Z.; Deng, J.; Wei, Y., Two-dimensional MXene membrane for ethanol dehydration. *Journal of Membrane Science* **2019,** *590*, 117300.
26. Ding, L.; Wei, Y.; Wang, Y.; Chen, H.; Caro, J.; Wang, H., A two-dimensional lamellar membrane: MXene nanosheet stacks. *Angewandte Chemie* **2017,** *129* (7), 1851-1855.
27. Han, R.; Ma, X.; Xie, Y.; Teng, D.; Zhang, S., Preparation of a new 2D MXene/PES composite membrane with excellent hydrophilicity and high flux. *Rsc Advances* **2017,** *7* (89), 56204-56210.
28. Zhang, S.; Liao, S.; Qi, F.; Liu, R.; Xiao, T.; Hu, J.; Li, K.; Wang, R.; Min, Y., Direct deposition of two-dimensional MXene nanosheets on commercially available filter for fast and efficient dye removal. *Journal of hazardous materials* **2020,** *384*, 121367.
29. Kim, J. H.; Park, G. S.; Kim, Y.-J.; Choi, E.; Kang, J.; Kwon, O.; Kim, S. J.; Cho, J. H.; Kim, D. W., Large-Area $Ti_3C_2T_x$-MXene Coating: Toward Industrial-Scale Fabrication and Molecular Separation. *ACS nano* **2021,** *15* (5), 8860-8869.
30. Ren, C. E.; Alhabeb, M.; Byles, B. W.; Zhao, M.-Q.; Anasori, B.; Pomerantseva, E.; Mahmoud, K. A.; Gogotsi, Y., Voltage-gated ions sieving through 2D MXene $Ti_3C_2T_x$ membranes. *ACS Applied Nano Materials* **2018,** *1* (7), 3644-3652.
31. Li, S.; Gu, W.; Sun, Y.; Zou, D.; Jing, W., Perforative pore formation on nanoplates for 2D porous MXene membranes via H_2O_2 mild etching. *Ceramics International* **2021,** *47* (21), 29930-29940.
32. Li, J.; Xu, C.; Long, J.; Ding, Z.; Yuan, R.; Li, Z., Lamellar MXene Nanofiltration Membranes for Electrostatic Modulation of Molecular Permeation: Implications for Fine Separation. *ACS Applied Nano Materials* **2022**.
33. Xiang, J.; Wang, X.; Ding, M.; Tang, X.; Zhang, S.; Zhang, X.; Xie, Z., The role of lateral size of MXene nanosheets in membrane filtration of dyeing wastewater: Membrane characteristic and performance. *Chemosphere* **2022,** *294*, 133728.
34. Yousaf, T.; Areeb, A.; Murtaza, M.; Munir, A.; Khan, Y.; Waseem, A., Silane-Grafted MXene ($Ti_3C_2T_X$) Membranes for Enhanced Water Purification Performance. *ACS Omega* **2022**.
35. Han, R.; Xie, Y.; Ma, X., Crosslinked P84 copolyimide/MXene mixed matrix membrane with excellent solvent resistance and permselectivity. *Chinese Journal of Chemical Engineering* **2019,** *27* (4), 877-883.
36. Pandey, R. P.; Rasheed, P. A.; Gomez, T.; Azam, R. S.; Mahmoud, K. A., A fouling-resistant mixed-matrix nanofiltration membrane based on covalently cross-linked $Ti_3C_2T_X$ (MXene)/cellulose acetate. *Journal of Membrane Science* **2020**, 118139.
37. Krecker, M. C.; Bukharina, D.; Hatter, C. B.; Gogotsi, Y.; Tsukruk, V. V., Bioencapsulated MXene flakes for enhanced stability and composite precursors. *Advanced Functional Materials* **2020,** *30* (43), 2004554.

38. Li, Y.; Dai, R.; Zhou, H.; Li, X.; Wang, Z., Aramid Nanofiber Membranes Reinforced by MXene Nanosheets for Recovery of Dyes from Textile Wastewater. *ACS Applied Nano Materials* **2021,** *4* (6), 6328-6336.
39. Yi, M.; Héraly, F.; Chang, J.; Kheirabad, A. K.; Yuan, J.; Wang, Y.; Zhang, M., A transport channel-regulated MXene membrane via organic phosphonic acids for efficient water permeation. *Chemical Communications* **2021,** *57* (51), 6245-6248.
40. Tong, X.; Liu, S.; Qu, D.; Gao, H.; Yan, L.; Chen, Y.; Crittenden, J., Tannic acid-metal complex modified MXene membrane for contaminants removal from water. *Journal of Membrane Science* **2021,** *622*, 119042.
41. Lin, Q.; Liu, Y.; Zeng, G.; Li, X.; Wang, B.; Cheng, X.; Sengupta, A.; Yang, X.; Feng, Z., Bionics inspired modified two-dimensional MXene composite membrane for high-throughput dye separation. *Journal of Environmental Chemical Engineering* **2021,** *9* (4), 105711.
42. Yi, M.; Wang, M.; Wang, Y.; Wang, Y.; Chang, J.; Kheirabad, A. K.; He, H.; Yuan, J.; Zhang, M., Poly (ionic liquid)-Armored MXene Membrane: Interlayer Engineering for Facilitated Water Transport. *Angewandte Chemie* **2022**, e202202515.
43. Yao, Y.-y.; Wang, T.; Wu, L.-g.; Chen, H.-l., PES mixed-matrix membranes incorporating ZIF-8@ MXene nanocomposite for the efficient dye/salt separation. *Desalination* **2022,** *543*, 116116.
44. Hu, W.; Xie, L.; Zeng, H., Novel sodium alginate-assisted MXene nanosheets for ultrahigh rejection of multiple cations and dyes. *Journal of colloid and interface science* **2020,** *568*, 36-45.
45. Liu, T.; Liu, X.; Graham, N.; Yu, W.; Sun, K., Two-dimensional MXene incorporated graphene oxide composite membrane with enhanced water purification performance. *Journal of Membrane Science* **2020,** *593*, 117431.
46. Pandey, R. P.; Rasool, K.; Madhavan, V. E.; Aïssa, B.; Gogotsi, Y.; Mahmoud, K. A., Ultrahigh-flux and fouling-resistant membranes based on layered silver/MXene ($Ti_3C_2T_x$) nanosheets. *Journal of Materials Chemistry A* **2018,** *6* (8), 3522-3533.
47. He, S.; Zhan, Y.; Hu, J.; Zhang, G.; Zhao, S.; Feng, Q.; Yang, W., Chemically stable two-dimensional MXene@ UIO-66-$(COOH)_2$ composite lamellar membrane for multi-component pollutant-oil-water emulsion separation. *Composites Part B: Engineering* **2020**, 108188.
48. Feng, X.; Yu, Z.; Long, R.; Sun, Y.; Wang, M.; Li, X.; Zeng, G., Polydopamine intimate contacted two-dimensional/two-dimensional ultrathin nylon basement membrane supported RGO/PDA/MXene composite material for oil-water separation and dye removal. *Separation and Purification Technology* **2020**, 116945.
49. Long, Q.; Zhao, S.; Chen, J.; Zhang, Z.; Qi, G.; Liu, Z.-Q., Self-assembly enabled nano-intercalation for stable high-performance MXene membranes. *Journal of Membrane Science* **2021,** *635*, 119464.
50. Sun, Y.; Xu, D.; Li, S.; Cui, L.; Zhuang, Y.; Xing, W.; Jing, W., Assembly of multidimensional MXene-carbon nanotube ultrathin membranes with an enhanced anti-swelling property for water purification. *Journal of Membrane Science* **2021,** *623*, 119075.
51. Zeng, G.; Lin, Q.; Wei, K.; Liu, Y.; Zheng, S.; Zhan, Y.; He, S.; Patra, T.; Chiao, Y.-H., High-performing composite membrane based on dopamine-functionalized graphene oxide incorporated two-dimensional MXene nanosheets for water purification. *Journal of Materials Science* **2021,** *56* (11), 6814-6829.
52. Ding, M.; Xu, H.; Chen, W.; Kong, Q.; Lin, T.; Tao, H.; Zhang, K.; Liu, Q.; Zhang, K.; Xie, Z., Construction of a hierarchical carbon nanotube/MXene membrane with distinct fusiform channels for efficient molecular separation. *Journal of Materials Chemistry A* **2020,** *8* (43), 22666-22673.
53. Gong, X.; Zhang, G.; Dong, H.; Wang, H.; Nie, J.; Ma, G., Self-assembled hierarchical heterogeneous MXene/COF membranes for efficient dye separations. *Journal of Membrane Science* **2022**, 120667.
54. Huang, H.; Xu, Y.; Lu, Z.; Zhang, A.; Zhang, D.; Xue, H.; Dong, P.; Zhang, J.; Goto, T., Highly permeable and dye-rejective nanofiltration membranes of TiO_2 and Bi_2S_3 double-embedded $Ti_3C_2T_x$ with a visible-light-induced self-cleaning ability. *Journal of Materials Research Technology* **2022,** *18*, 4156-4168.

55. Tao, M.-j.; Cheng, S.-Q.; Han, X.-L.; Yi, F.; Li, R.-H.; Rong, Y.; Sun, Y.; Liu, Y., Alignment of MXene based membranes to enhance water purification. *Journal of Membrane Science* **2022,** *662*, 120965.
56. Zhang, P.; Zhang, Y.; Wang, L.; Qiu, K.; Tang, X.; Gibson, J. K.; Liu, X.; Mei, L.; An, S.; Huang, Z., Bioinspired Macrocyclic Molecule Supported Two-Dimensional Lamellar Membrane with Robust Interlayer Structure for High-Efficiency Nanofiltration. *Advanced Science* **2023,** *10* (5), 2206516.
57. Pandey, R. P.; Rasool, K.; Abdul Rasheed, P.; Mahmoud, K. A., Reductive sequestration of toxic bromate from drinking water using lamellar two-dimensional $Ti_3C_2T_X$ (MXene). *ACS Sustainable Chemistry & Engineering* **2018,** *6* (6), 7910-7917.
58. Shahzad, A.; Nawaz, M.; Moztahida, M.; Jang, J.; Tahir, K.; Kim, J.; Lim, Y.; Vassiliadis, V. S.; Woo, S. H.; Lee, D. S., $Ti_3C_2T_x$ MXene core-shell spheres for ultrahigh removal of mercuric ions. *Chemical Engineering Journal* **2019,** *368*, 400-408.
59. Dong, X.; Wang, Y.; Jia, M.; Niu, Z.; Cai, J.; Yu, X.; Ke, X.; Yao, J.; Zhang, X., Sustainable and scalable in-situ synthesis of hydrochar-wrapped Ti_3AlC_2-derived nanofibers as adsorbents to remove heavy metals. *Bioresource technology* **2019,** *282*, 222-227.
60. Wang, L.; Tao, W.; Yuan, L.; Liu, Z.; Huang, Q.; Chai, Z.; Gibson, J. K.; Shi, W., Rational control of the interlayer space inside two-dimensional titanium carbides for highly efficient uranium removal and imprisonment. *Chemical Communications* **2017,** *53* (89), 12084-12087.
61. Jun, B.-M.; Her, N.; Park, C. M.; Yoon, Y., Effective removal of Pb (ii) from synthetic wastewater using $Ti_3C_2T_x$ MXene. *Environmental Science: Water Research & Technology* **2020,** *6* (1), 173-180.
62. Feng, Y.; Wang, H.; Xu, J.; Du, X.; Cheng, X.; Du, Z.; Wang, H., Fabrication of MXene/PEI functionalized sodium alginate aerogel and its excellent adsorption behavior for Cr (VI) and Congo Red from aqueous solution. *Journal of Hazardous Materials* **2021,** *416*, 125777.
63. Jin, L.; Chai, L.; Yang, W.; Wang, H.; Zhang, L., Two-dimensional titanium carbides ($Ti_3C_2T_x$) functionalized by poly (m-phenylenediamine) for efficient adsorption and reduction of hexavalent chromium. *International journal of environmental research and public health* **2020,** *17* (1), 167.
64. Ying, Y.; Liu, Y.; Wang, X.; Mao, Y.; Cao, W.; Hu, P.; Peng, X., Two-dimensional titanium carbide for efficiently reductive removal of highly toxic chromium (VI) from water. *ACS applied materials & interfaces* **2015,** *7* (3), 1795-1803.
65. Kong, A.; Sun, Y.; Peng, M.; Gu, H.; Fu, Y.; Zhang, J.; Li, W., Amino-functionalized MXenes for efficient removal of Cr (VI). *Colloids and Surfaces A: Physicochemical and Engineering Aspects* **2021,** *617*, 126388.
66. Zou, G.; Guo, J.; Peng, Q.; Zhou, A.; Zhang, Q.; Liu, B., Synthesis of urchin-like rutile titania carbon nanocomposites by iron-facilitated phase transformation of MXene for environmental remediation. *Journal of Materials Chemistry A* **2016,** *4* (2), 489-499.
67. He, L.; Huang, D.; He, Z.; Yang, X.; Yue, G.; Zhu, J.; Astruc, D.; Zhao, P., Nanoscale zero-valent iron intercalated 2D titanium carbides for removal of Cr (VI) in aqueous solution and the mechanistic aspect. *Journal of hazardous materials* **2020,** *388*, 121761.
68. Karthikeyan, P.; Ramkumar, K.; Pandi, K.; Fayyaz, A.; Meenakshi, S.; Park, C. M., Effective removal of Cr (VI) and methyl orange from the aqueous environment using two-dimensional (2D) $Ti_3C_2T_x$ MXene nanosheets. *Ceramics International* **2020**.
69. Xie, X.; Chen, C.; Zhang, N.; Tang, Z.-R.; Jiang, J.; Xu, Y.-J., Microstructure and surface control of MXene films for water purification. *Nature Sustainability* **2019,** *2* (9), 856-862.
70. Tang, Y.; Yang, C.; Que, W., A novel two-dimensional accordion-like titanium carbide (MXene) for adsorption of Cr (VI) from aqueous solution. *Journal of Advanced Dielectrics* **2018,** *8* (05), 1850035.
71. Khan, A. R.; Awan, S. K.; Husnain, S. M.; Abbas, N.; Anjum, D. H.; Abbas, N.; Benaissa, M.; Mirza, C. R.; Mujtaba-ul-Hassan, S.; Shahzad, F., 3D Flower like δ-MnO_2/MXene Nano-Hybrids for the Removal of Hexavalent Cr from Wastewater. *Ceramics International* **2021**.
72. Yang, G.; Hu, X.; Liang, J.; Huang, Q.; Dou, J.; Tian, J.; Deng, F.; Liu, M.; Zhang, X.; Wei, Y., Surface functionalization of MXene with chitosan through in-situ formation of polyimidazoles and its adsorption properties. *Journal of Hazardous Materials* **2021,** *419*, 126220.

73. Wan, H.; Nan, L.; Geng, H.; Zhang, W.; Shi, H., Green Synthesis of A Novel MXene–CS Composite Applied in Treatment of Cr (VI) Contaminated Aqueous Solution. *Processes* **2021,** *9* (3), 524.
74. Lv, Y.; Chang, K.; Wu, H.; Fang, P.; Chen, C.; Liao, Q., Highly efficient scavenging of Cr (VI) by two-dimensional titanium carbide nanosheets: kinetics, isotherms and thermodynamics analysis. *Water Science Technology* **2021,** *84* (9), 2446-2456.
75. Zhang, G.; Wang, T.; Xu, Z.; Liu, M.; Shen, C.; Meng, Q., Synthesis of amino-functionalized $Ti_3C_2T_x$ MXene by alkalization-grafting modification for efficient lead adsorption. *Chemical Communications* **2020,** *56* (76), 11283-11286.
76. Elumalai, S.; Yoshimura, M.; Ogawa, M., Simultaneous delamination and rutile formation on the surface of $Ti_3C_2T_x$ MXene for copper adsorption. *Chemistry–An Asian Journal* **2020,** *15* (7), 1044-1051.
77. Dong, Y.; Sang, D.; He, C.; Sheng, X.; Lei, L., MXene/alginate composites for lead and copper ion removal from aqueous solutions. *RSC advances* **2019,** *9* (50), 29015-29022.
78. Shahzad, A.; Rasool, K.; Miran, W.; Nawaz, M.; Jang, J.; Mahmoud, K. A.; Lee, D. S., Two-dimensional $Ti_3C_2T_x$ MXene nanosheets for efficient copper removal from water. *ACS Sustainable Chemistry & Engineering* **2017,** *5* (12), 11481-11488.
79. Gan, D.; Huang, Q.; Dou, J.; Huang, H.; Chen, J.; Liu, M.; Wen, Y.; Yang, Z.; Zhang, X.; Wei, Y., Bioinspired functionalization of MXenes ($Ti_3C_2T_X$) with amino acids for efficient removal of heavy metal ions. *Applied Surface Science* **2020,** *504*, 144603.
80. Ren, J.; Zhu, Z.; Qiu, Y.; Yu, F.; Zhou, T.; Ma, J.; Zhao, J., Enhanced adsorption performance of alginate/MXene/$CoFe_2O_4$ for antibiotic and heavy metal under rotating magnetic field. *Chemosphere* **2021,** *284*, 131284.
81. Zhang, K.-N.; Wang, C.-Z.; Lü, Q.-F.; Chen, M.-H., Enzymatic hydrolysis lignin functionalized $Ti_3C_2T_x$ nanosheets for effective removal of MB and Cu^{2+} ions. *International Journal of Biological Macromolecules* **2022,** *209*, 680-691.
82. Feng, X.; Yu, Z.; Long, R.; Li, X.; Shao, L.; Zeng, H.; Zeng, G.; Zuo, Y., Self-assembling 2D/2D (MXene/LDH) materials achieve ultra-high adsorption of heavy metals Ni^{2+} through terminal group modification. *Separation and Purification Technology* **2020,** *253*, 117525.
83. Gu, P.; Xing, J.; Wen, T.; Zhang, R.; Wang, J.; Zhao, G.; Hayat, T.; Ai, Y.; Lin, Z.; Wang, X., Experimental and theoretical calculation investigation on efficient Pb (II) adsorption on etched Ti_3AlC_2 nanofibers and nanosheets. *Environmental Science: Nano* **2018,** *5* (4), 946-955.
84. Du, Y.; Yu, B.; Wei, L.; Wang, Y.; Zhang, X.; Ye, S., Efficient removal of Pb (II) by $Ti_3C_2T_x$ powder modified with a silane coupling agent. *Journal of Materials Science* **2019,** *54* (20), 13283-13297.
85. Peng, Q.; Guo, J.; Zhang, Q.; Xiang, J.; Liu, B.; Zhou, A.; Liu, R.; Tian, Y., Unique lead adsorption behavior of activated hydroxyl group in two-dimensional titanium carbide. *Journal of the American Chemical Society* **2014,** *136* (11), 4113-4116.
86. Wang, S.; Liu, Y.; Lü, Q.-F.; Zhuang, H., Facile preparation of biosurfactant-functionalized Ti_2CT_X MXene nanosheets with an enhanced adsorption performance for Pb (II) ions. *Journal of Molecular Liquids* **2020,** *297*, 111810.
87. Qin, Z.; Deng, H.; Huang, R.; Tong, S., 3D MXene hybrid architectures for the cold-resistant, rapid and selective capture of precious metals from electronic waste and mineral. *Chemical Engineering Journal* **2022,** *428*, 132493.
88. Wang, C.; Cheng, R.; Hou, P.-X.; Ma, Y.; Majeed, A.; Wang, X.; Liu, C., MXene-Carbon Nanotube Hybrid Membrane for Robust Recovery of Au from Trace-Level Solution. *ACS Applied Materials & Interfaces* **2020,** *12* (38), 43032-43041.
89. Fu, K.; Liu, X.; Yu, D.; Luo, J.; Wang, Z.; Crittenden, J. C., Highly Efficient and Selective Hg (II) Removal from Water Using Multilayered $Ti_3C_2O_x$ MXene via Adsorption Coupled with Catalytic Reduction Mechanism. *Environmental Science & Technology* **2020,** *54* (24), 16212-16220.
90. Shahzad, A.; Jang, J.; Lim, S.-R.; Lee, D. S., Unique selectivity and rapid uptake of molybdenum-disulfide-functionalized MXene nanocomposite for mercury adsorption. *Environmental Research* **2020,** *182*, 109005.

91. Shahzad, A.; Rasool, K.; Miran, W.; Nawaz, M.; Jang, J.; Mahmoud, K. A.; Lee, D. S., Mercuric ion capturing by recoverable titanium carbide magnetic nanocomposite. *Journal of hazardous materials* **2018,** *344*, 811-818.
92. Hu, X.; Chen, C.; Zhang, D.; Xue, Y., Kinetics, isotherm and chemical speciation analysis of Hg (Ⅱ) adsorption over oxygen-containing MXene adsorbent. *Chemosphere* **2021,** *278*, 130206.
93. Shahzad, A.; Rasool, K.; Iqbal, J.; Jang, J.; Lim, Y.; Kim, B.; Oh, J.-M.; Lee, D. S., MXsorption of mercury: Exceptional reductive behavior of titanium carbide/carbonitride MXenes. *Environmental Research* **2022,** *205*, 112532.
94. Mu, W.; Du, S.; Li, X.; Yu, Q.; Wei, H.; Yang, Y.; Peng, S., Removal of radioactive palladium based on novel 2D titanium carbides. *Chemical Engineering Journal* **2019,** *358*, 283-290.
95. Fard, A. K.; Mckay, G.; Chamoun, R.; Rhadfi, T.; Preud'Homme, H.; Atieh, M. A., Barium removal from synthetic natural and produced water using MXene as two dimensional (2-D) nanosheet adsorbent. *Chemical engineering journal* **2017,** *317*, 331-342.
96. Jun, B.-M.; Park, C. M.; Heo, J.; Yoon, Y., Adsorption of Ba^{2+} and Sr^{2+} on $Ti_3C_2T_x$ MXene in model fracking wastewater. *Journal of environmental management* **2020,** *256*, 109940.
97. Mu, W.; Du, S.; Yu, Q.; Li, X.; Wei, H.; Yang, Y., Improving barium ion adsorption on two-dimensional titanium carbide by surface modification. *Dalton Transactions* **2018,** *47* (25), 8375-8381.
98. Shahzad, A.; Moztahida, M.; Tahir, K.; Kim, B.; Jeon, H.; Ghani, A. A.; Maile, N.; Jang, J.; Lee, D. S., Highly effective prussian blue-coated MXene aerogel spheres for selective removal of cesium ions. *Journal of Nuclear Materials* **2020,** *539*, 152277.
99. Jun, B.-M.; Jang, M.; Park, C. M.; Han, J.; Yoon, Y., Selective adsorption of Cs^+ by MXene ($Ti_3C_2T_x$) from model low-level radioactive wastewater. *Nuclear Engineering and Technology* **2020,** *52* (6), 1201-1207.
100. Khan, A. R.; Husnain, S. M.; Shahzad, F.; Mujtaba-ul-Hassan, S.; Mehmood, M.; Ahmad, J.; Mehran, M. T.; Rahman, S., Two-dimensional transition metal carbide ($Ti_3C_2T_x$) as an efficient adsorbent to remove cesium (Cs^+). *Dalton Transactions* **2019,** *48* (31), 11803-11812.
101. Rethinasabapathy, M.; Hwang, S. K.; Kang, S.-M.; Roh, C.; Huh, Y. S., Amino-functionalized POSS nanocage-intercalated titanium carbide ($Ti_3C_2T_x$) MXene stacks for efficient cesium and strontium radionuclide sequestration. *Journal of Hazardous Materials* **2021,** *418*, 126315.
102. Zhang, P.; Wang, L.; Yuan, L.-Y.; Lan, J.-H.; Chai, Z.-F.; Shi, W.-Q., Sorption of Eu (III) on MXene-derived titanate structures: the effect of nano-confined space. *Chemical Engineering Journal* **2019,** *370*, 1200-1209.
103. Zhang, P.; Wang, L.; Du, K.; Wang, S.; Huang, Z.; Yuan, L.; Li, Z.; Wang, H.; Zheng, L.; Chai, Z., Effective removal of U (VI) and Eu (III) by carboxyl functionalized MXene nanosheets. *Journal of hazardous materials* **2020,** *396*, 122731.
104. Yan, J.; Liu, H. J.; Xie, L.; Liu, Z.; Liu, P. F.; Wen, H. X., Europium (III) removal from aqueous solution using citric acid modified alkalized Mxene as an adsorbent. *Journal of Radioanalytical and Nuclear Chemistry* **2022,** *331* (2), 1063-1073.
105. Gu, P.; Zhang, S.; Ma, R.; Sun, M.; Wang, S.; Wen, T.; Wang, X., Layered double hydroxides nanosheets in-situ anchored on ultrathin MXenes for enhanced U (VI) and Eu (III) trapping: Excavating from selectivity to mechanism. *Separation and Purification Technology* **2022,** *288*, 120641.
106. Zhang, D.; Zhao, B.; Liu, L.; Tang, H.; Wang, X.; Yu, S., Insights into enhanced elimination of U (VI) and Eu (III) by amidoxime-functionalized $Ti_3C_2T_x$ MXenes. *Separation and Purification Technology* **2022,** *294*, 121179.
107. Huang, H.; Sha, X.; Cui, Y.; Sun, S.; Huang, H.; He, Z.; Liu, M.; Zhou, N.; Zhang, X.; Wei, Y., Highly efficient removal of iodine ions using MXene-PDA-Ag_2O_x composites synthesized by mussel-inspired chemistry. *Journal of Colloid and interface Science* **2020,** *567*, 190-201.
108. Sun, S.; Sha, X.; Liang, J.; Yang, G.; Hu, X.; He, Z.; Liu, M.; Zhou, N.; Zhang, X.; Wei, Y., Rapid synthesis of polyimidazole functionalized MXene via microwave-irradiation assisted multi-component reaction and its iodine adsorption performance. *Journal of Hazardous Materials* **2021,** *420*, 126580.

109. Wang, L.; Song, H.; Yuan, L.; Li, Z.; Zhang, P.; Gibson, J. K.; Zheng, L.; Wang, H.; Chai, Z.; Shi, W., Effective removal of anionic Re (VII) by surface-modified Ti_2CT_x MXene nanocomposites: implications for Tc (VII) sequestration. *Environmental science & technology* **2019,** *53* (7), 3739-3747.
110. Li, S.; Wang, L.; Peng, J.; Zhai, M.; Shi, W., Efficient thorium (IV) removal by two-dimensional Ti_2CT_x MXene from aqueous solution. *Chemical Engineering Journal* **2019,** *366*, 192-199.
111. Wang, L.; Tao, W.; Ma, E.; Li, Z.; Ren, P.; Zhang, Y.; Liu, Z.; Yuan, L.; Shi, W., Thorium (IV) adsorption onto multilayered $Ti_3C_2T_x$ MXene: a batch, X-ray diffraction and EXAFS combined study. *Journal of Synchrotron Radiation* **2021,** *28* (6), 1709-1719.
112. Wang, S.; Wang, L.; Li, Z.; Zhang, P.; Du, K.; Yuan, L.; Ning, S.; Wei, Y.; Shi, W., Highly efficient adsorption and immobilization of U (VI) from aqueous solution by alkalized MXene-supported nanoscale zero-valent iron. *Journal of Hazardous Materials* **2021,** *408*, 124949.
113. Wang, L.; Song, H.; Yuan, L.; Li, Z.; Zhang, Y.; Gibson, J. K.; Zheng, L.; Chai, Z.; Shi, W., Efficient U (VI) reduction and sequestration by Ti_2CT_x MXene. *Environmental science & technology* **2018,** *52* (18), 10748-10756.
114. Zhang, P.; Wang, L.; Huang, Z.; Yu, J.; Li, Z.; Deng, H.; Yin, T.; Yuan, L.; Gibson, J. K.; Mei, L., Aryl diazonium-assisted amidoximation of MXene for boosting water stability and uranyl sequestration via electrochemical sorption. *ACS applied materials & interfaces* **2020,** *12* (13), 15579-15587.
115. Wang, L.; Yuan, L.; Chen, K.; Zhang, Y.; Deng, Q.; Du, S.; Huang, Q.; Zheng, L.; Zhang, J.; Chai, Z., Loading actinides in multilayered structures for nuclear waste treatment: the first case study of uranium capture with vanadium carbide MXene. *ACS Applied Materials & Interfaces* **2016,** *8* (25), 16396-16403.
116. Zhao, W.; Chi, H.; Zhang, X.; Wang, Y.; Li, T., Cellulose/silsesquioxane grafted $Ti_3C_2T_x$ MXene for synergistically enhanced adsorption of uranium. *Colloids and Surfaces A: Physicochemical and Engineering Aspects* **2022,** *650*, 129610.
117. Chen, Z.; Xu, X.; Ding, Z.; Wang, K.; Sun, X.; Lu, T.; Konarova, M.; Eguchi, M.; Shapter, J. G.; Pan, L., Ti_3C_2 MXenes-derived $NaTi_2(PO_4)_3$/MXene nanohybrid for fast and efficient hybrid capacitive deionization performance *Chemical Engineering Journal* **2020**, 127148.
118. Shen, X.; Xiong, Y.; Hai, R.; Yu, F.; Ma, J., All-MXene-based integrated membrane electrode constructed using $Ti_3C_2T_x$ as an intercalating agent for high-performance desalination. *Environmental Science & Technology* **2020,** *54* (7), 4554-4563.
119. Ma, J.; Cheng, Y.; Wang, L.; Dai, X.; Yu, F., Free-standing $Ti_3C_2T_x$ MXene film as binder-free electrode in capacitive deionization with an ultrahigh desalination capacity. *Chemical Engineering Journal* **2020,** *384*, 123329.
120. Bao, W.; Tang, X.; Guo, X.; Choi, S.; Wang, C.; Gogotsi, Y.; Wang, G., Porous cryo-dried MXene for efficient capacitive deionization. *Joule* **2018,** *2* (4), 778-787.
121. Amiri, A.; Chen, Y.; Teng, C. B.; Naraghi, M., Porous nitrogen-doped MXene-based electrodes for capacitive deionization. *Energy Storage Materials* **2020,** *25*, 731-739.
122. Anwer, S.; Anjum, D.; Luo, S.; Abbas, Y.; Li, B.; Iqbal, S.; Liao, K., 2D $Ti_3C_2T_x$ MXene nanosheets coated cellulose fibers based 3D nanostructures for efficient water desalination. *Chemical Engineering Journal 406*, 126827.
123. Guo, L.; Wang, X.; Leong, Z. Y.; Mo, R.; Sun, L.; Yang, H. Y., Ar plasma modification of 2D MXene $Ti_3C_2T_x$ nanosheets for efficient capacitive desalination. *FlatChem* **2018,** *8*, 17-24.
124. Chen, B.; Feng, A.; Deng, R.; Liu, K.; Yu, Y.; Song, L., MXene as a Cation-Selective Cathode Material for Asymmetric Capacitive Deionization. *ACS Applied Materials & Interfaces* **2020,** *12* (12), 13750-13758.
125. Srimuk, P.; Halim, J.; Lee, J.; Tao, Q.; Rosen, J.; Presser, V., Two-dimensional molybdenum carbide (MXene) with divacancy ordering for brackish and seawater desalination via cation and anion intercalation. *ACS Sustainable Chemistry & Engineering* **2018,** *6* (3), 3739-3747.
126. Srimuk, P.; Kaasik, F.; Krüner, B.; Tolosa, A.; Fleischmann, S.; Jäckel, N.; Tekeli, M. C.; Aslan, M.; Suss, M. E.; Presser, V., MXene as a novel intercalation-type pseudocapacitive cathode and anode for capacitive deionization. *Journal of Materials Chemistry A* **2016,** *4* (47), 18265-18271.

127. Feng, A.; Yu, Y.; Mi, L.; Yu, Y.; Song, L., Comparative study on electrosorptive behavior of NH_4HF_2-etched Ti_3C_2 and HF-etched Ti_3C_2 for capacitive deionization. *Ionics* **2019,** *25* (2), 727-735.
128. Torkamanzadeh, M.; Wang, L.; Zhang, Y.; Budak, Ö.; Srimuk, P.; Presser, V., MXene/activated carbon hybrid capacitive deionization for permselective ion removal at low and high salinity. *ACS Applied Materials & Interfaces* **2020**.
129. Agartan, L.; Hantanasirisakul, K.; Buczek, S.; Akuzum, B.; Mahmoud, K. A.; Anasori, B.; Gogotsi, Y.; Kumbur, E. C., Influence of operating conditions on the desalination performance of a symmetric pre-conditioned $Ti_3C_2T_x$-MXene membrane capacitive deionization system. *Desalination* **2020,** *477*, 114267.
130. Mansoor, N. E.; Diaz, L. A.; Shuck, C. E.; Gogotsi, Y.; Lister, T. E.; Estrada, D., Removal and recovery of ammonia from simulated wastewater using $Ti_3C_2T_x$ MXene in flow electrode capacitive deionization. *NPJ Clean Water* **2022,** *5* (1), 1-11.
